# OptiMouse: a comprehensive open source program for reliable detection and analysis of mouse body and nose positions

**DOI:** 10.1186/s12915-017-0377-3

**Published:** 2017-05-15

**Authors:** Yoram Ben-Shaul

**Affiliations:** Department of Medical Neurobiology, The Hebrew University, Faculty of Medicine, Jerusalem, Israel

**Keywords:** Rodent behavior, Position analysis, Preference tests, Video data

## Abstract

**Background:**

Accurate determination of mouse positions from video data is crucial for various types of behavioral analyses. While detection of body positions is straightforward, the correct identification of nose positions, usually more informative, is far more challenging. The difficulty is largely due to variability in mouse postures across frames.

**Results:**

Here, we present OptiMouse, an extensively documented open-source MATLAB program providing comprehensive semiautomatic analysis of mouse position data. The emphasis in OptiMouse is placed on minimizing errors in position detection. This is achieved by allowing application of multiple detection algorithms to each video, including custom user-defined algorithms, by selection of the optimal algorithm for each frame, and by correction when needed using interpolation or manual specification of positions.

**Conclusions:**

At a basic level, OptiMouse is a simple and comprehensive solution for analysis of position data. At an advanced level, it provides an open-source and expandable environment for a detailed analysis of mouse position data.

**Electronic supplementary material:**

The online version of this article (doi:10.1186/s12915-017-0377-3) contains supplementary material, which is available to authorized users.

## Background

Many studies of rodent behavior are based on the analysis of positional data. Such data can reveal place preference, attraction to particular stimuli, or other positional information such as speed and distance covered [1–6]. Analysis of videos of mouse behavior in arenas can be performed by manual scoring or by automated video analysis. While the first approach does not require any dedicated software, it is highly subjective, prone to error, and time consuming if conducted rigorously. Automated analysis of position data is objective and usually fast, but is often also not error free.

The challenge of position detection is not to identify body and nose positions in any single image, but rather to do so correctly for the vast majority of frames in any given movie. This is considerably harder for detection of nose as compared to body center coordinates. This distinction is important because it is the snout that indicates what a mouse is attending to. The difficulty of the image analysis problem varies from video to video. Decreased contrast between the mouse and the background, increased variability in mouse posture, frames that do not include the mouse in its entirety, as well as temporal and spatial inhomogeneity of the arena, are all factors that increase the likelihood of errors. Common procedures for detecting nose positions involve an initial step of automatic detection, followed by a reviewing of the detection algorithm’s performance. When errors are detected, they can often be manually corrected. The last stage can be very laborious and time consuming, making it difficult to detect all instances of erroneous detections. For example, a 10-minute movie with a 30 Hz frame rate, will include 18,000 frames. This is a large number of frames to monitor, let alone to correct.

Here, we describe a new open-source software, OptiMouse, designed for the analysis of positions of individual mice in a behavioral arena. OptiMouse is written in MATLAB and covers the entire sequence of mouse position data analysis, starting from a video and ending with processed measures of mouse position and movement data within an arena. Other programs for automated analysis of position data exist. Some of these are commercial products with a broad range of features (e.g., Ethovision, ANT-maze), others are freely available programs. Among these, several are designed for detection of particular features such as stretch or grooming [7, 8], exploratory behaviors [9–11], or social interactions [12, 13]. Other programs also include advanced algorithms for detecting patterns in behavior [14–16]. The motivation for creating yet another software program for this general problem is two-fold. First, OptiMouse was designed to provide highly accurate detection of positions, and to facilitate the process of reviewing and correcting, when needed, the automatically detected positions. As described below, many features in OptiMouse were designed with the objective of improving accuracy in position detection. Second, OptiMouse was developed with the goal of making the entire analysis process accessible and free. Working with OptiMouse involves graphical user interfaces, and does not require any programming knowledge. A detailed user manual accompanies the code and all detection stages can be controlled by the user.

Although it provides several algorithms for detection, OptiMouse does not rely on any single algorithm to detect positions under all scenarios. The key assumption behind OptiMouse is that, while usually there will not be a single set of parameters (a setting) that correctly identifies positions in all movie frames, a small number of settings will suffice to provide correct detection in the vast majority.

Building upon these assumptions, OptiMouse includes the following elements:

1. Application of multiple detection settings to a single session. In addition to several built-in algorithms, each with its own associated set of parameters, OptiMouse allows seamless integration of custom functions for body/nose detection into the user interface.
2. Efficient identification of frames with incorrect position detection. In addition to serially reviewing the video to identify problematic frames, OptiMouse allows simultaneous viewing of various features describing each of the frames. This ability facilitates identification of frames with incorrect position detection.
3. Methods for correcting detection in frames with errors. This includes the application of settings to single frames or to groups of frames, defining manual positions, and interpolating body and nose coordinates.

The ease of use, expandability, and performance of OptiMouse should make it useful both for occasional investigators of mouse behavior and for researchers that wish to apply a rigorous analysis of position data.

## Implementation

**Fig. 1.**
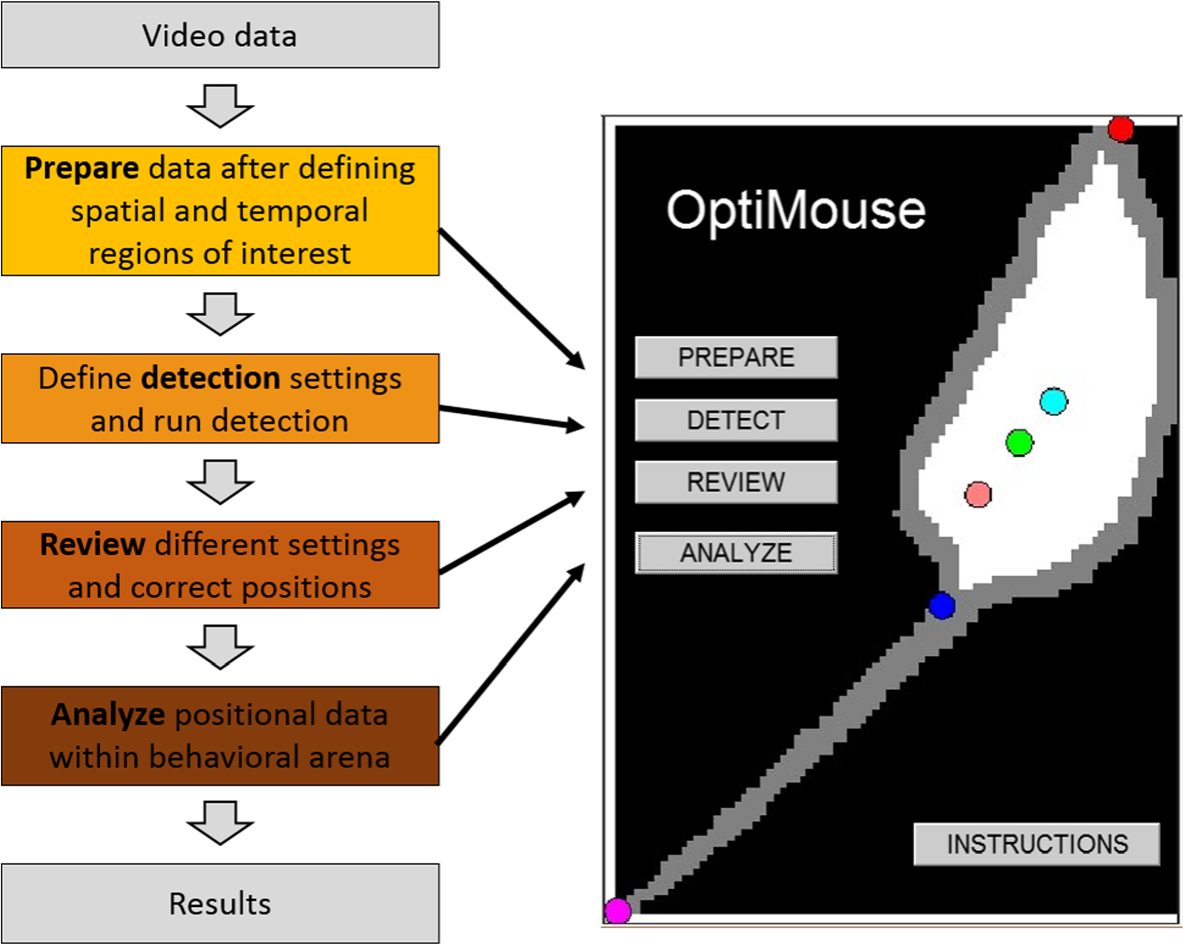
The main modules in OptiMouse. The left side shows a workflow of the main analysis stages. The right image shows the main OptiMouse interface. Each of the four buttons evokes a GUI for the corresponding stage

Analysis in OptiMouse involves four main stages (Fig. 1). In the first stage, Preparation, the user must specify the spatial and temporal regions of interest within the video file to define a session. Once defined, OptiMouse converts the raw video data into MATLAB files which contain only data relevant for each session. In the next stage, Detection, OptiMouse detects nose and body positions for each frame of the video. At the start of this stage, the user must specify one or more detection settings. Each setting is essentially an algorithm whose parameters are specified by the user. During the Detection stage, OptiMouse will calculate position data for each frame, according to each of the settings. The outcome of the Detection stage is the Position file which can be directly used for analysis. Analysis results can be displayed graphically or saved to a file (the Results file, in MATLAB data format). A key feature of OptiMouse is the optional reviewing stage, which allows the user to choose among the various detection settings or define manual settings for each frame. The reviewing stage also allows manual frame by frame annotation. The importance of implementing the reviewing stage depends on both the video quality and on the sensitivity of the analyses to errors in detection. Table 1 describes the key stages and the required user intervention in each. Table 2 provides a general overview of the key properties of OptiMouse, allowing users to determine if the software is suitable for their specific needs.

**Table 1:** Key stages in OptiMouse

Stage	Goal	Comments
Preparation	Define arena boundaries	The user must define the region of interest for analysis and provide actual arena dimensions.
Preparation	Conversion of raw video data to MATLAB files	Once arenas are defined, the user can initiate conversion Conversion can be run in batch mode, allowing definition of multiple arenas and then running conversion for all, in a single operation.
Detection	Determine optimal detection settings	The user may simply accept the default algorithm and initiate detection; however, in most cases, detection of body and nose positions can be significantly improved by small adjustments of the detection algorithm parameters and, often, by definition of multiple algorithms. In this stage, the movie is browsed to find detection settings that minimize errors in the detection of body and nose positions. The goal is to define a minimum number of algorithms so that at least one is appropriate for each frame of the movie. If multiple settings are defined, one of them must be specified as the default.
Detection	Running detection	During detection, OptiMouse finds mouse positions in each frame according to the settings defined by the user. Like conversion, detection can be time consuming and thus the option for a batch mode is provided. Detection settings can be defined for multiple sessions, and then run in a single batch operation. Note that the output of the detection stage, the position files, can be immediately used for analysis manually or by using the OptiMouse analysis interface.
Reviewing (optional)	Testing, and if needed, correcting, of the detection settings; annotation	If the stage is bypassed, then the default setting will be applied to all frames. The reviewing stage allows overriding of the default algorithm positions. Individual frames or groups of frames can be assigned with any of the non-default settings defined by the user, or set manually. OptiMouse provides multiple tools to browse the video or to locate individual frames with specific attributes. The impact of reviewing on the final position data depends on the number of errors associated with the default detection setting. A major effort has been made in OptiMouse to provide tools to efficiently locate problematic frames. Nevertheless, this stage may be time consuming. During reviewing, it is also possible to add annotation to the video.
Analysis	Derive meaningful behavioral parameters from the position data	Several analysis options are provided at this stage via the graphical user interface. Additionally, task-specific analyses can be implemented by analyzing the Results file. In this stage, the user can define zones (within the arena) and analyze position data with respect to these zones.

**Table 2:** Frequently asked questions concerning the key properties of OptiMouse

**What programs must be installed on my computer to run OptiMouse?**
MATLAB with the image processing tool box. OptiMouse has been tested on MATLAB releases 2015b and 2016a and 2016b. OptiMouse is not compatible with some older versions of MATLAB which use different coding conventions for graphical interfaces.
**Which operating systems can OptiMouse run on?**
OptiMouse was developed in Windows 8. However, it should be compatible with other operating systems that run MATLAB.
**Do I need to know MATLAB to run OptiMouse?**
Running OptiMouse requires a very basic familiarity with MATLAB. At the minimum, it is required to set the path and call the program from the command line. More advanced data analyses, and modifications and extension of the code, naturally require MATLAB programming skills.
**Which video files formats can OptiMouse use?**
OptiMouse was tested with the following formats: mp4, mpg, and wmv. However, any format supported by the MATLAB VideoReader object should be valid. The list of supported formats is obtained by typing “VideoReader.getFileFormats” on the MATLAB command line. On MATLAB 2016b running on a windows OS, this yields:
.asx - ASX File
.avi - AVI File
.m4v - MPEG-4 Video
.mj2 - Motion JPEG2000
.mov - QuickTime movie
.mp4 - MPEG-4
.mpg - MPEG-1
.wmv - Windows Media Video
**Is OptiMouse suitable for real time processing?**
No. OptiMouse is an offline analysis software.
**Is OptiMouse suitable for analyzing social interactions?**
No. OptiMouse is designed for analyzing the behavior of a single mouse.
**Which types of behavioral tests is OptiMouse suitable for?**
OptiMouse is suitable for any test that involves a single mouse in a stationary arena. Examples of standard tests that fall into this category are place preference tests, open field behavior, plus mazes, three-chamber tests.
**Does OptiMouse identify specific body regions?**
Yes. A key aspect in OptiMouse is the detection of body center and nose positions. Some of the detection algorithms in OptiMouse also detect the tail-end and tail-base as intermediate stages of nose detection. However, the coordinates of these positions are not used in other analyses.
**Can OptiMouse detect particular postures, such as rearing and grooming, or gait patterns?**
No. OptiMouse does not provide automatic detection of body postures. Manual annotation on a frame by frame basis is possible in OptiMouse.
**What detection algorithms are built into OptiMouse?**
OptiMouse includes several detection algorithms, each of which includes several parameters that can be modified by the user. All of them rely on a color contrast (after the movie has been transformed to grayscale) between the mouse and the arena. Most built-in algorithms employ “peeling” of the mouse image perimeter, which allows detection of the tail and then assists detection of the nose. A detailed explanation is provided in the user manual. In addition, OptiMouse allows incorporation of custom written detection functions.
**Are there any constraints on the coat color of the mouse?**
Yes. The coat color must be distinct from the arena’s background. Ideally, the entire mouse should be darker or lighter than the arena. Thus, a black mouse on a white arena or vice-versa are the ideal scenarios. However, if the mouse contains small patches of a different color on its body, this should not significantly impair detection. Current detection algorithms in OptiMouse will not perform well with a two-colored black and white animal on a gray background.
**I have an algorithm that works really well on my videos. Can I use it in OptiMouse?**
Yes. OptiMouse is designed to incorporate other detection algorithms. Custom algorithms are “declared” in one of the OptiMouse folders, and then are essentially incorporated into the user interface. Custom functions accept image data and other optional parameters, including user defined parameters. It is even possible to set user-defined parameters (which are not part of the current detection algorithm) graphically via the OptiMouse GUI. Custom written algorithms must, at minimum, return body and nose positions. See the user’s manual for a more detailed explanation of how to write and incorporate custom algorithms into OptiMouse.
**How long will it take me to obtain results from a video file?**
The answer obviously depends on many factors. Under ideal conditions, which require minimal user intervention, the entire procedure to process a 10 minute video may require about 20–30 minutes. The actual values also depend on video frame rate, resolution, and computer processing speeds. Most of the time is spent on automated processing not requiring any user input. Such processing can be performed in batch mode, so that the actual user time (with minimal user intervention) is a few minutes.
**Which stages require most user intervention?**
The two most time consuming stages are setting detection parameters and reviewing the video after detection has been performed. In both cases, the required time depends on the video quality. Videos in which the mouse is always easily separated from the arena facilitate both stages. Videos with variable conditions and poor image signal-to-noise ratios will require more tweaking of the detection settings. Setting detection parameters involves browsing the video and deciding on a number of detection algorithms. This typically requires a few minutes for each video (settings can be saved and applied to other movies if they share similar attributes, thus reducing the time required by the user). Reviewing of the movie can be a lengthy process and depends on the performance of the detection algorithms and the desired accuracy. The reviewing stage includes multiple tools to easily identify frames with erroneous detection, as well as frames in which the mouse is in particular parts of the arena, allowing the user to focus reviewing efforts on the frames that matter most.
**Can behavioral data be synchronized with other data (e.g., electrophysiological data)?**
OptiMouse does not include a built-in synchronizing signal. However, the Results file contains a frame by frame account of various parameters such as body and nose position, body angle, speed, presence in a certain zone, and occurrence of annotated events. The Results file also contains a time stamp for every frame so that, if the first video frame is synchronized with other non-video data, all other OptiMouse values can be aligned as well.
**Can I modify OptiMouse beyond the addition of custom detection algorithms?**
OptiMouse is stored in Github, with the hope that this will facilitate a community based development of the code. OptiMouse is written in MATLAB and individual code files (m-files) are annotated. The user manual provides a description of file formats, algorithms, and data conversions. The graphical user interface has been designed with the MATLAB GUIDE tool, which can be used to modify the existing interfaces and the code associated with various controls.
**Which analyses are implemented in the Analysis interface?**
In the analysis stage there is a distinction between zone dependent and zone independent analysis. Without defining zones, OptiMouse can generate graphical displays of positions (as tracks or heatmaps), speeds, and body angles as a function of time. If events have been annotated during the annotation stage, their total occurrence during the session or their distribution in time can also be plotted. In addition, the Analysis GUI allows the definition of zones (of arbitrary number and shapes within the arena). Once zones are derived, the analysis of positions and events can also be shown as a function of zone entries.
**Can I implement my own data analyses on positions detected by OptiMouse?**
Yes. The Position file contains frame by frame mouse positional data as well as user annotated events. The Results file also contains zone-related information. Both files are MATLAB data files (*.mat) which can be used for more complicated analysis. A detailed description of the Results file format is provided in the user manual. In addition, the user manual provides example code for the analysis of freezing episodes from the Results file.
**Does OptiMouse support batch processing of position data files?**
No. However, OptiMouse contains an option for adding tags to individual files. These tags then allow grouping of files for statistical comparison of groups of files. See the manual for more details on the use of “experiment tags”.
**How do I get more information about OptiMouse?**
OptiMouse includes a detailed user manual. For issues that are not covered in the manual, contact the corresponding author of this manuscript by email.

The manuscript describes the conceptual ideas behind the operation of OptiMouse, rather than the practical steps of working with the software. The latter, along with more complete descriptions of the features and algorithms implemented, are covered in a detailed user manual (Additional file 1). Although all stages are described, the emphasis here is placed on the stages that are unique to OptiMouse, namely the detection and the reviewing stages.

### Defining spatial and temporal regions of interest

**Fig. 2.**
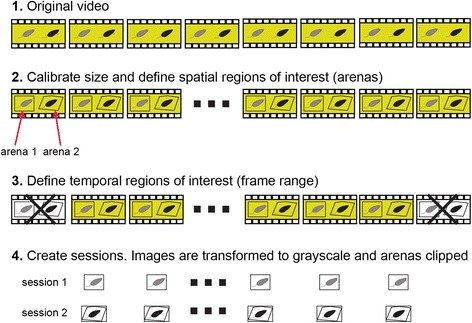
Schematic description of the session preparation process. Preparation involves spatial definitions of one or more arenas and size calibration as well as optional removal of irrelevant video sections. Video data for each of the sessions is converted to grayscale images

**Fig. 3.**
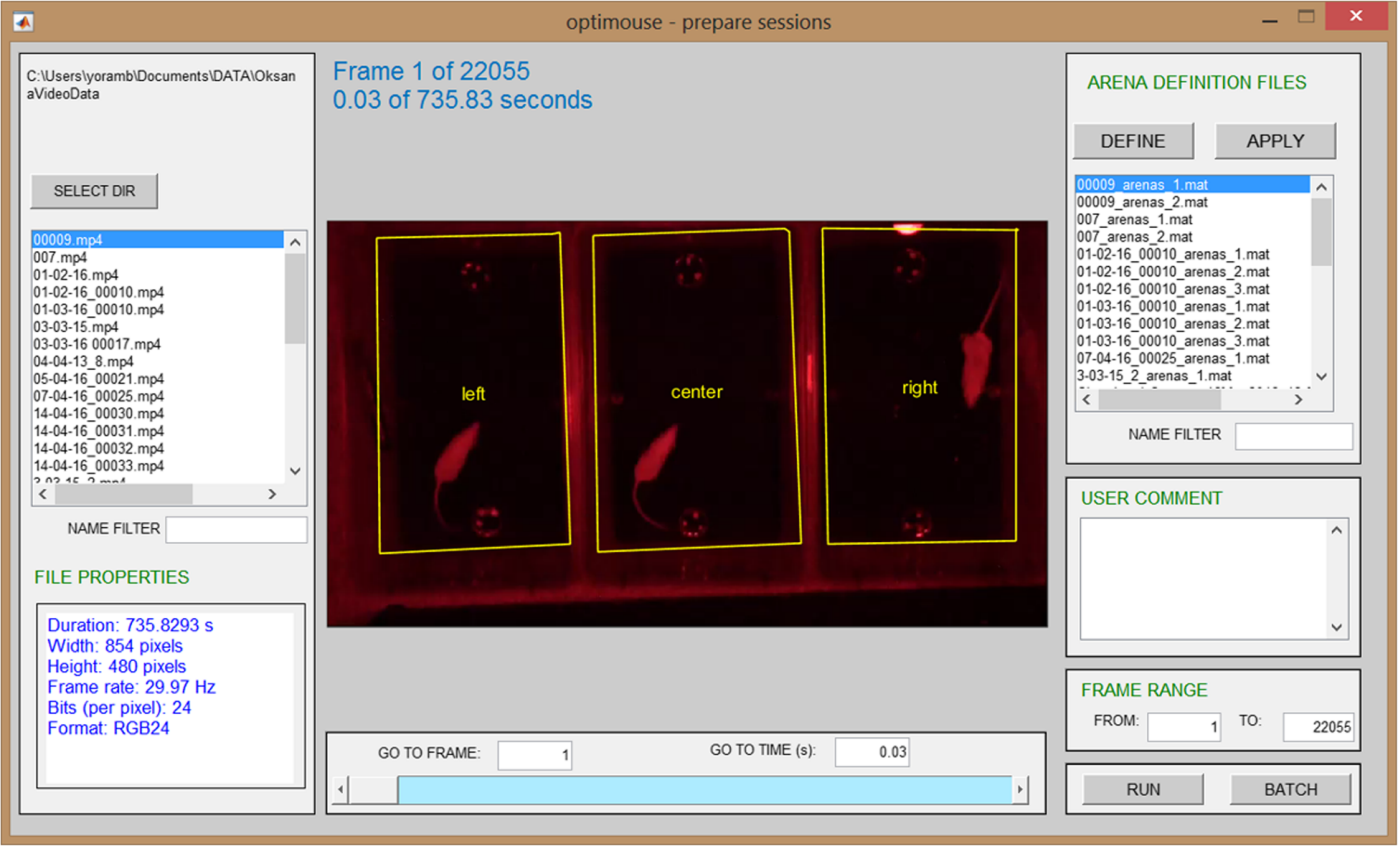
The Prepare GUI. The Prepare GUI is shown after definition of three arenas (named *left*, *center*, and *right*). The GUI for arena definition is accessed via the Define button (see the manual for details)

The first step in analysis is the definition of the spatial and temporal regions of interest in the video file (Fig. 2). See Table 2 for a description of valid video file formats. The Prepare GUI is designed to apply these definitions and prepare the data for subsequent analysis (Fig. 3). Size calibration (i.e., defining the pixel to mm ratio) and arena boundary definitions are implemented using a different user interface that is called from the Prepare GUI (not show here, see Additional file 1). Figure 3 shows the Prepare GUI after three arenas were named and defined. Once the spatial and temporal ranges are defined, processing can be initiated using the *run* button. The result of processing is one or more *sessions*, each including one arena within a video (Fig. 2). During preparation, video frames are converted to greyscale images. Because preparation can be time consuming, it is possible to run preparation for several arenas as a single batch process (Additional file 1).

### Detection of body and nose positions

**Fig. 4.**
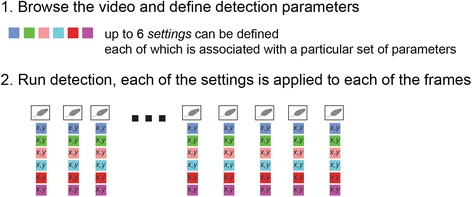
Schematic of the detection stage. In very broad terms, one or more detection settings (up to six) are applied to each of the frames of the video. Each setting involves several user defined parameters and potentially also user specified algorithms. The selection among the various settings is applied in the review stage

**Fig. 5.**
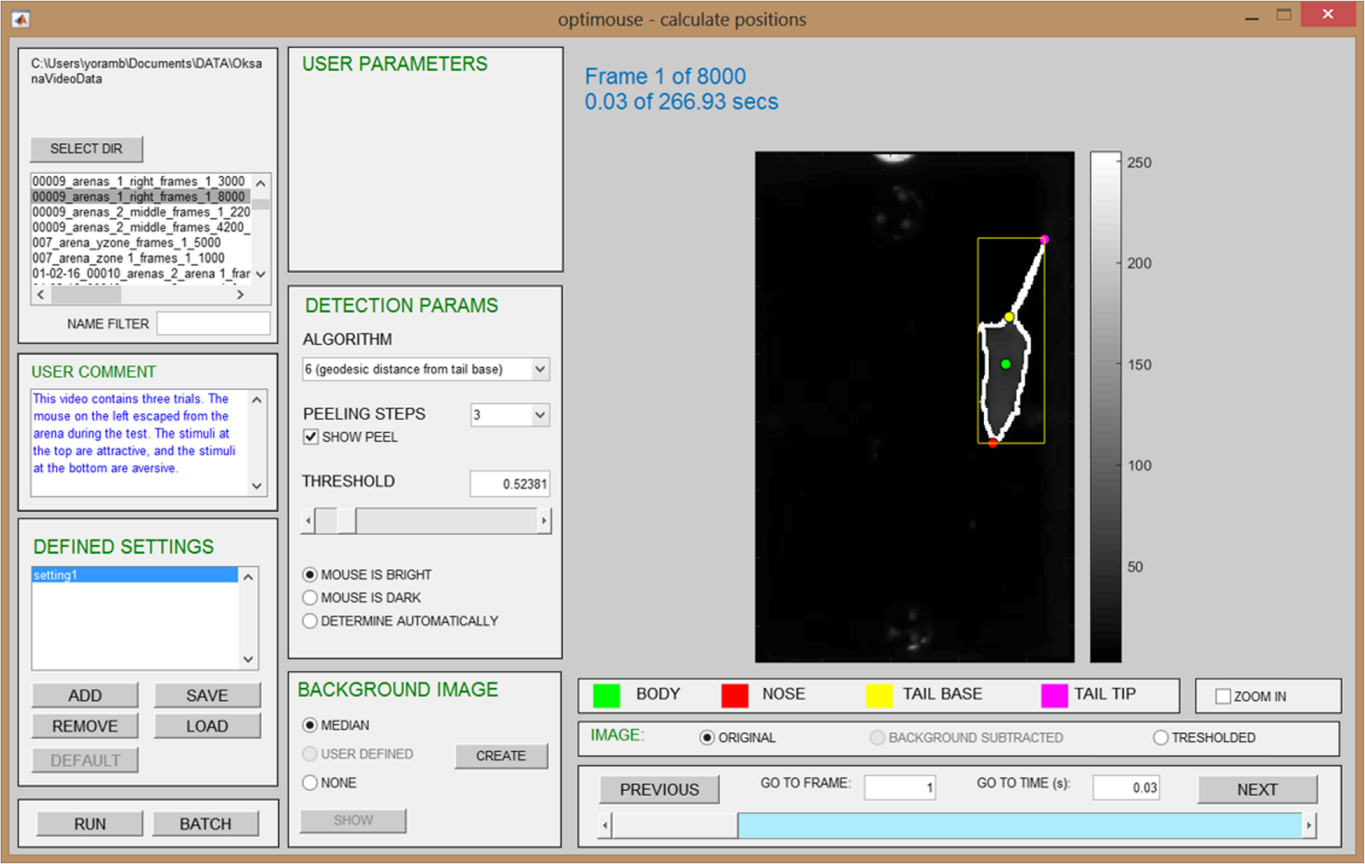
The Detect GUI. The Detect GUI is shown with one setting defined

During detection, the coordinates of the mouse’s body and nose are determined for each frame in the session (Fig. 4). Controls on the Detect GUI allow browsing the video and setting optimal parameters for detection (Fig. 5). The challenge is to correctly identify nose positions as identification of the body center is considerably simpler. Below, we provide a conceptual description of the detection process. Detailed technical accounts of each of the algorithms are given in the user manual (Additional file 1, Section 4.6 and Appendix 10).

**Fig. 6.**
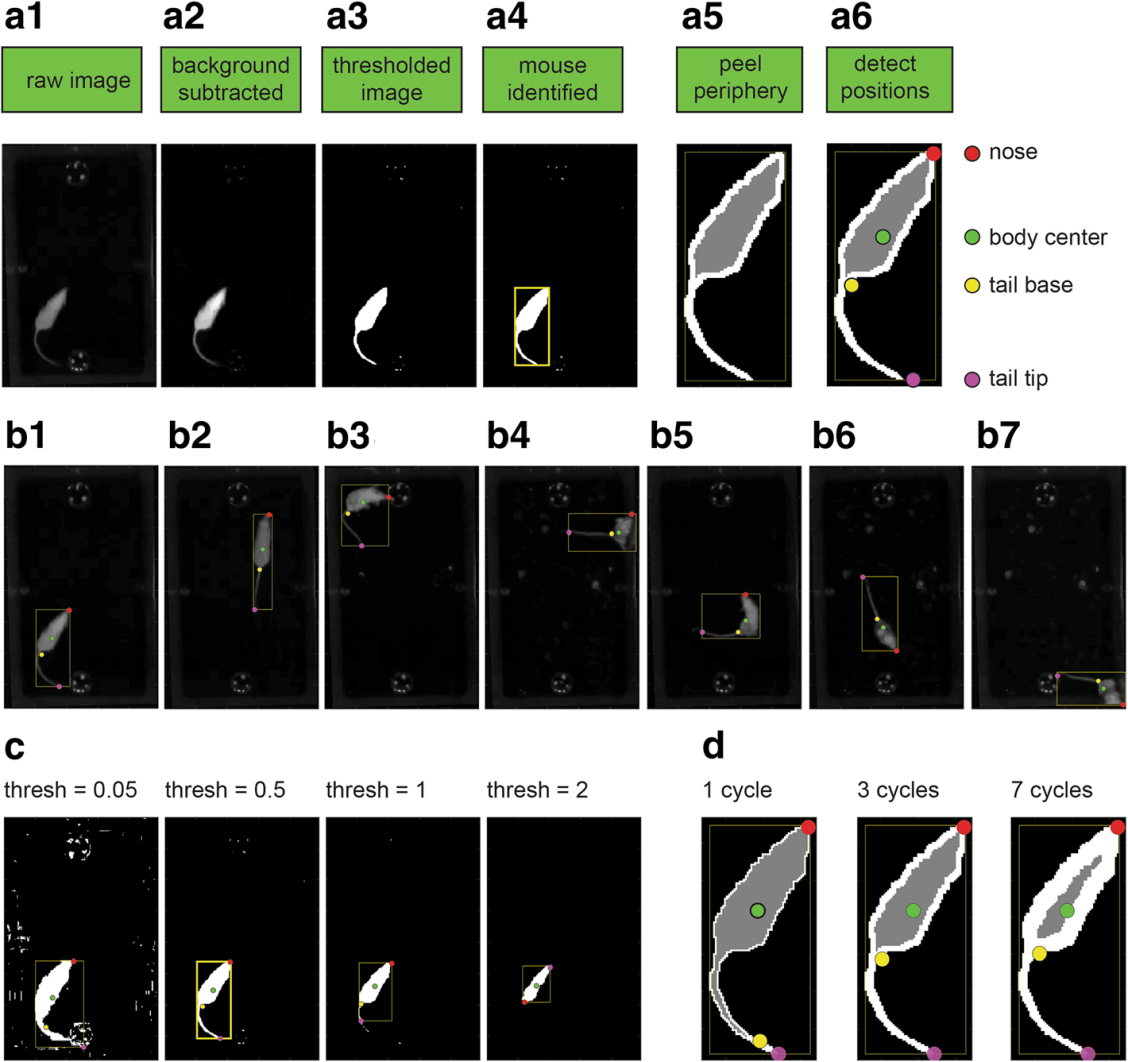
The detection process. **a** The key stages of nose and body detection. **b** Examples of detection of various frames in a single session. **c** Effects of changing the detection threshold. **d** Effects of changing the number of peeling cycles

The key stages of the detection algorithm are illustrated in Fig. 6. Inputs from the preparation stage are greyscale arena images (Fig. 6a1). By default, the median image is subtracted from each frame, resulting in the elimination of static arena features. However, if arena objects move during the session, or alternatively, if the mouse does not, background subtraction can actually confound detection. It is thus possible to either forego background subtraction, or to define a custom background image (Additional file 1). Like the original image, the background subtracted image is a grayscale image (Fig. 6a2). Next, a threshold is set, converting each frame to a binary (black and white) image (Fig. 6a3). The *mouse* is defined as the largest group of continuous “white” pixels in the binary image. Its boundaries are indicated by the yellow rectangle (Fig. 6a4). Note that, in the binary image, the mouse should appear brighter than the background, even if it is darker in reality.

Once detected, the mouse body center is simply defined as its centroid. Detection of the nose is generally more challenging, and is addressed by several features in OptiMouse. The first of these is the availability of several algorithms for detection. Most algorithms rely on detection of the tail, which is identified by a process denoted here as *peeling*, in which the outer layer of mouse pixels is sequentially removed (Fig. 6a5). Since it is the thinnest part of the mouse, the tail is the first part to disappear in this process.

Using the default algorithm, the nose is defined as the furthest point from the tail base, with distance measured along the outer layer of the mouse. Detection using these default settings is shown in Fig. 6a6, with detected landmarks drawn over the mouse image. Of the four landmarks indicated in Fig. 6a6, only the nose and body center are required for subsequent analysis stages (tail base and tail tip represent intermediate stages in the calculation of nose and body positions and are not used further).

Figure 6b shows the results of detection of various frames from the same session, again using the default settings. In these examples, positions are correctly identified for a variety of postures. Generally, however, settings must be adjusted to achieve good identification. The purpose of the Detect GUI is to identify those settings. Two key parameters are the threshold and the number of peeling steps. The effects of changing these parameters are illustrated in Fig. 6c and d, respectively. For both these parameters, a narrow range of values yields good detection. Other parameters that are determined through the Detect GUI include the algorithm used for calculation and the specific background image.

The GUI allows browsing of video frames throughout the entire session, with the aim of finding parameters that provide good detection across most, if not all, frames. Once a successful combination of parameters is found, it can be defined as a *setting* (in Fig. 5, under the *defined settings* panel, the setting was named *setting 1*). The *run* button in the GUI will apply the setting to find nose and body positions in all session frames. As with the preparation stage, detection is a time consuming process which can be run in batch mode.

### Detection of body and nose positions using multiple settings

**Fig. 7.**
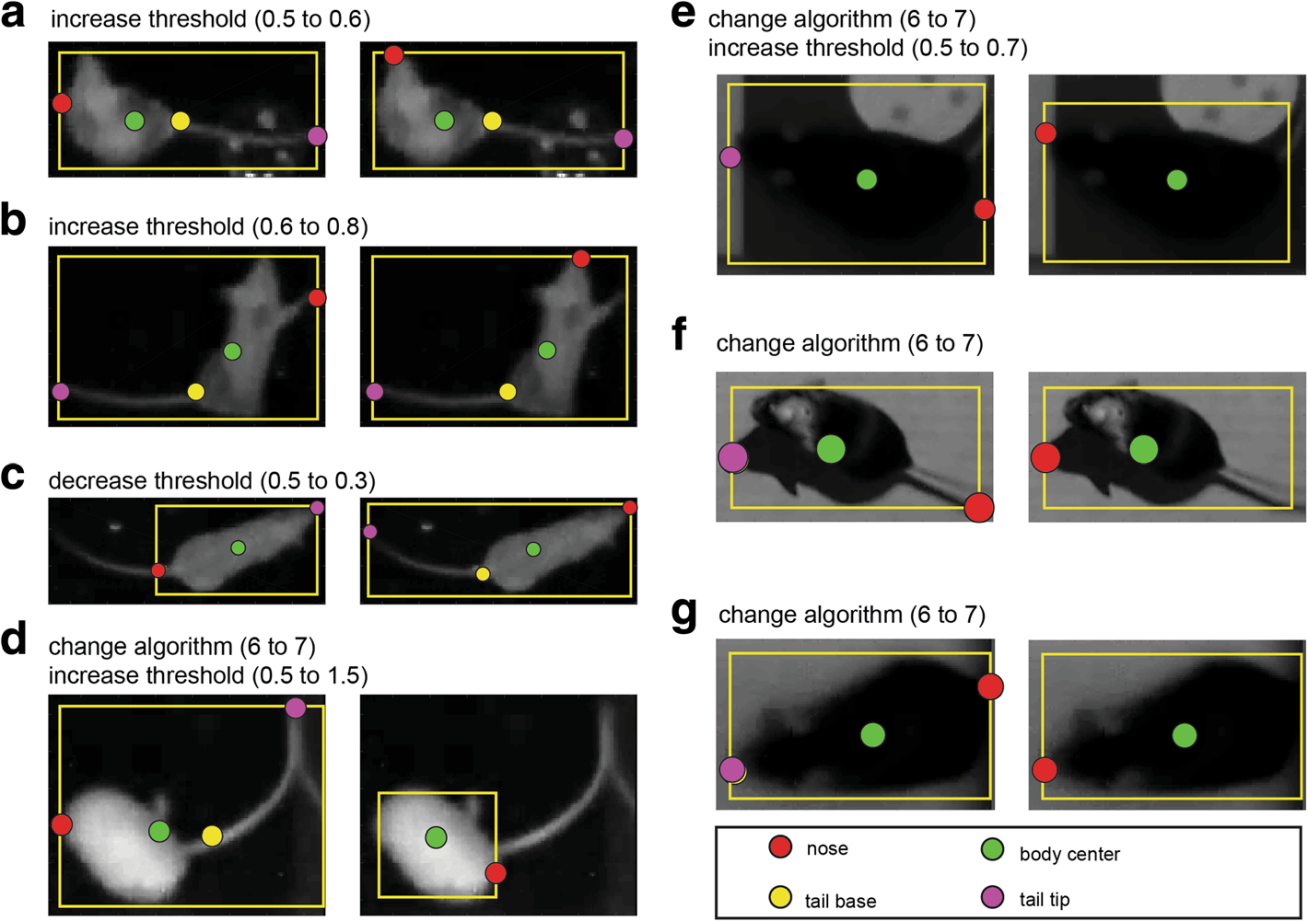
Examples of incorrect detection (left image in each panel) and their correction (right images). Some detection failures can be fixed by adjusting the detection threshold (i.e., **a**–**c**) but others require more extensive adjustments. In (**d**), the mouse is grooming its tail, with the nose positioned close to the tail base. Such cases are difficult to detect consistently in static images, but are apparent when viewed in the context of a movie. Although it is easy to modify the parameters to achieve correct detection in this frame, it is challenging to generate an algorithm that will reliably identify the nose under such cases. In some cases, application of another algorithm is required. For example, algorithm 7 (Additional file 1) is suitable when the tail is not included in the thresholded image. This indeed is the remedy for the examples in (**d**–**g**), sometimes combined with a modified threshold. In (**f**), the left image shows an obvious failure with the tail detected as nose. Detection is improved when the algorithm is changed, yet is still not perfect, since the shadow cast by the nose is detected as the nose. This problem is also beyond the scope of the built-in algorithms, as the shadow is darker than the nose and just as sharp

The images in Fig. 6b highlight the algorithm’s ability to correctly detect positions for a variety of mouse postures. However, generally, one particular setting will not yield correct detection across all frames in a given session. This is illustrated in Fig. 7, showing several examples of detection failures and their correction. To address this, OptiMouse allows the application of multiple detection settings to any given session. Differences between settings may be subtle (i.e., only small change in threshold) or substantial, pertaining to multiple parameters such as the background image, number of peeling steps, and the detection algorithm.

In addition, OptiMouse facilitates the integration of custom functions into the GUI. Any MATLAB function that accepts as input a frame image and returns as output nose and body coordinates may be integrated into the GUI. User defined functions can accept all built-in parameters as well as additional unique GUI inputs. Details on the requirements and application of user defined functions are provided in the manual (Additional file 1).

In summary, the goal of the detection stage is to identify a minimal set of settings, such that, in each frame, at least one yields correct detection. When detection is run with multiple settings, each will be applied to each of the frames (Fig. 4). The batch option applies to multiple settings as it does for single settings.

### Reviewing position detection

**Fig. 8.**
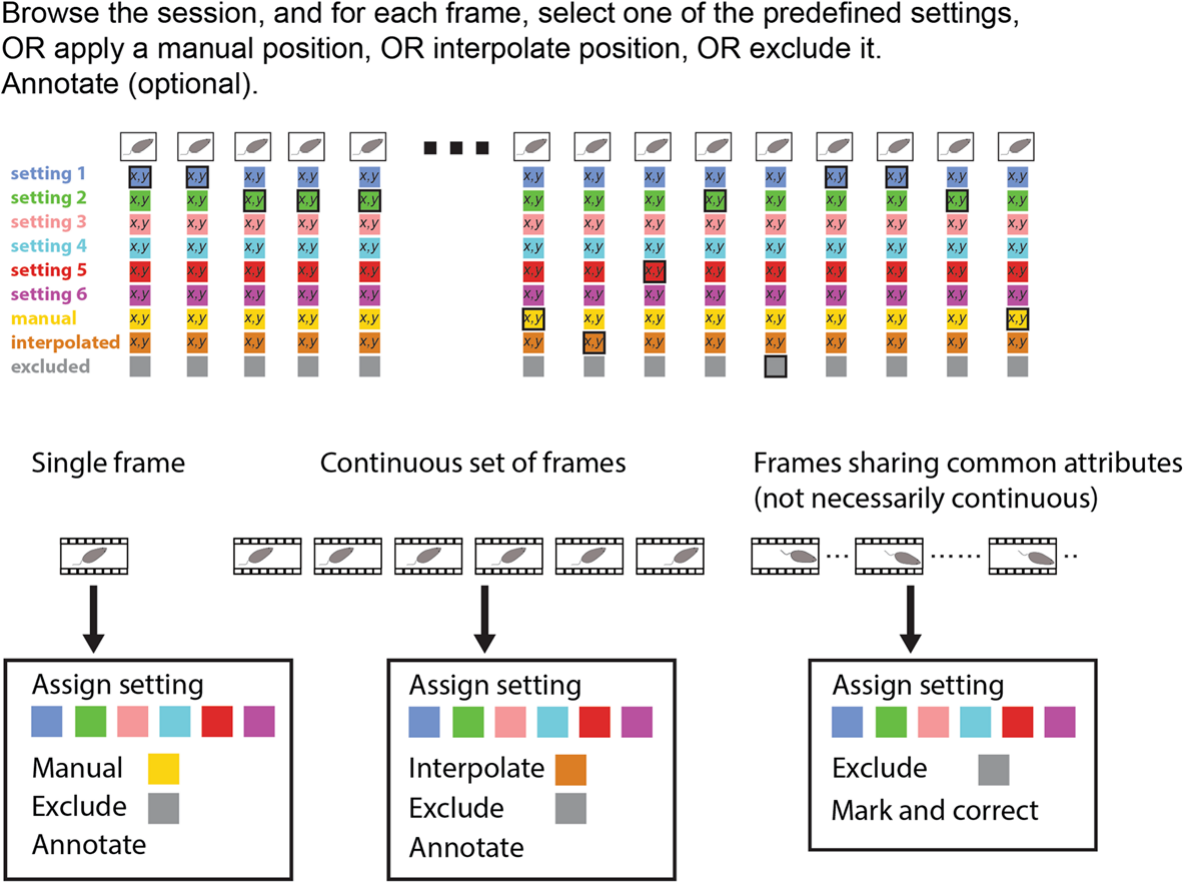
A schematic overview of the reviewing stage. This graphic on top illustrates the operations that can be applied to each frame. The bottom panels show that such operations can be applied to individual frames, to a continuous segment of frames, and to frames sharing common attributes

**Fig. 9.**
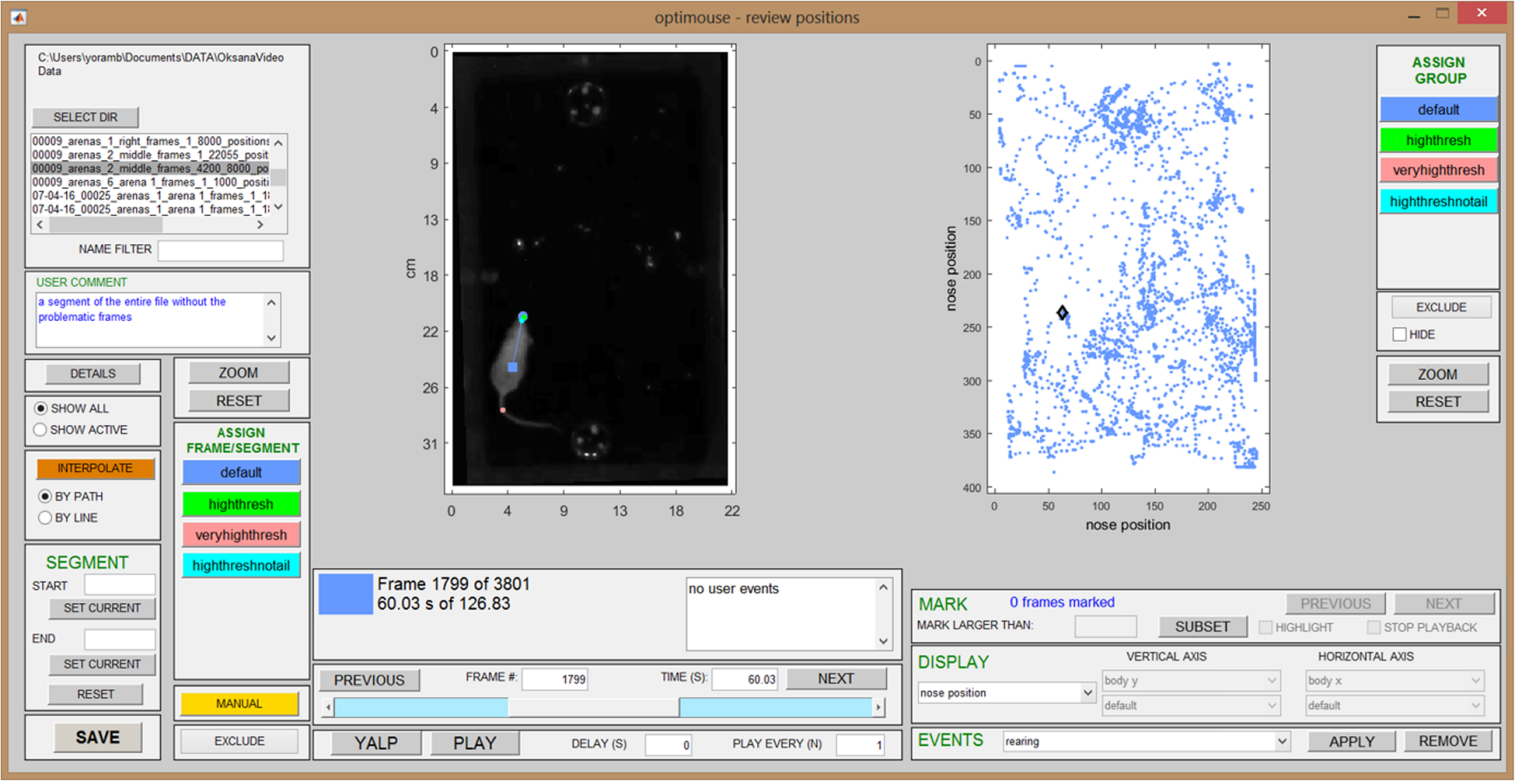
The review GUI. The review GUI is shown after four settings have been defined during the detection stage

The end goal of the reviewing stage is to ensure that positions are detected correctly in all frames. This is achieved by selecting which predefined setting to apply to each frame, and when all predefined settings fail, by assigning user defined settings. Figure 8 shows a schematic of the reviewing stage. Figure 9 shows the Review GUI. Several controls on the GUI allow navigation. These include continuous forward and reverse playback as well as single frame, 1 second, or 1 minute steps, as well as direct access to frames by number or time. Like many other GUI actions, navigation can be implemented with keyboard shortcuts (Additional file 1).

**Fig. 10.**
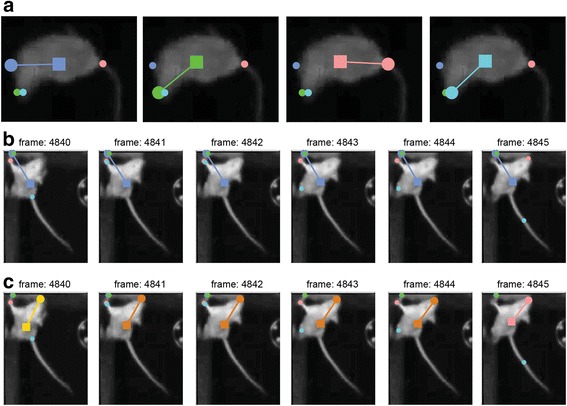
Examples illustrating the application of detection settings. **a** Application of different (predefined) settings to a single frame. The active setting is indicated by a larger circle denoting the nose position, a square denoting the body center, and a line connecting them. In the leftmost frame, the active setting is the default (first) setting. In each of the other frames, there is a different active setting. **b** A sequence of frames with incorrect detection. In this example, the default method fails for the entire segment of frames. **c** The solution involves three stages. First, a manual position, indicated in yellow, is defined for the first frame in the sequence (4840). Next, setting 3 (pink), is applied to the last frame in the sequence (4845). Finally, the set of frames is defined as a segment (Additional file 1), and the positions within it are interpolated. The interpolated positions are shown in ochre (frames 4841-4844). See the manual for a detailed description of the two available interpolation algorithms

Figure 8 also shows the main actions that can be performed in the reviewing stage. The first group of actions involves application of settings to single frames. As seen in Fig. 9 and Fig. 10, in each frame, the positions determined by each of the settings are indicated. Every setting is associated with a different color. Within each frame, there exists one active setting that determines the position eventually assigned to that frame. Initially, all frames are assigned with the first setting as defined in the detection stage. The active setting in each frame is highlighted (Fig. 10a).

The left side of the Review GUI includes buttons that apply each of the settings to the currently shown frame. The images in Fig. 10a show the same frame with each of the available settings applied. In this example, the default (blue) setting does not provide correct detection, but the second (green) and fourth (cyan) settings do. When none of the predefined settings work, it is possible to define manual positions or to exclude individual frames from the analysis.

The second group of actions (Fig. 8) allows application of settings to a continuous sequence of frames. Manual positions cannot be applied to an entire segment in a single operation, but it is possible to assign positions to an entire segment, using interpolation based on the start and end positions (Fig. 10b, c).

### The parameter view

The ability to apply settings to an entire sequence of frames accelerates the correction process, but it nevertheless requires serial viewing of frames. A key feature of OptiMouse, designed to further expedite reviewing, is the option to view information about all frames simultaneously. This is accomplished with the parameter display (right side of the Review GUI, Fig. 9). In this display, each frame is indicated by one dot, with the horizontal and vertical coordinates representing features associated with the frame. The currently shown frame is highlighted by the black diamond. Pressing the left mouse button while the cursor is near a dot will show the associated frame, providing direct access to frames with particular attributes. As described in the manual, there are numerous parameter combinations (views) which can be shown. Here, we provide a brief description of some of these views and the operations that can be achieved with them.

**Fig. 11.**
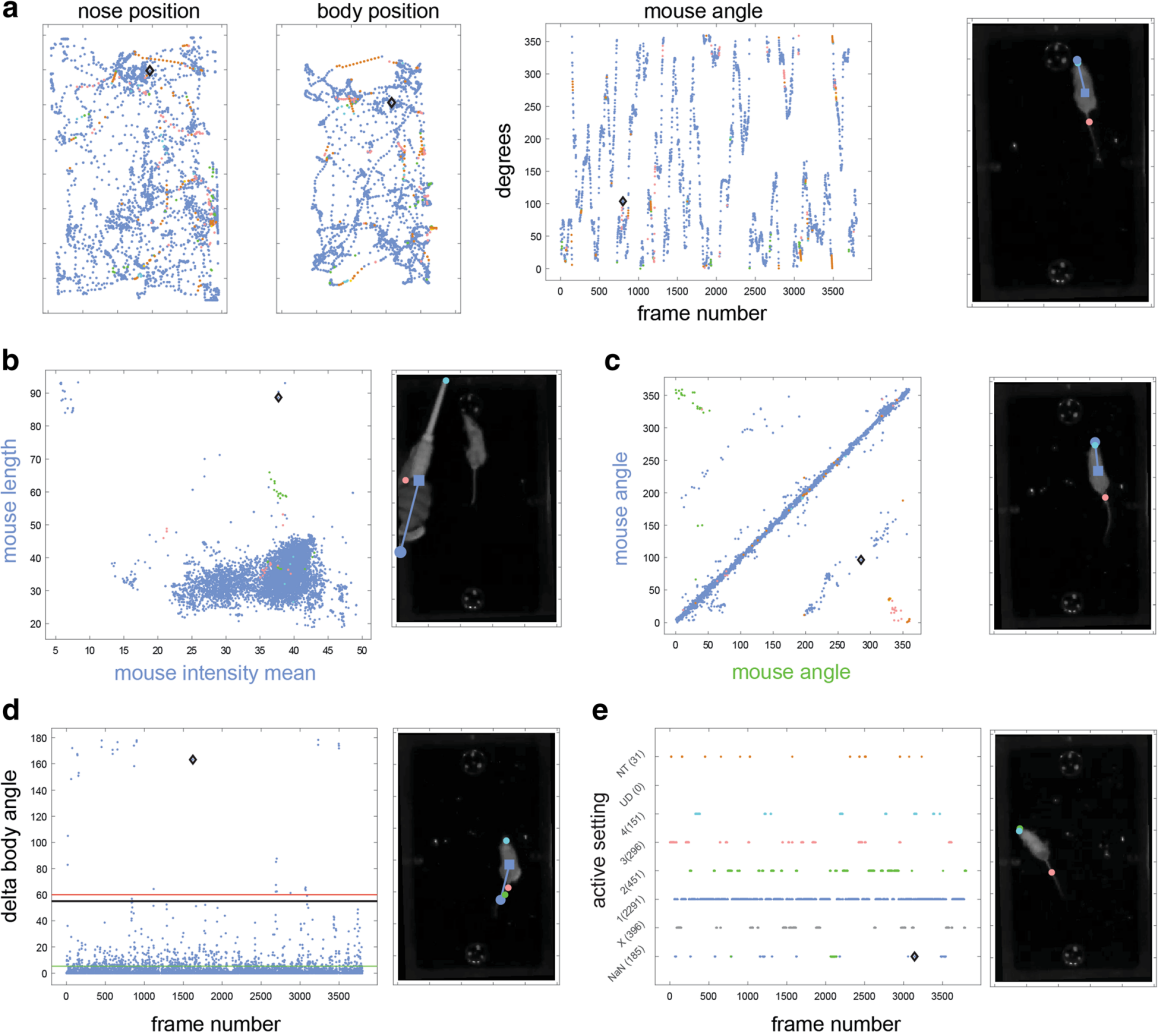
Examples of some parameter views. In all cases, the current frame is indicated by a diamond, and is shown to the right of each view. **a** Views associated with position. **b** Length verus mean intensity of the detected object. **c** Comparison of angles detected by each of the two settings. The settings which apply to each axis are indicated by the label colors (blue and green denoting the first and second settings, respectively). **d** View showing body angle change as a function of frame number. Extreme change values, as shown in this example, often reveal erroneous detections. **e** View showing the setting associated with each frame

Figure 11a shows three views of a session (with 4000 frames). The current frame is shown on the right. In the nose position view, each dot represents the position of the nose, as determined by the active setting for that frame with the dot’s color indicating the active setting. The body position view shows the coordinates of the body center. Thus, the diamond positions indicate the nose and body positions associated with the current frame. The third view in this panel shows mouse angles as a function of frame number.

The parameter views can show many parameters associated with detection in each frame. Examples include mouse length and area, angle, body and nose positions, intensity values of mouse or background, and more. The interface allows plotting of any of these parameters, as determined by any of the settings, against any other parameter as determined by any of the settings. For example, in Fig. 11b, dots represent the length and mean intensity of the mouse. This representation happens to be useful for identifying a frame with extreme feature values, where the detected object is clearly not a mouse.

In Fig. 11c, the dots represent mouse angles as detected by two different methods. Note that axes label texts describe the parameters shown, while their colors indicate the relevant setting. In this example, most dots are located on the diagonal, indicating agreement between the first and second settings. Off-diagonal dots are more likely to represent erroneous detections, since at least one of them must be incorrect. The purpose of views like those in Fig. 11c is to identify frames with discrepancies between settings. Thus, in the particular frame shown (Fig. 11c, right), the first (blue) and the second (green) settings yield angles that are almost opposite. Here, correct detection is provided by settings 1 and 4, while settings 2 and 3 confound tail and nose.

Figure 11d shows another special view where the dots represent the angular velocity of the mouse. Dot positions indicate the angle differences between the preceding and the current frame. Exceedingly high values are associated with erroneous detection in either the frame itself, or in the one before it. This is indeed the case with the frame shown in the right side of Fig. 11d, where the default setting confounds tail with nose. This and two other related views (body speed and nose speed), are useful for detecting, and as described below, for correcting erroneous detections.

Another useful view (Fig. 11e) shows the active setting associated with each frame. In addition to showing the distribution of the different settings, it allows identification of frames that are not associated with any valid position. These are denoted as NaN (not a number) frames. NaN frames lack positions because their active setting does not yield a valid position. This is the case for the default setting in the frame shown in Fig. 11e. In this frame, three settings do yield a valid position, but only two are correct (settings 2 and 4, as can be seen by examining the frame image).

### Operations that can be performed with the parameter view

The importance of the parameter view is its ability to identify frames with particular attributes, as well as to apply changes to a group of frames simultaneously. Some of these operations are described below.

#### Marking frames

**Fig. 12.**
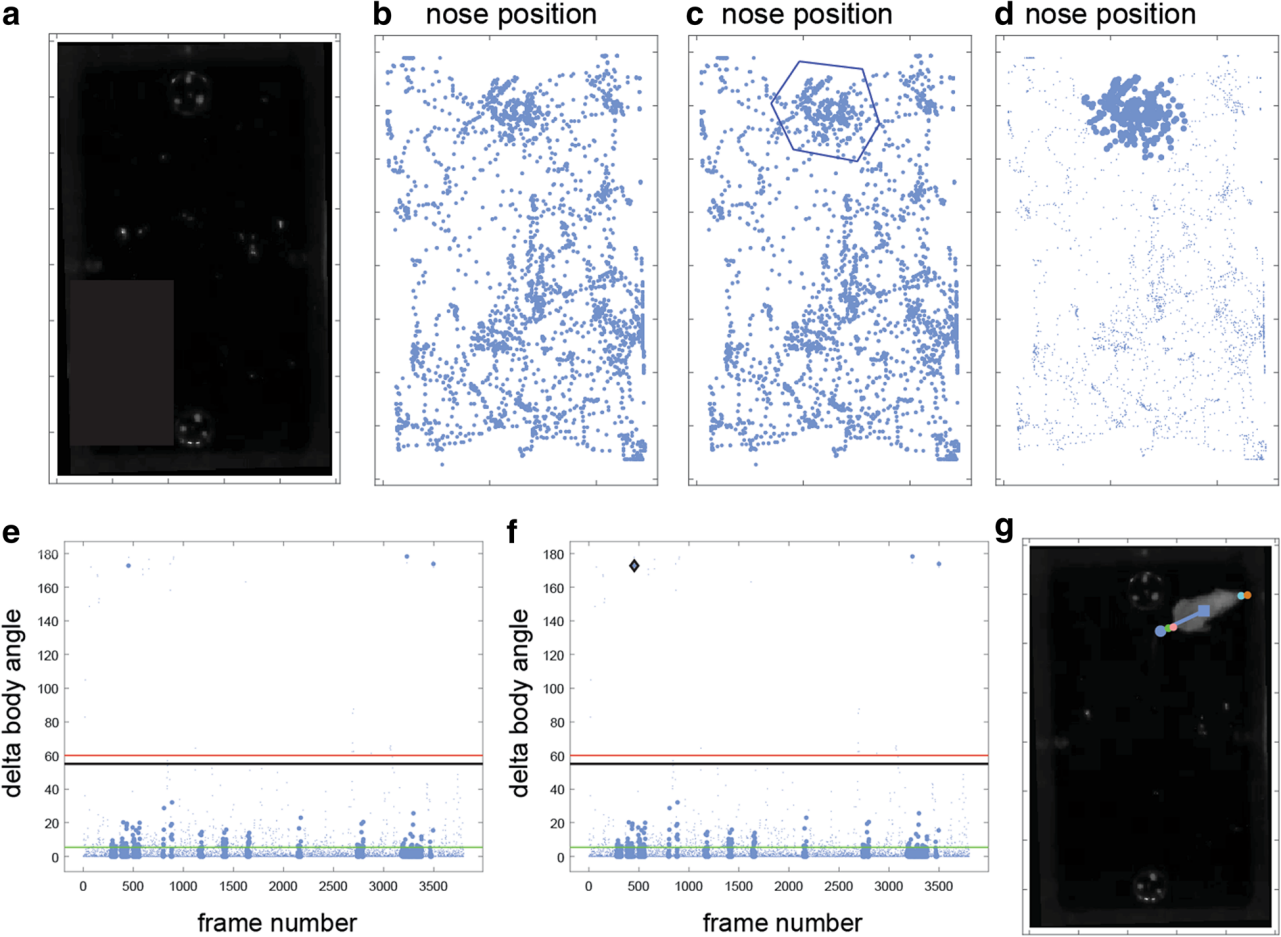
Procedure for marking frames with particular attributes. In this example, frames associated with particular nose positions are marked, and then examined for abrupt changes in direction. **a** One frame showing positions of odor plates in the arena. **b** View showing nose positions before marking. **c** Same view during the process of marking a subset of frames near upper odor plate. **d** In this view, the marked frames are highlighted. **e** After switching to a different view, the marked frames are still highlighted. In the present example, this allows identification of frames that are both associated with particular positions and with high values of body angle changes. **f** Selection of one such dot reveals a frame in which the mouse is near the upper plate and the tail is mistaken for the nose (**g**)

Figure 12b shows the nose position view in an arena containing two odor containing plates (Fig. 12a). If the important measures are time spent investigating the plates, then it may be unnecessary to carefully monitor positions when the mouse is distant from them. To specifically mark those frames in which the mouse is near the upper plate, a polygon can be drawn around the subset of relevant dots (Fig. 12c) using the *subset* button in the *mark* panel (Fig. 9). Once a polygon is defined, the frames are marked. Marked frames are highlighted as bold dots (Fig. 12d). Highlighting is useful for identifying marked frames under different views. For example, Fig. 12e shows marked frames in the angle change view. Highlighted dots associated with high angle change values represent frames that are important and likely associated with detection errors. For example, the dot selected in Fig. 12f corresponds to a frame in which the nose is near the upper plate, but the position is not detected correctly. Focusing on such a subset of frames can significantly expedite the reviewing process by narrowing the number of frames that require reviewing.

Once frames are marked, it is possible to selectively skip from one marked frame to another, or to pause continuous playback on marked frames. Frame marking can be applied in any of the views, providing numerous possibilities to hone in on frames associated with particular features. Examples include frames with particular parameter values (Fig. 11b), discrepant values between two settings for a given parameter (Fig. 11c), frames assigned with particular settings (Fig. 11e), or frames associated with abrupt changes in position (Fig. 11d). The latter are of particular importance to marking, and include body speed, nose speed (defined relative to the body), and the previously described angle change view. In each of these views, frames can be marked not only using polygons (as in Fig. 12c) but also by setting a threshold value, above which all frames will be marked. If the threshold is sufficiently high, then marked frames, or those immediately preceding them, are likely to reveal incorrect detections.

#### Applying settings to a group of frames

**Fig. 13.**
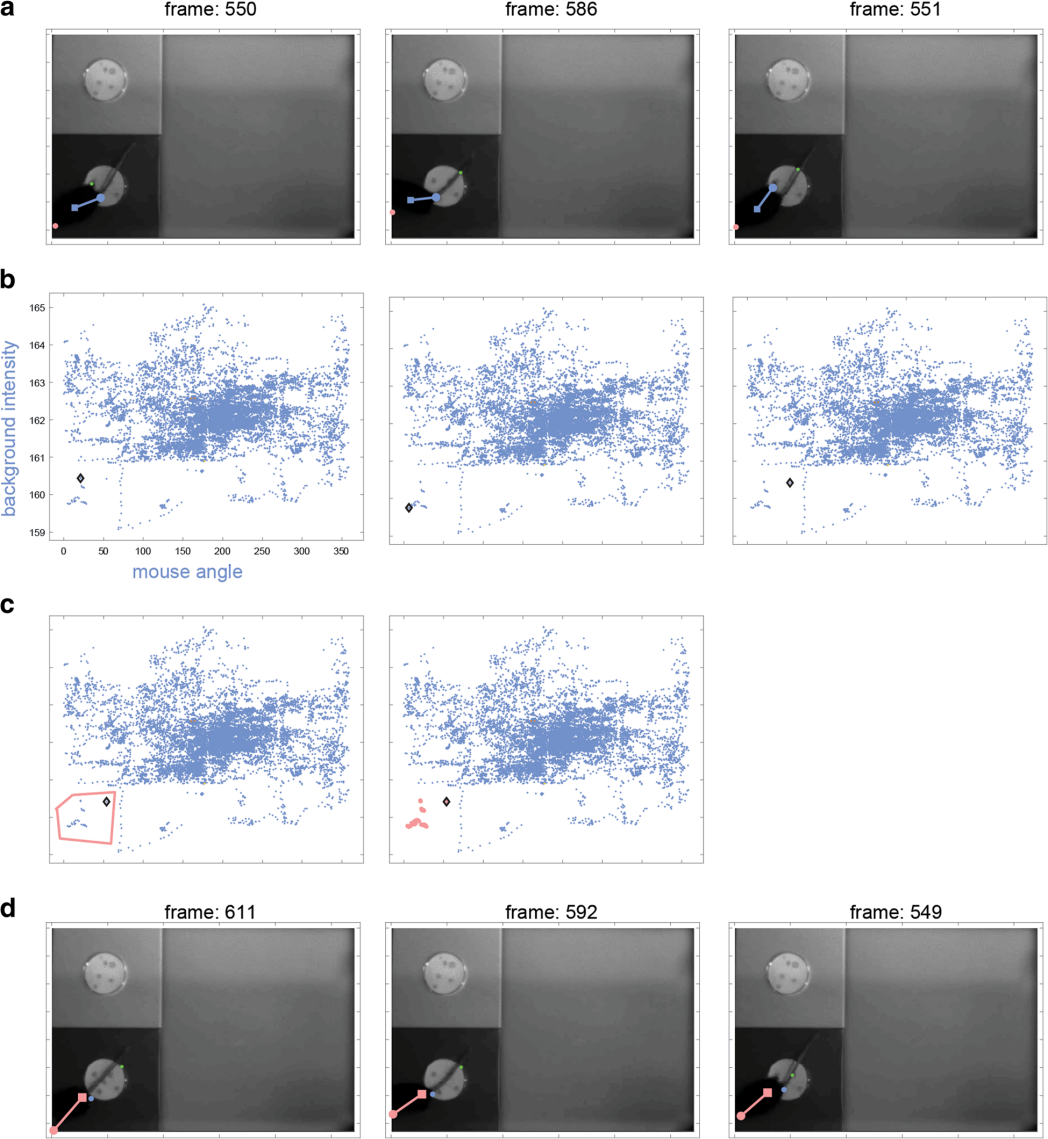
Applying a setting to a set of frames. **a** Three frames that show a similar failure of the first setting cluster together in this view (**b**). Applying a different setting to all these frames using a polygon (**c**), also corrects detection in other frames in the cluster (**d**)

The parameter view can be used to apply a setting to an entire group of frames in a single operation. Technically, the procedure is identical to frame marking. Polygon definitions for specific settings are initiated using the corresponding buttons on the right side of the GUI (Fig. 9). A setting should be applied to a group of frames if it provides good position detection on all, or most, of the frames. To illustrate this, in the three frames shown in Fig. 13a, the default setting fails, but the third setting (pink) correctly detects the nose. In each of these frames, the mouse is located on the dark part of the arena with its head pointing to the left. This property is captured by a combination of the angle and the background intensity, which are the axes represented in the view in Fig. 13b. The clustering of the dots representing these frames suggest that other adjacent frames will also be better detected using the third setting. To apply this setting, the corresponding button is used to draw a polygon around this small cluster of frames (Fig. 13c). Examination of other frames in the cluster (Fig. 13d) validates the assumption that this setting is adequate for them as well. Similar solutions can be applied in other situations where non-consecutive frames sharing some common features are best assigned with a particular setting.

In addition to marking and applying predefined settings, polygon definitions in the parameter view can be used to exclude frames. Frame exclusion is useful for when frames should be ignored due to technical problems with them.

#### Correcting brief detection failures

**Fig. 14.**
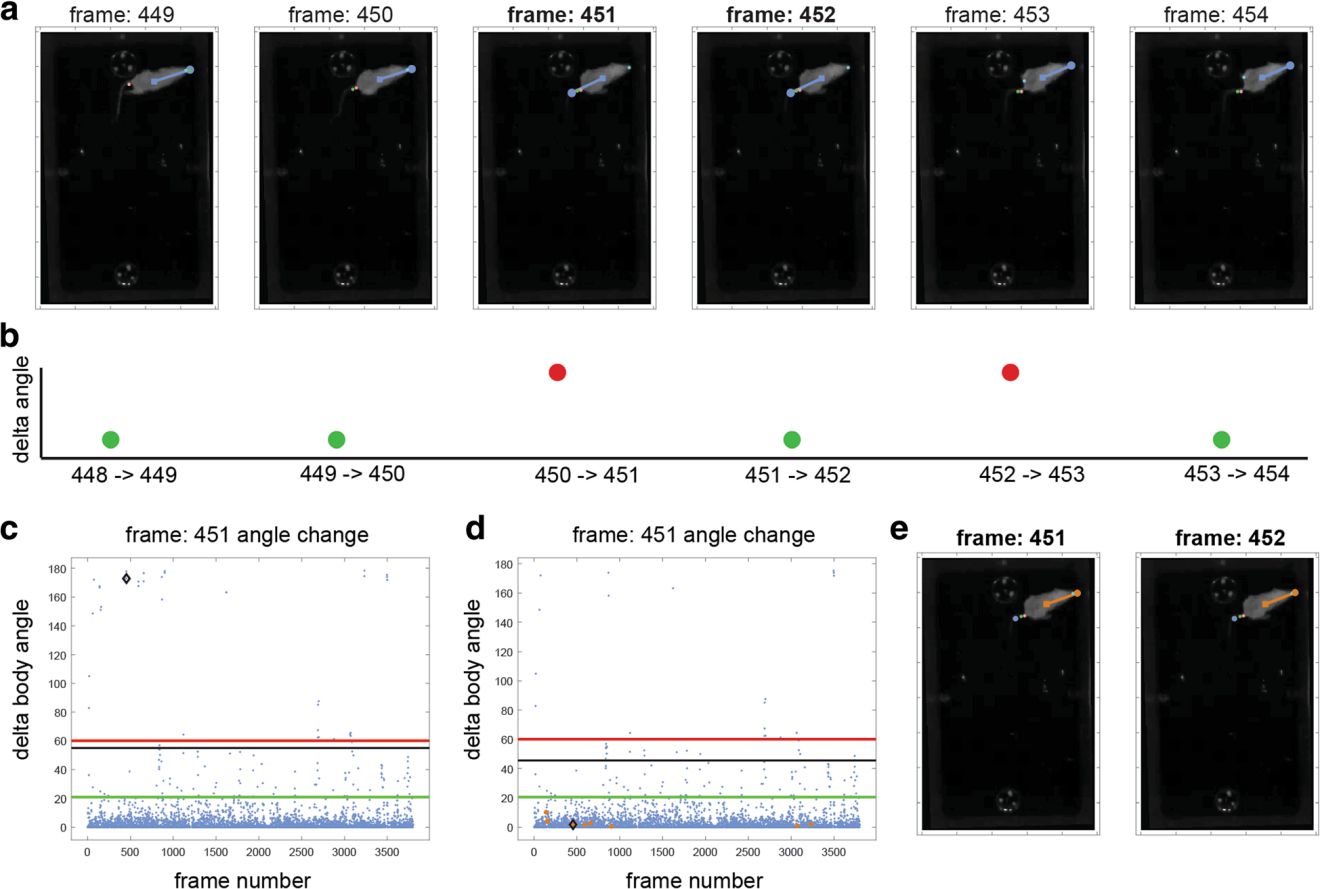
Automatic correction of position detection errors. **a** Sequence of frames with a transient detection failure. **b** Schematic of angle changes in the sequence of frames; the magnitude of angle changes is shown qualitatively. **c** Actual angle changes before correction. **d** Angle changes after correction. **e** Interpolated positions in the two frames that were initially associated with errors

A common type of failure is to confound nose and tail positions. Even when a setting works well for most frames, there may be brief episodes of failures. Such cases will be associated with abrupt changes in the calculated mouse angle. An example is shown in the sequence of six frames in Fig. 14a. Here, the default setting fails for two of the frames (451 and 452), but correctly identifies the flanking frames. Consequently, there are abrupt angle changes between the second and third, and fourth and fifth frames (Fig. 14b). The sequence of abrupt changes can be utilized to detect such transient detection failures, after which positions in the intervening frames can be corrected using interpolation. Figures 14c and d show the angle change view before and after applying correction, respectively. The green and red lines in these panels correspond to user determined parameters that specify how to detect such abrupt transient changes (Additional file 1). Note that after applying correction, the angle change associated with frame 451 (the current frame in Fig. 14c, d) decreases dramatically. Interpolated frames are shown in Fig. 14d as ochre dots. Figure 14e shows the two problematic frames after interpolation. As described in the manual (Additional file 1), this correction is useful only when errors occur rarely, over a background of correct detections.

#### Annotation

An additional, optional, function of the reviewing stage is to assign events to frames. Annotated events can include any user defined feature such as grooming, freezing, rearing, or any other postural or dynamic property. As indicated in Fig. 8, event annotation can be applied to single frames or to continuous segments of frames. See the manual for a more detailed explanation of annotation.

### Analysis

**Fig. 15.**
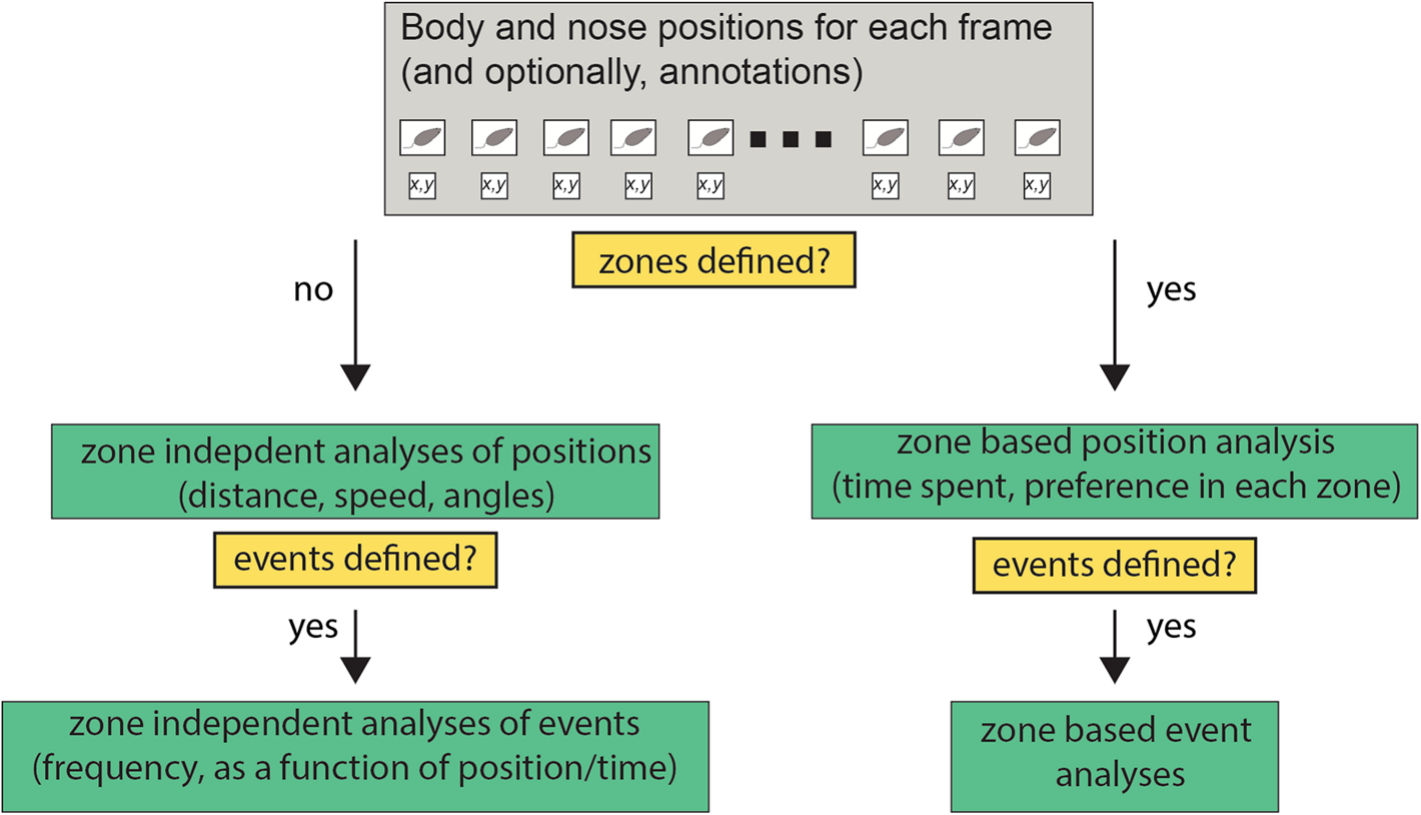
Schematic of possible types of analysis. Flow chart provides a very general description of possible analyses. Results can be shown as figures, saved as MATLAB data files, and for some analyses, displayed on the command prompt

**Fig. 16.**
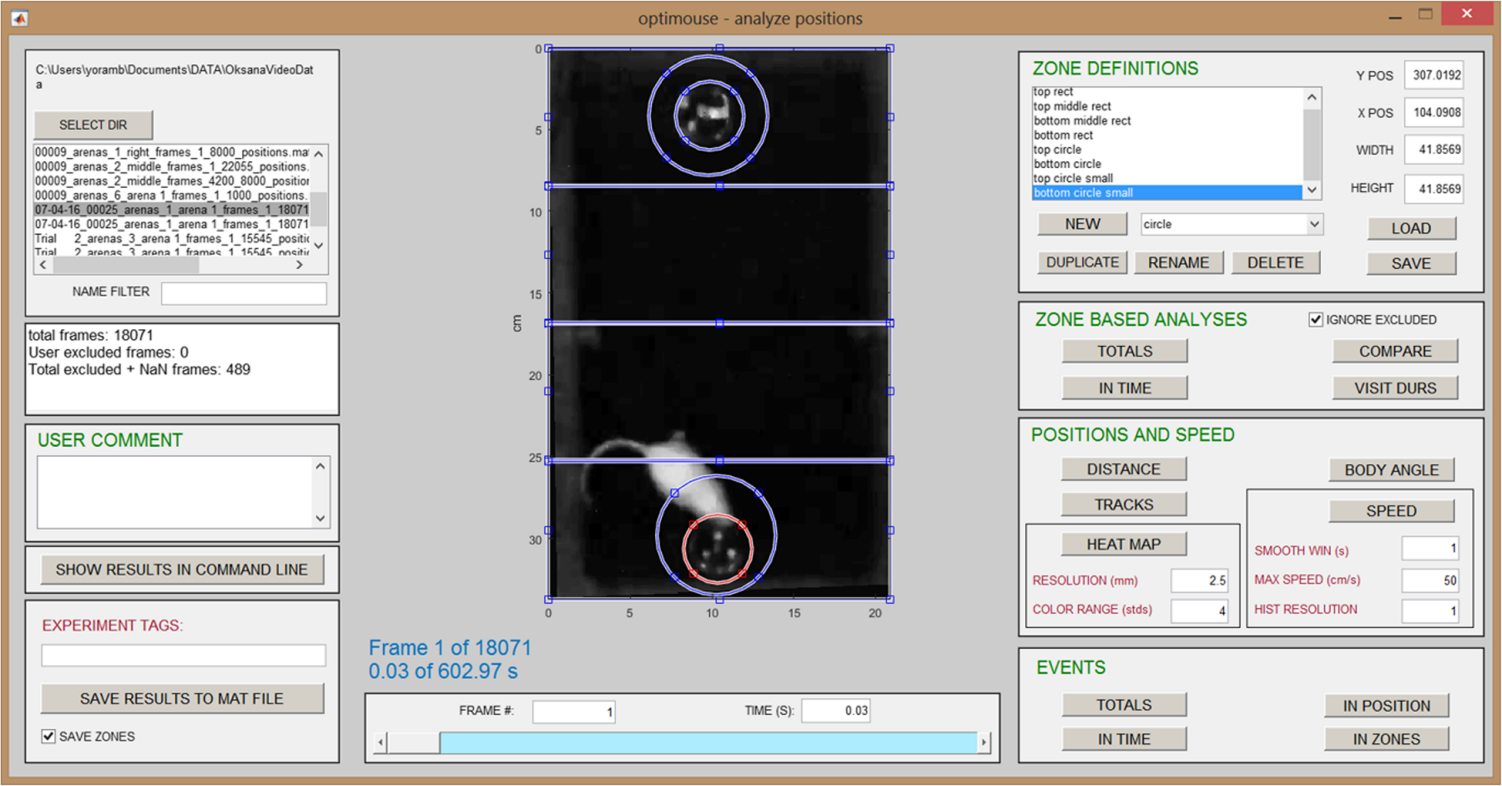
The Analysis GUI after the eight zones were defined

A schematic description of built-in analyses is shown in Fig. 15. The analysis GUI is shown in Fig. 16. Most GUI buttons create graphical displays of particular analyses. In addition to graphical displays, all analyses can be saved into Results files for further processing and analysis. The Results file is a MATLAB data file (*.mat), which contains a single data structure with various fields. The information saved to file is considerably more comprehensive than that shown in the graphical displays, and allows further advanced (custom) analyses. The manual (Additional file 1) describes the Results file format in detail. Some of the results can also be displayed on the command line as text.

Analyses can be divided into several categories. The *positions and speed* panel includes analyses that can be performed independently of any zone definitions (Fig. 16), including tracks made in the arena, position heatmaps, distance travelled, velocity profiles, and angle histograms. Tracks and positional heatmaps are shown separately for nose and body positions.

**Fig. 17.**
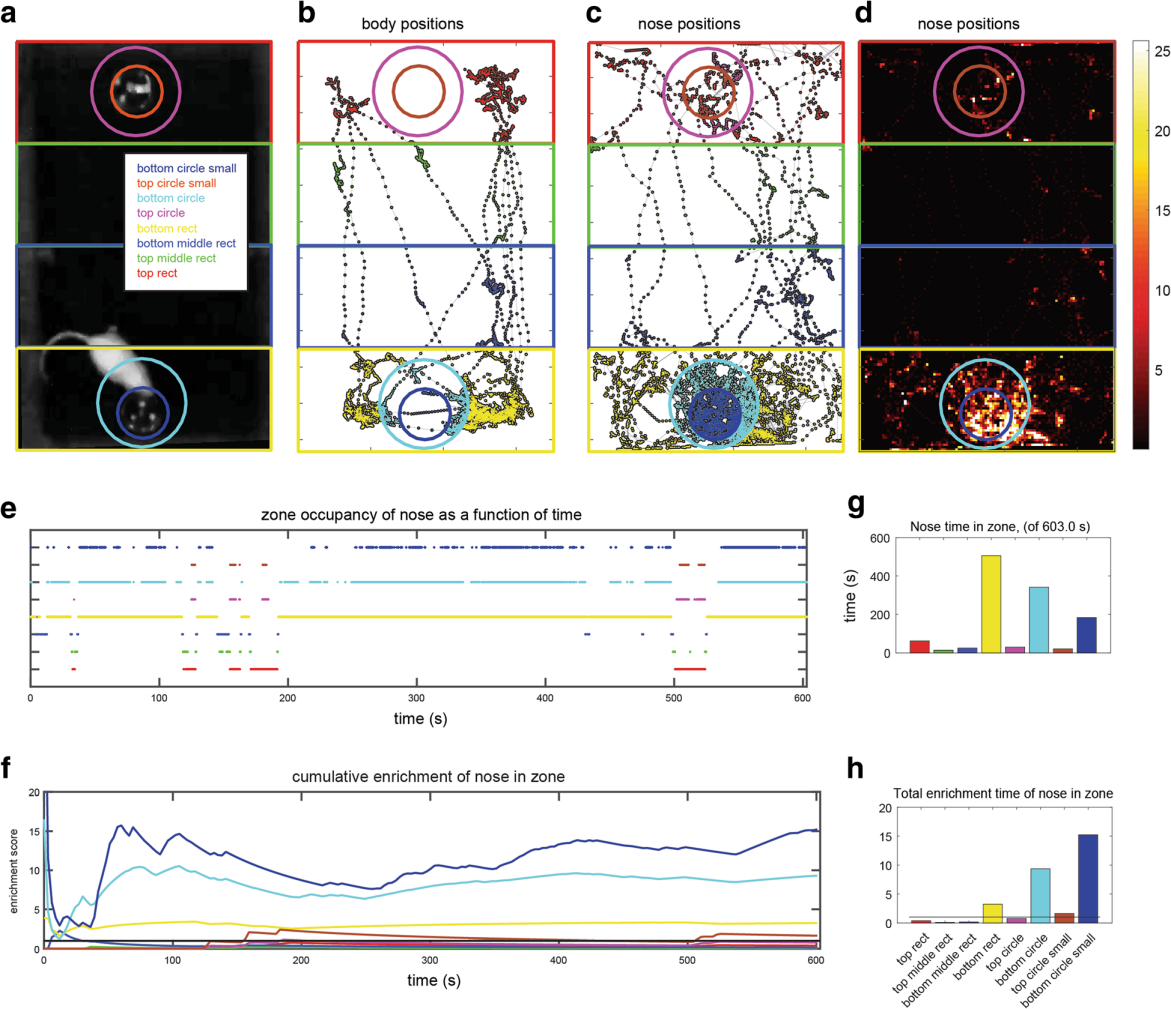
Examples of some graphical analyses. **a** The arena with eight zones defined (same zones shown in Fig. 16). **b** Tracks defined by the body center. Colored zones indicate zone entries. Dots representing positions assume colors of corresponding zones. **c** Tracks made by the nose. **d** Heatmap of zone positions. **e** Zone occupancy as a function of time. Each row corresponds to one of the zones. When the nose is inside a specific zone at a particular frame, this is indicated by a dot. Dots in this display are so dense that they appear as lines. **f** Enrichment score (of the nose) as a function of time. **g** Total nose time in each zone. **h** Enrichment score at the end of the session

Zones of any shape can be defined using the *zone definitions* panel (Additional file 1). Once present, it is possible to analyze how positions relate to these zones. The *totals* button in the *zone based analyses* panel shows the total time spent in each zone (by both body and nose), as well as the enrichment score. The enrichment score is the actual time spent in a zone divided by the expected time, which in turn is derived from the zone’s area, the session duration, and the assumption that all arena locations are visited with equal probability. Thus, the enrichment score reflects zone preference independently of area. Other analyses show how zone visits and the enrichment score evolve during the session, preference among different zones, and the statistics of zone-visit durations. When defined, zones are also shown in the tracks and heatmap displays (Fig. 17b–d).

The *events panel* provides analyses of the distribution of events in time and space. When both events and zones are defined, the occurrence of events within particular zones can be displayed as well (using the *in zones* button in the *events* panel). The complete list of analyses and displays is described in detail in the manual (Additional file 1).

## Results

In this section, we briefly describe a few examples that provide some general insights on analysis of position data using OptiMouse. This analysis is based on a two-odor preference test in a rectangular arena, but these principles apply to a broad range of arena configurations.

Figure 17a shows a movie frame (as seen in the Analysis GUI) following the definition of eight zones. In this session, the mouse was an adult female ICR mouse, which sampled two plates with male urine stimuli. The arena was a plastic cage with dimensions 36 × 20 × 18 cm (L × W × H). Lighting was supplied by four red lamps (General Electric, 25 W, LED light bulbs) placed above the cages and positioned to minimize shadows. The video was made with a HD camcorder (Canon Legria HFR106), mounted 210 cm above the cage floor. The video format was a 24 bit per pixel, RGB, with a 1280 × 720 pixel resolution, taken at 30 frames per second. Movie duration was 602 seconds (18,071 frames). Arena preparation (with a single arena defined, see Figs. 2 and 3) required 745 seconds (on a Lenovo T440s laptop with 12GB of RAM, an i7-4600 processer, and a Samsung SSD 850 EVO disk). Detection (Figs. 4 and 5) used four different settings (setting 1 (default): algorithm 6, three peeling steps, threshold 0.5, setting 2: same as setting 1 with threshold 0.8, setting 3: same as setting 1 with threshold 1.35, setting 4: algorithm 7, three peeling steps, threshold 1.75). Detection required 1407 seconds. Detection using a single setting (the default) required 395 seconds. During reviewing of the 18,071 frames, 489 (2.7%), 262 (1.5%), and 1434 (8%) were assigned to settings 2, 3, and 4, respectively. Eighteen (0.1%) frames were assigned manual positions and 383 (2.1%) were interpolated.

Figure 17b and c are displays of body and nose tracks, respectively, for the entire session. Comparison of the two panels underscores the importance of accurate identification of nose rather than body coordinates. Specifically, it is the nose rather than the body that enters the small circular zones containing odor plates, and provides an indication of preference. Figure 17d shows a heatmap display of nose positions. Figures 17e and f show times of zone visits and the enrichment score as a function of time (only nose positions are shown in these panels). The total time spent in each zone, which is proportional to the number of dots in Fig. 17e, is shown in Fig. 17g. The enrichment score at the end of the session, equivalent to height of the traces in Fig. 17f at the last time point, is shown in Fig. 17h.

Examination of the enrichment score as a function of time for the different zones provides two insights. First, the actual preference values are highly sensitive to the precise definition of the zones. In the current example, the small circular zone at the bottom (blue) is associated with stronger preference than the larger circle at the bottom (cyan). Consequently, the difference in preference between the small lower and upper circles is considerably larger than that for the corresponding large circles. Second, the specific time point at which the enrichment score is considered can have a significant impact on the preference scores. As seen in Fig. 17g, in most zones, the enrichment score changes as a function of time. This in itself is not unexpected, but it is noteworthy that zone preference can change abruptly, and in some cases, may even reverse in time. Thus, care must be taken when defining zones and setting the exact periods for analysis, as these may have a profound, qualitative effect on apparent preferences.

**Fig. 18.**
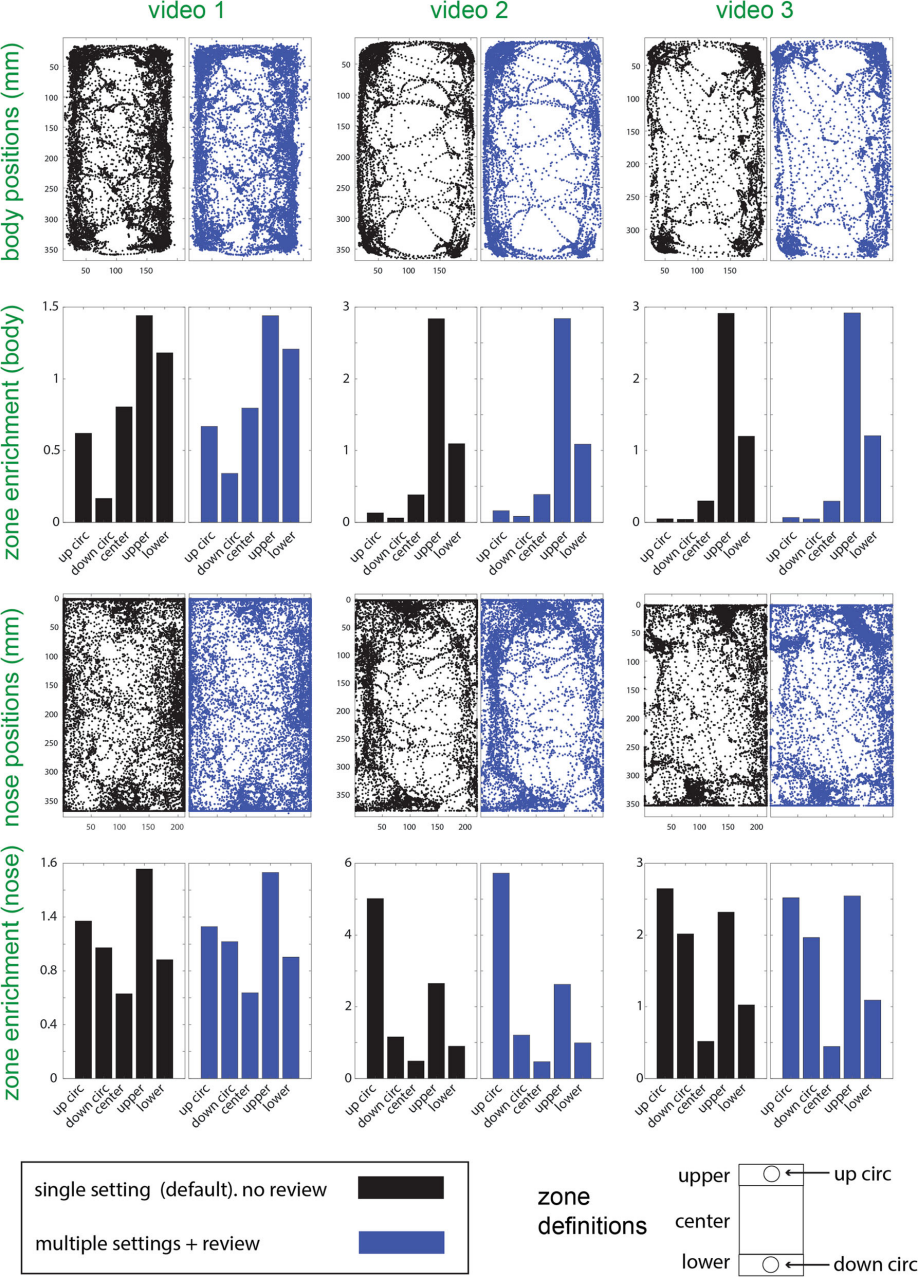
Comparison of positional analysis with and without reviewing for three different videos. Each video is shown in one column, and each row represents one type of analysis. The first row from the top shows body position coordinates. The second row shows enrichment scores of body positions in each of five different zones, whose coordinates relative to the arena are shown at the bottom of the figure. The third and fourth rows from the top are similar to the upper rows, except that *nose*, rather than body positions, are shown. Each panel contains two plots. The plots on the left (in black) show the results using the default setting, while plots on the right (blue) show the same analyses after the application of non-default settings, including manual settings


The analysis shown in Fig. 17 features position data after the reviewing stage. An obvious question is the extent to which the results rely on careful (and potentially time consuming) reviewing. The answer depends on many factors, which involve the video quality, the mouse behavior, and the relevant behavioral metrics, and will thus vary under different situations. In Fig. 18, we address this issue with three different videos (Additional files 2, 3, and 4). All these videos (each 10 minutes long) are similar in attributes to the movie described above and shown in Fig. 17. The videos have been reviewed and the default settings have been overridden when they did not yield correct detections. Thus, the annotated data approximates an error-free account of the mouse positions. The number of frames not associated with the default setting (and the total number of frames) are 671 (18,410), 3621 (18,027), and 3582 (18,071), for videos 1, 2, and 3, respectively. The key point which is immediately apparent from inspection of the position displays and zone preference scores in Fig. 18 is that the annotated and non-annotated data yield very similar results. This comparison indicates that, for these examples, correct detection is achieved in the majority of frames, and that the failures that do occur, do not have a prominent influence on the final results. Thus, the major trends in the data do not depend on the annotation, indicating that the default detection performs well on these videos. Nevertheless, closer inspection does reveal differences, both in positions and in terms of zone preference ratios. We stress that the default setting in OptiMouse will not be appropriate for all movies. However, we do claim that, for many videos, there will be one single detection setting, that even without reviewing, will yield reliable results even in the absence of an elaborate reviewing process. The importance of the reviewing stage is determined by the video and the question asked. For example, if a large open-field arena is divided into four quadrants, the benefits of reviewing are likely to be minimal. On the other hand, if the analysis involves analysis of nose positions on a fine spatial scale, or of body angles, then reviewing is likely to be more crucial. Ultimately, users must evaluate the performance of various algorithms on their own data to determine the importance of reviewing.

Additional file 2: MP4 file containing Video 1 from Fig. 18. (MP4 29118 kb)
Additional file 3: MP4 file containing Video 2 from Fig. 18. (MP4 26693 kb)
Additional file 4: MP4 file containing Video 3 from Fig. 18. (MP4 26098 kb)


OptiMouse does not include a module for population level analysis. However, when the results are saved to a MATLAB file, it is possible to add tags to each session. These tags are useful for identifying sessions for subsequent population level analyses of position data.

## Discussion

In this manuscript, we presented OptiMouse, an open source MATLAB-based code for the semiautomatic analysis of mouse position data. Although most examples above focused on a simple rectangular arena and two-stimulus preference tests, the principles described here apply to other arena configurations and behavioral tests. Because OptiMouse allows definition of arenas and zones or any arbitrary shape, it is suitable for any test that involves a single mouse within a static arena, including tests such as open field behavior, place preference or avoidance, and plus mazes [17]. OptiMouse is not suitable for analyzing social interactions because the detection algorithm is designed for a single mouse in an arena. At the analysis stage, any behavioral parameters that are defined by the body and/or nose positions can be easily extracted from the Results file. This includes measures such as speed, acceleration, and body angle. Freezing episodes can be easily derived from speed velocity profiles. Appendix 9 in the user manual (Additional file 1) includes code for analysis of freezing using the Results file and examples of the analysis. Furthermore, if zones are defined, the dependence of each of those measures on the zone can be easily derived using the Results file which contains occupancy information in each zone for each of the frames. Information about more subtle postural features (such as grooming and reading) can be incorporated into the Results file using manual annotation which allows frame by frame annotation of any user defined event (e.g., freezing, grooming; see the manual). Once defined, the analysis of the occurrence of these events in time and space, and particularly in specific zones, is an integral part of the analysis GUI. Automatic detection of such subtle postural features is beyond the current scope of OptiMouse. Implementing such automatic detection can be achieved via user defined detection functions, which will need to return other variables in addition to the required variables that are returned by default (e.g., body and nose positions). See Additional file 1: Appendix 5 in the manual for a detailed explanation of the outputs of the detection functions. OptiMouse does not support an explicit synchronization signal, but alignment of behavioral data with non-video data (such as electrophysiological data) is possible if the non-video data contains a time stamp that is aligned to the video. Since the Results file contains a variable with the time of each frame and a frame by frame description of all position and movement parameters, events and behavioral features can be compared at the temporal resolution of the video frame rate.

At a basic level, OptiMouse provides a simple yet comprehensive solution for the analysis of position data, starting with video data and culminating with graphical displays. At a more advanced level, OptiMouse is an expandable software with multiple tools designed to achieve high accuracy detection and more advanced analyses of positional data. Using OptiMouse at a basic level is appropriate for videos with good quality, when high accuracy determination of nose positions is not critical, or when the relevant measures concern body rather than nose positions. Under these conditions, after defining arenas, it may suffice to apply one detection setting and directly analyze the data, entirely skipping the reviewing stage. The strength of OptiMouse, however, lies in the ability to achieve high quality nose position determination under less than ideal conditions. This ability involves two elements. First, multiple settings can be defined for any given session (Fig. 4). Second, positions are assigned to each frame based on the settings that yield the most accurate detection (Fig. 8). When none of the predefined settings work, positions can be assigned manually or interpolated.

While setting detection parameters, users have almost full control over all aspects of detection, including background image subtraction, threshold level determination, number of peeling cycles, and selection of the algorithm to apply. Despite this flexibility, and despite the ability to apply multiple settings, the built-in algorithms cannot cover all image detection scenarios. Acknowledging such limitations, OptiMouse is designed to seamlessly integrate user defined functions into the interface as if they were built-in.

Indeed, the simplest approach to expansion of OptiMouse is to apply custom detection algorithms. Custom algorithms may be slight variations of built-in algorithms, but can also implement entirely novel approaches. They may also use other parameters in addition to those integrated within OptiMouse. It should be noted that the algorithms that can be most easily integrated are limited to single frame analysis of grayscale converted images. Incorporation of color information, usage of prior knowledge in any given frame (e.g., using Kalman Filters), and automatic derivation of other positional features (e.g., automatic detection of rearing, grooming [7, 8]) are potential improvements that will require more extensive modification of the code.

When it is important to minimize detection errors, the reviewing stage is crucial. One way to review a session is to watch it in its entirety, pause on errors, and correct those using predefined settings or a manually defined position. If there are many detection failures, then serially viewing and correcting all errors is impractical. The parameter display in the Review GUI was designed to facilitate reviewing, by allowing simultaneous visualization of all frames using an approach adopted from spike sorting software [18]. Namely, in spike sorting software, multidimensional spike shape data is often represented by a small number of principal components. Principal component analysis is not easily applicable to variable movie images, but their dimensionality can be effectively reduced by representing their salient features. The advantage of such a display is that it allows viewing, marking, or changing settings for frames that share common attributes. In particular, the ability to focus on rapid changes and discrepancies between different settings is useful for identifying frames with likely errors. The ability to target on specific arena locations (Fig. 12b–d) allows focusing on those frames where correct detection is critical. Practically, the use of keyboard shortcuts can significantly accelerate the reviewing and correction process.

In summary, OptiMouse is a fully documented MATLAB-based open source program, which users can expand according to their own particular needs. We anticipate that OptiMouse will be very useful to other researchers in the field, providing a simple, accurate, and free solutions for analysis of mouse position data.

## Additional files

Additional file 1: PDF user manual for OptiMouse. (PDF 6406 kb)
