# Supplementary material for: OptiMouse: a comprehensive open source program for reliable detection and analysis of mouse body and nose positions
Source: BMC Biol. 2017 May 15;15:41. doi: 10.1186/s12915-017-0377-3 (PMC5433172; doi:10.1186/s12915-017-0377-3)
Supplement: Supplementary file 4 — PDF user manual for OptiMouse. (PDF 6406 kb) [file 12915_2017_377_MOESM1_ESM.pdf]

OptiMouse User Manual

1. Overview

OptiMouse is a MATLAB program designed for analyzing mouse positions in a behavioral arena. Its OptiMouse reflects the goal of optimizing the accuracy of position detection. OptiMouse is an open-source code, and all analysis stages are transparent and configurable. In particular, OptiMouse allows integration of custom written functions with a built-in set of algorithms.
The key assumption behind OptiMouse is that no single algorithm will achieve accurate detection of nose positions in all sessions, nor on all frames within a session. Therefore, OptiMouse allows application of multiple detection algorithms to any given session, among which the best algorithm is selected for each frame.
OptiMouse requires the main MATLAB code, as well as the statistics and image processing toolboxes. It was developed on MATLAB releases 2015b and 2016a. It is not compatible with some older versions. It was tested on a PC platform. Graphical outputs may vary with different screen resolutions.
Please report errors and suggestions to Yoram Ben-Shaul, yoramb@ekmd.huji.ac.il

1.1. Scope and limitations of OptiMouse

OptiMouse is designed for analyzing positions of one mouse in an arena. It is thus not suitable for analyzing social interactions. Object detection is based on the assumption that the mouse is either darker or brighter than the background. Although a mouse does not have to be uniformly colored, detection of mice with patches of different intensities could compromise reliability of detection. Color information is not used, since video frames are transformed to single channel intensity images. In principle, all formats that are supported by the MATLAB *VideoReader* function should work. Currently tested formats are: *mp4*, *mpg*, and *wmv*. OptiMouse is designed to optimize detection accuracy, not speed. It is not suitable for real time detection.

1.2. Main analysis stages in OptiMouse

Analysis begins with a video file and ends with various measures associated with mouse position within an arena.
In broad terms, the analysis includes four main stages:
1. Defining the spatial limits of the arena and the temporal range of the video for analysis.
2. Setting parameters for position detection, and run detection.
3. Reviewing the outcome of detection, selecting optimal algorithms for each frame, and correcting positions if required. The review stage also allows video annotation.
4. Analysis of position data.

2. Running OptiMouse

2.1. Installation and starting

Before running OptiMouse, the *optimouse* folder and the *optimouse_user_definitions* folders must be added to the MATLAB path. The *optimouse* folder contains the main code files. The *optimouse_user_definitions* folder contains user specific definitions. These two folders should reside under the same parent directory. There are several ways to set the MATLAB path. One is to type *pathtool* on the MATLAB command prompt. Typing *doc pathtool* opens a MATLAB help window explaining the *pathtool* function and providing more information about the concept of the MATLAB path.

Once OptiMouse files are included in the MATLAB path, the program can be started by typing
>> optimouse
on the MATLAB command prompt.

This will open the **OptiMouse GUI**, which provides access to the four main analysis stages (**Figure 2.1**) and to this user manual (**INSTRUCTIONS**).

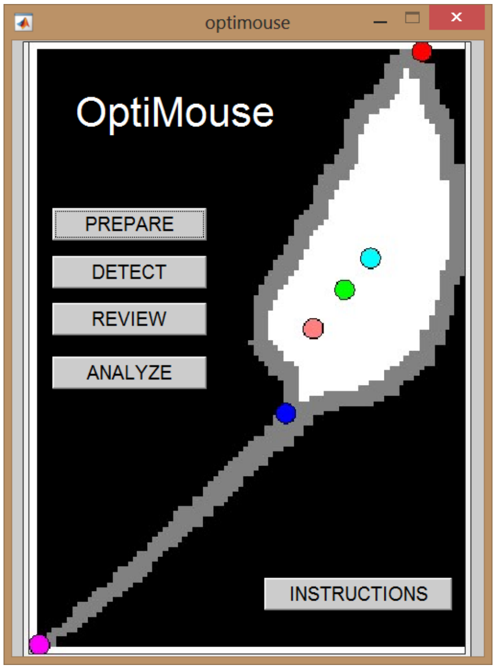

Figure 2.1. The main OptiMouse GUI

2.2. A very quick start guide

This section is intended for users that do not have patience for wordy manuals. The sequence of steps below defines the shortest path from a video file to analyzed results. However, it is only appropriate for those videos for which detection is easy. Most features are entirely ignored in this description.

1. After installation (**Section 2.1**), type *optimouse* on the command line
2. Press the **PREPARE** button in the OptiMouse interface (**Figure 2.1**)
3. Press the **SELECT DIR** button (**Figure 3.1**) to specify the directory containing the video files
4. Press the **DEFINE** button (**Figure 3.1**) to define the arenas
5. Press the **CALIBRATE** button (**Figure 3.2**) to specify the dimensions of the arena
6. Resize the yellow square until its edge matches a known distance on the arena (**Figure 3.3**)
7. Enter that distance (in *mm*!) into the yellow edit box (**Figure 3.3**)
8. Press the **APPLY** button to set the scale (**Figure 3.3**)
   [The dimensions are now calibrated]
9. Press the **NEW** button to define a new arena (a rectangle by default)
10. Drag and resize the rectangle to define the arena for analysis

11. Press the **SAVE & APPLY** button (**Figure 3.5**)
[The arena has been specified and is applied to the current video]

12. Back in the **Prepare GUI** (**Figure 3.7**), set the range of frames for analysis in the **FILE RANGE** panel (by default all frames are included).

13. Press the **RUN** button (**Figure 3.7**) and wait until the progress bar disappears.
[The session has been prepared and positions can now be detected]

14. Press the **DETECT** button in the **OptiMouse GUI** (**Figure 2.1**). Select the file corresponding to the session just created in the listbox on the upper left (there is no need to select the video directory again).

15. In the **DETECT GUI**, examine the result of position detection. Note if body landmarks are correctly identified, if the yellow rectangle bounds the mouse, and if the tail and periphery are seen as white lines (as in **Figure 4.1**).

16. If detection is not satisfactory, modify the parameters. If detection seems completely off, try checking the **MOUSE IS BRIGHT** and **MOUSE IS DARK** radio buttons (perhaps the automatic detection failed). Change the threshold, the peeling stages, and, if the mouse does not have a visible tail, consider using algorithm #7.

17. If none of the settings results in good position detection, it is probably a good idea to read the manual section on the detection (**Section 4**).

18. If detection is satisfactory, browse the movie using the **PREVIOUS** and **NEXT** button, or jump or scroll to various frames in the session, using the controls on lower right side of the **Detect GUI**.

19. If detection is satisfactory on all (or most) frames, press the **ADD** button in the **DEFINED SETTINGS** panel. Name the setting when prompted. This will define the current set of parameters as a *setting* (see **Figure 4.6**).
*In this rapid procedure, we are defining only one setting. If one setting does not address the large majority of frames, multiple settings must be defined. This is described in **Section 4.9**.*

20. Press the **RUN** button in the **Detect GUI** (**Figure 4.6**), and wait until detection is complete (the progress bar will disappear).
[Body and nose positions have now been detected in each frame]
*Following this rapid procedure, we are skipping the reviewing stage. However, if multiple settings were defined in the detection stage, if there are many failures requiring manual settings, or if it is desired to annotate frames with events, then the reviewing stage is required (**Section 5**). Advancing to the analysis stage without reviewing also does not allow evaluating the extent of incorrect detection or their correction.*

21. To show the **Analysis GUI** (**Figure 6.1**), press the **Analyze** button in the **OptiMouse GUI** (**Figure 2.1**).

22. Select the *positions file* created in the detection stage from the upper left listbox. It should be easy to identify the file by the names of the original video and the arena.

23. For general analysis of positions, use the buttons in the **POSITIONS AND SPEED** panel. This will create displays similar to those shown in **Figures 6.2**, **6.3**, **6.4**, **6.5**, and **6.6**.

24. To analyze the preference for particular regions in the arena, zones must be defined. To define a new zone, press the **NEW** button in the **ZONE DEFINITIONS** panel (**Figure 6.7**). The type of zone is specified using the listbox to the right of the **NEW** button. Resize and position the zone over the desired part of the arena. Add as many zones as needed. For a detailed description of zone definition, see **Section 6.3**.

25. Once at least one zone has been defined, the buttons in the **ZONE BASED ANALYSES** panel (**Figure 6.7**) are activated, making it possible to generate displays of zone specific analyses, such as shown in **Figure 6.8**, **6.9**, **6.10**, **6.11**, **6.12**.
26. Press the **SAVE RESULTS TO MAT FILE** button on the analysis interface (**Figure 6.1**) to save the results into a MATLAB file. The *results file* thus generated contains all analysis results. It is described in **Appendix 8**.
27. Now, read the manual.

3. Preparing Sessions

The purpose of the first stage is to define the spatial extent and the temporal range of interest in the video file. To begin press the **PREPARE** button on the **OptiMouse GUI** (**Figure 2.1**). This will open the **Prepare GUI** (**Figure 3.1**).

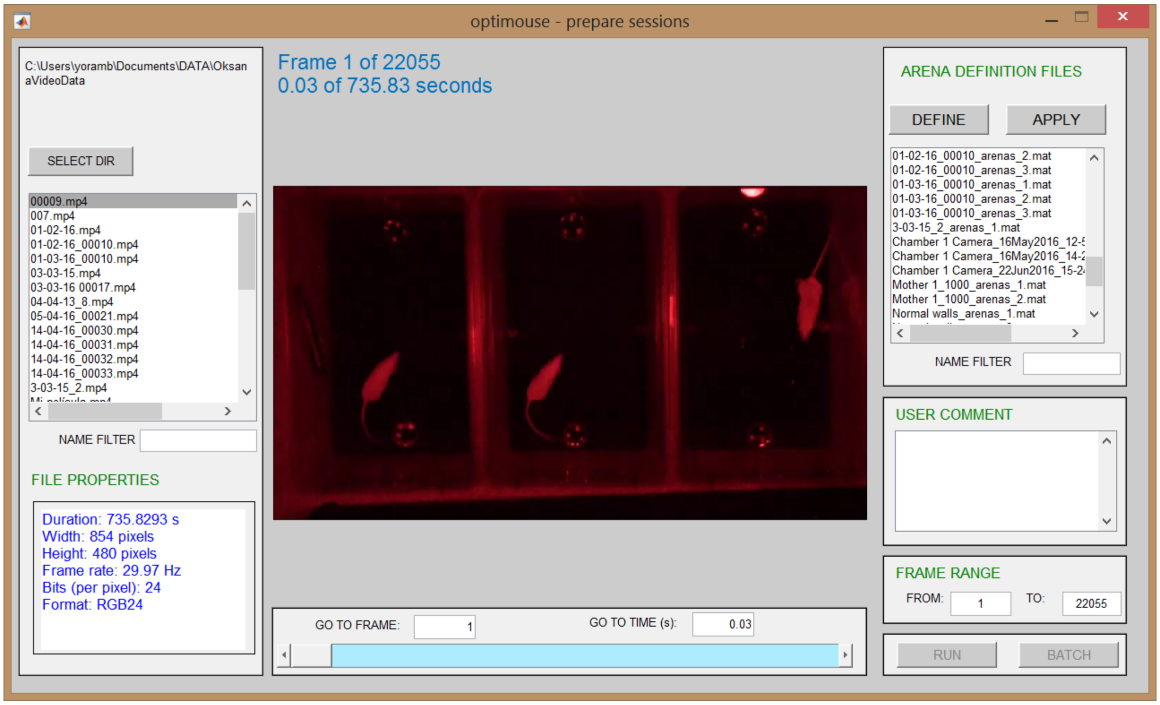

Figure 3.1. The Prepare GUI

3.1. Selecting VIDEO files

Setting the main video directory

When running OptiMouse for the first time, the main video directory must be specified. This is the directory that contains the video files. Use the **SELECT DIR** button to specify the directory. Note that for convenience, several OptiMouse GUIs have a **SELECT DIR** button. They are all designed to select the same main video directory. Once selected with any of the GUIs, it need not be done again (unless the video files are moved to a different location).

During various processing stages, OptiMouse creates several subdirectories within this main video directory. The significance of each directory is described in **Appendix 1**, though users need not be familiar with the different directories.

After selecting the main video directory, all supported video files will be shown in the list on the left side of the **Prepare GUI.** Currently tested formats are: *mp4*, *mpg*, and *wmv*. In principle, all formats that are supported by the MATLAB *VideoReader* function should work. See the function *populate_video_file_list* to expand the list of displayed formats.

Narrowing the list of video files

It is possible to limit the range of video files displayed in the list. This is done by specifying a string in the **NAME FILTER** edit box (and pressing enter). Only files whose names contain the string will be displayed in the list.

3.2. Viewing video frames

A video file is selected by clicking its name on the list. Once selected, the first frame will be shown in the central part of the GUI. Some of the video properties will be shown in the **FILE PROPERTIES** panel.

Other frames can be viewed by entering a specific frame number (in the **GO TO FRAME** edit box), a specific time within the video (using the **GO TO TIME** edit box), or by moving the slider below the frame image. Clicking the arrows at the edges of the slider will advance in one-second steps. Clicking the central region of the slider control, (blue shaded region to the right and left of the slider bar) results in larger, one-minute, steps. The current frame and time are indicated above the image.

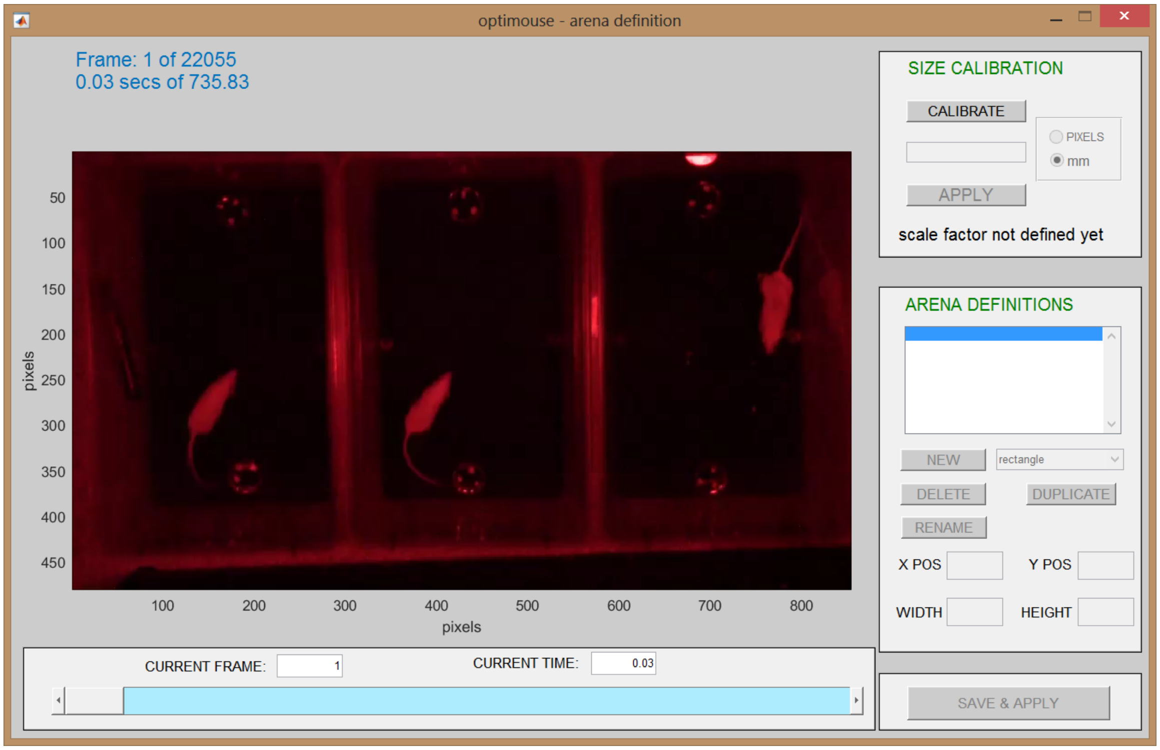

Figure 3.2. The Arena Definition GUI.

3.3. Defining Arenas

Before a video can be analyzed, one or more spatial regions of interest, denoted as *arenas*, must be defined. The list on the right side of the **Prepare GUI** contains arena definition files. Initially, the list will be empty. Arenas are defined for each video separately (but may be applied from one to another, see **Section 3.3.2.5**). To define arenas, press the **DEFINE** button. This will open the **Arena Definition GUI** (**Figure 3.2**) which performs two functions: calibration of arena dimensions and definition of its boundaries.

3.3.1. Calibrating video dimensions

The actual size of the objects in the video cannot be determined from the video alone. Thus, the ratio between pixels and mm must be specified. This is known as *calibration*. Before calibration, dimensions are specified in pixel units (**Figure 3.2**). It is assumed that scaling is uniform across the entire image and across all frames in the video.

Calibration is initiated using the **CALIBRATE** button. When pressed, a yellow square appears over the video image (**Figure 3.3**). The calibration square can be *repositioned* by placing the cursor inside it, holding the left mouse button, and dragging it. The square can be *resized* by dragging its corners or edges using a left mouse button click. The square should be positioned to cover an area of the image of known actual dimensions. This can be done with any frame in the video. Once the square size has been adjusted, its value **in millimeters** should be entered in the edit

box in the **SIZE CALIBRATION** panel. During definition, the edit box is highlighted in yellow (**Figure 3.3**).

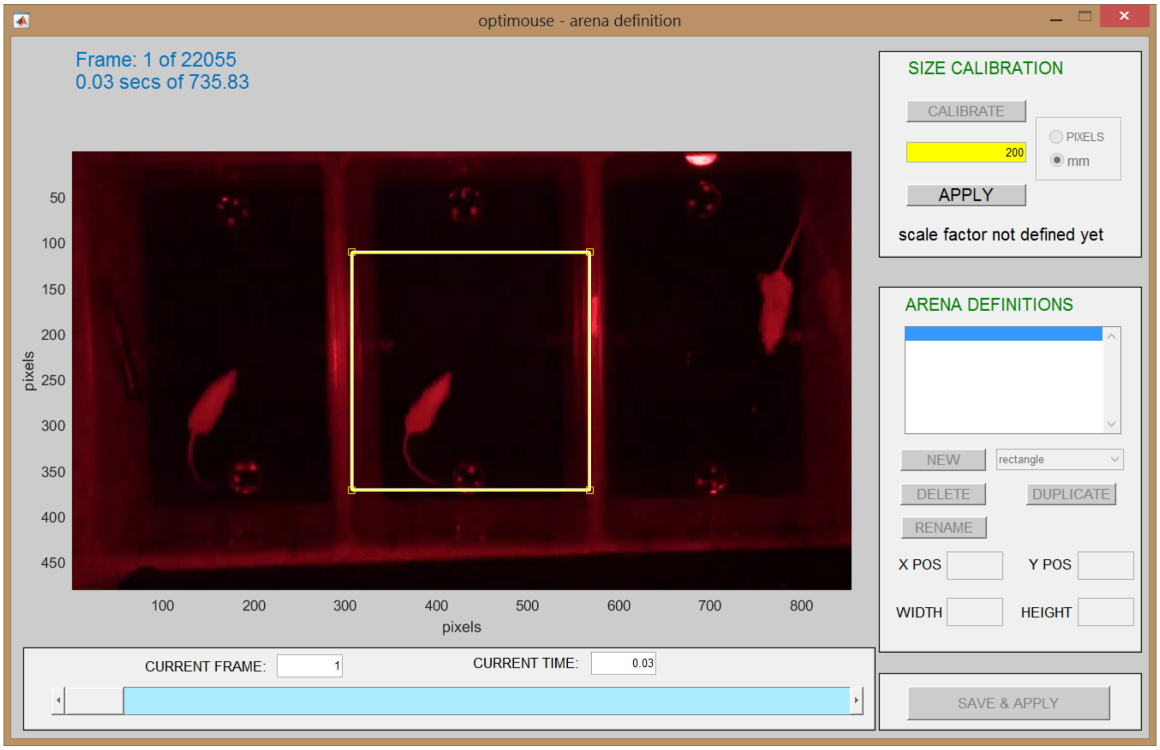

Figure 3.3. **Arena definition interface after the calibration square has been repositioned, resized, and after entering its dimensions in mm.**

Calibration is completed by pressing the **APPLY** button. Once complete, the yellow square disappears, the scale factor is set and is indicated in the GUI (in red font), and the dimensions are shown in mm (**Figure 3.4**). Dimensions can be shown as either pixels or mm units, using the radio buttons on the **SIZE CALIBRATION** panel.

It is important to examine the dimensions assigned to the image, and validate the accuracy of calibration. Although the exact dimensions do not affect most aspects of image analysis and positional detection, they are required to obtain accurate measures of distance and speed.

The scaling process can be repeated in the same interface, overwriting any previous calibration.

Once the video is calibrated, buttons in the **ARENA DEFINITIONS** panel become active (as in **Figure 3.4**).

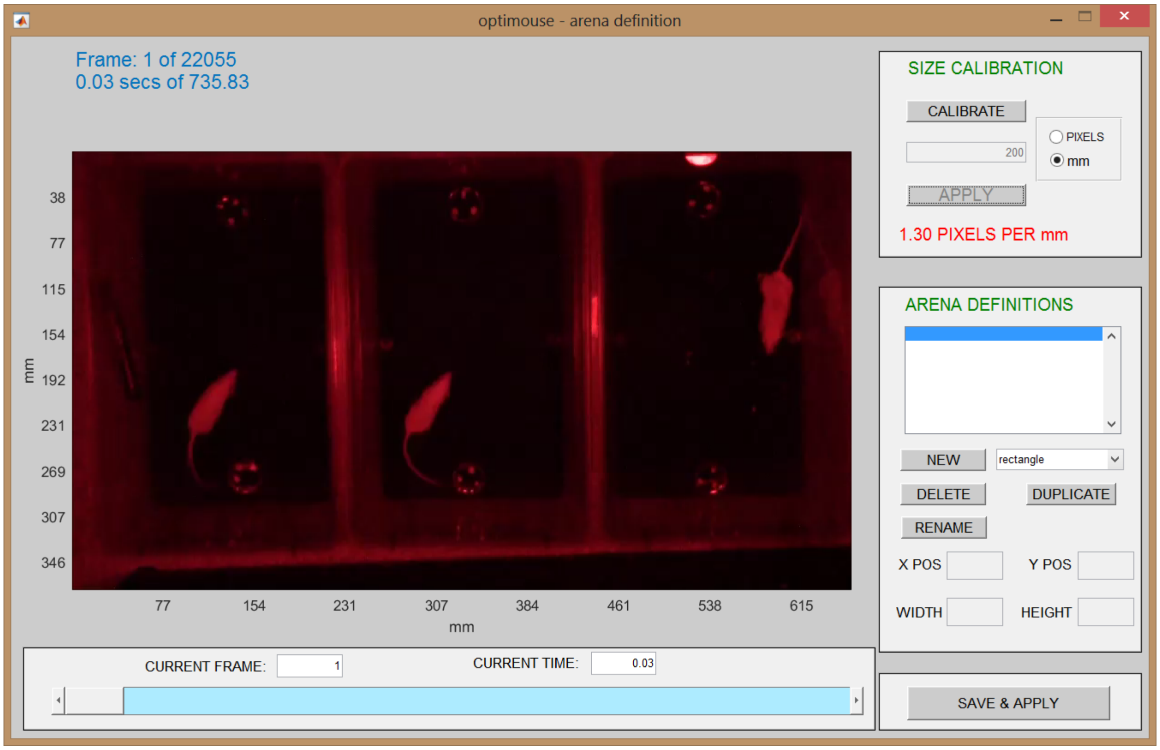

Figure 3.4. Arena definition interface after calibration.

3.3.2. Defining Arenas

Arenas are defined with the **ARENA DEFINITIONS** panel. Arenas may be circles, ellipses, squares, rectangles, polygons, or freehand forms. The list of defined arenas is shown in the listbox on the right side. To define a new arena, press the **NEW** button. The arena type is determined by the value selected in the drop down menu to the right of the button (e.g. rectangle in **Figure 3.4**).

3.3.2.1. Defining square, rectangular, circular or elliptical arenas

For arenas that are square, rectangular, circular or elliptical, pressing the **NEW** button immediately draws the corresponding shape over the image. Each of these shapes can be moved by placing the cursor inside it, holding the left mouse button, and dragging it. These shapes can be resized by dragging the corners or edges using a left-click of the mouse button.

Multiple arenas can be defined per video. For example, the video shown in **Figure 3.4** includes three parallel sessions, and thus requires specification of three arenas. Once an arena is defined, it becomes the *active* arena, and is shown in red. Arenas also become active when selected via the listbox, or when moved or resized (by clicking and dragging as described above). Merely clicking an arena, however, without moving/resizing it, will not make it active.

Rectangular, square, circular and elliptical arenas can also be resized and moved using the edit boxes at the lower part of the **ARENA DEFINITIONS** panel. Values entered into the edit boxes are applied to the currently active arena.

3.3.2.2. Defining polygon and freehand Arenas

Polygon and freehand shapes allow definition of arenas of arbitrary shapes. Polygons are particularly convenient as they allow changing the position of each vertex after definition is completed. The definition of polygon and freehand arenas follows a different procedure from that described above.

Polygon arenas

For polygon arenas, clicking, the **NEW** button does not immediately create a shape. Instead, the cursor changes to a cross when it is positioned on the image. Each left button mouse click defines a new vertex. To complete definition, the polygon must be closed in one of three ways: double clicking the left mouse button, single clicking the right mouse button, or single clicking of the left mouse button with the cursor positioned over the first vertex. Closing the polygon does not yet complete its definition. To do that, the shape must be double clicked once more. Only then it will be added to the list, and become active (and shown in red). The polygon can always be modified by moving it (placing the cursor inside it, clicking and dragging) or by dragging any of the vertices.

Freehand Arenas

For the freehand arenas, after the **NEW** button is pressed, the cursor is dragged to trace a path. When the mouse button is released, the shape is automatically closed. As with polygons, the shape must be double left-clicked to complete the definition, and make the arena active.

The definition of polygon and freehand shapes can be cancelled before completion by pressing the escape key. The size and position edit boxes are not applicable to polygon and freehand shapes. However, when active, the center coordinates will be shown in the **X POS** and **Y POS** boxes.

3.3.2.3. Renaming, deleting, and duplicating arenas

Arenas can be renamed using the **RENAME** button, deleted using the **DELETE** button, or duplicated using the **DUPLICATE** button. Pressing the **RENAME** button will initiate a dialog box requesting a new name. Duplication results in the creation of an identical arena, (but with a different name) exactly above the arena that was duplicated (thus hiding it). Renaming, deletion, and duplication of arenas is always applied to the selected (red) arena.

To demonstrate the variety of possible arena shapes, **Figure 3.5** shows the **Arena Definition GUI** after the definition of an unreasonable number of nonsense arenas.

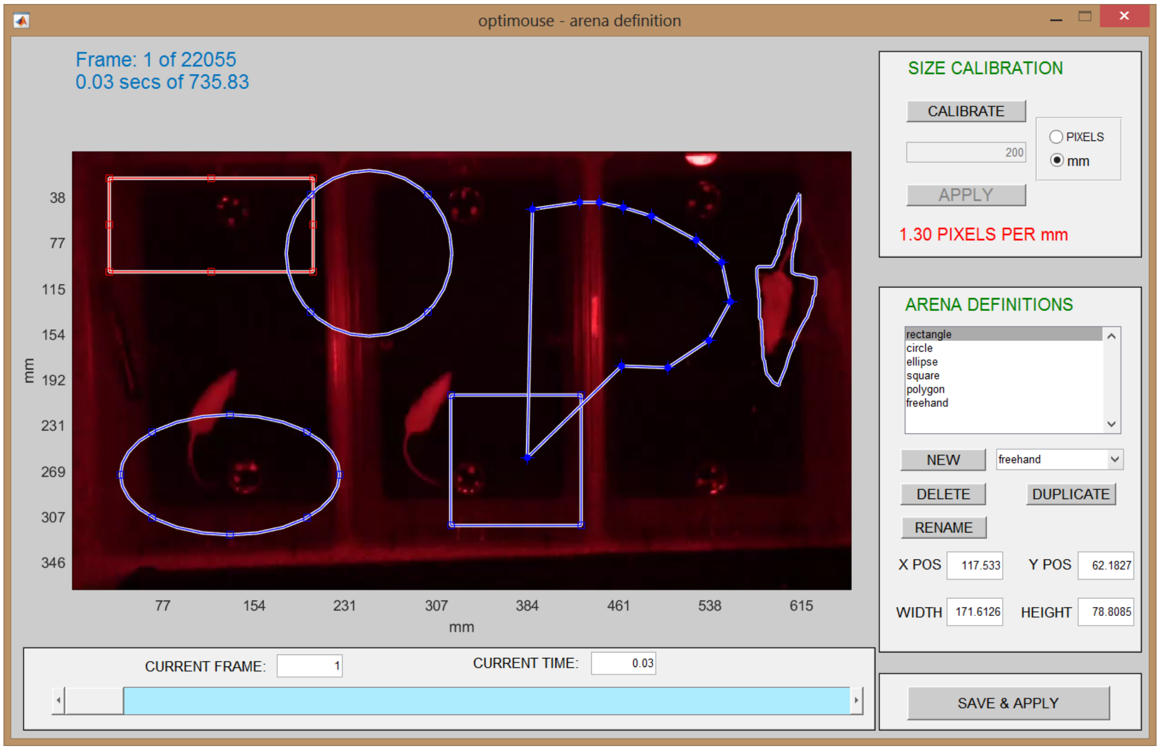

Figure 3.5. Arena definition with too many arenas.

Figure 3.6 shows a more reasonable definition of three polygon shaped arenas that were named according to their position. These examples show that a given video may contain multiple arenas, which may or may not overlap. ***Note that in subsequent steps, arenas are processed independently of each other.***

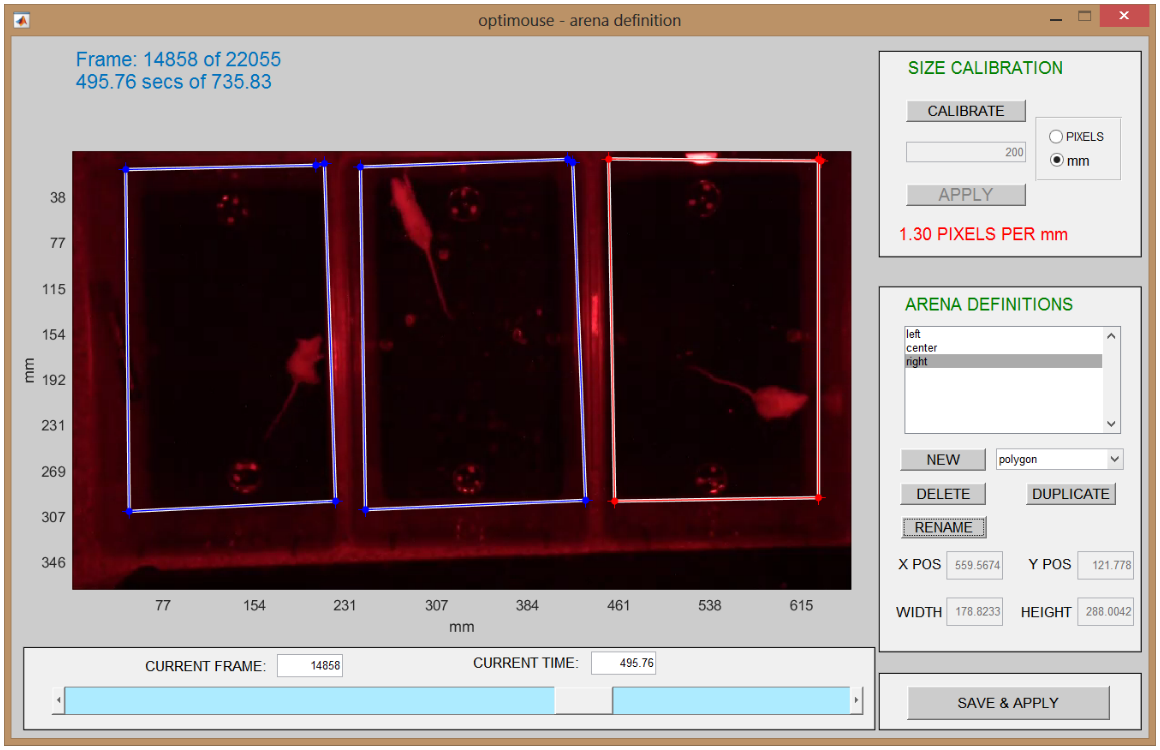

Figure 3.6 Definition of three arenas

3.3.2.4. The importance of accurate arena definitions

Proper arena definition is critical. Ideally, the arena should contain *all* the regions that a mouse (including its tail) may be in and *only* those regions. Irrelevant regions may at best increase file size, and at worst contain moving objects that will confound detection. Inclusion of arena walls that contain reflections should be avoided when possible, as these can confound detection. The navigation tools in the **Arena Definition GUI** (edit boxes, and slider bar) allow browsing different frames to confirm the validity of arena definitions. Remember that the slider can be clicked in one second and one minute steps (**section 3.2**).

3.3.2.5. Saving arena definitions

The **SAVE & APPLY** button saves arena definitions in a MATLAB data file (.mat), within a directory named ***arenas*** in the main video file directory (see **Appendix 1**), and closes the **Arena Definition GUI**.

The arena definition file name includes the original video file name, the string *arenas*, and a serial number. For the video used in this example, named 00009, the first arena file will be named 00009_arenas_1.mat. Subsequent arena files for the same video, if created, will be given names 00009_arenas_2.mat, 00009_arenas_3.mat, etc.

If the **Prepare GUI** is still open, the arenas definitions will be automatically applied to it. The arenas will be drawn over the video images, and the names given to them during definition will be indicated (**Figure 3.7**).

Arena definitions made with one video *can* be applied to another, but only if they share the same image height and width (in pixels). To apply existing arena definitions to the currently selected video, select an arena file (listbox on the right), *and* press the **APPLY** button.

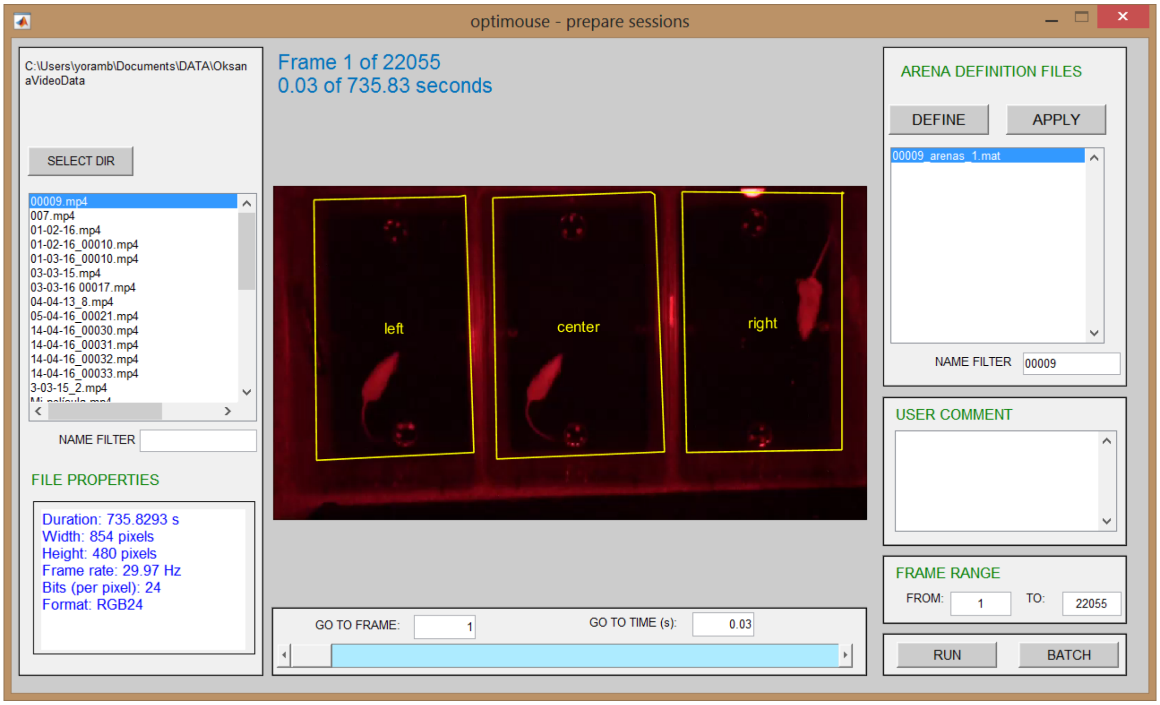

Figure 3.7 Video after applying three arenas.

3.4. Setting the range of frames for analysis

The **FRAME RANGE** panel specifies the range of frames for processing. By default, all video frames are included. However, when the relevant behavioral session is embedded in a longer video, it is best to exclude irrelevant flanking frames. The video can be examined using the navigation tools in the GUI.

3.5. Preparing videos for analysis - sessions

Once a video file is associated with an arena file (as indicated by the arenas shown over the video, as in **Figure 3.7**), sessions can be prepared using the **RUN** button. A session is defined by a video file, the arena, and the range of frames specified for analysis. For example, if the arena file contains one arena, then one session will be created. If, as in **Figure 3.7**, the arena file contains three arenas, three sessions will be created.

The technical details behind the session preparation stage are explained in **Appendix 2**. Broadly, preparation involves two major elements: 1, extraction of the spatial (arena) and temporal (frame range) boundaries, and 2, transformation of video files to MATLAB data files containing greyscale intensity values.

The specified frame range applies to all simultaneously processed sessions. If different arenas within one video are used at different times, they should be prepared separately. In other words, the **RUN** button should be pressed separately for each session, as defined by its arena and temporal range.
During session preparation, a progress bar is shown. Processing can be cancelled by deleting the progress bar. Once session definition is complete, it is possible to proceed to the detection stage (**Section 4**).

3.6. User comments

The **USER COMMENT** panel allows association of sessions with an optional text comment. The comment will be applied to all simultaneously created sessions. It will be visible in GUIs of subsequent analysis stages. **Figure 3.8** shows the GUI after setting the frame range and entering a user comment.

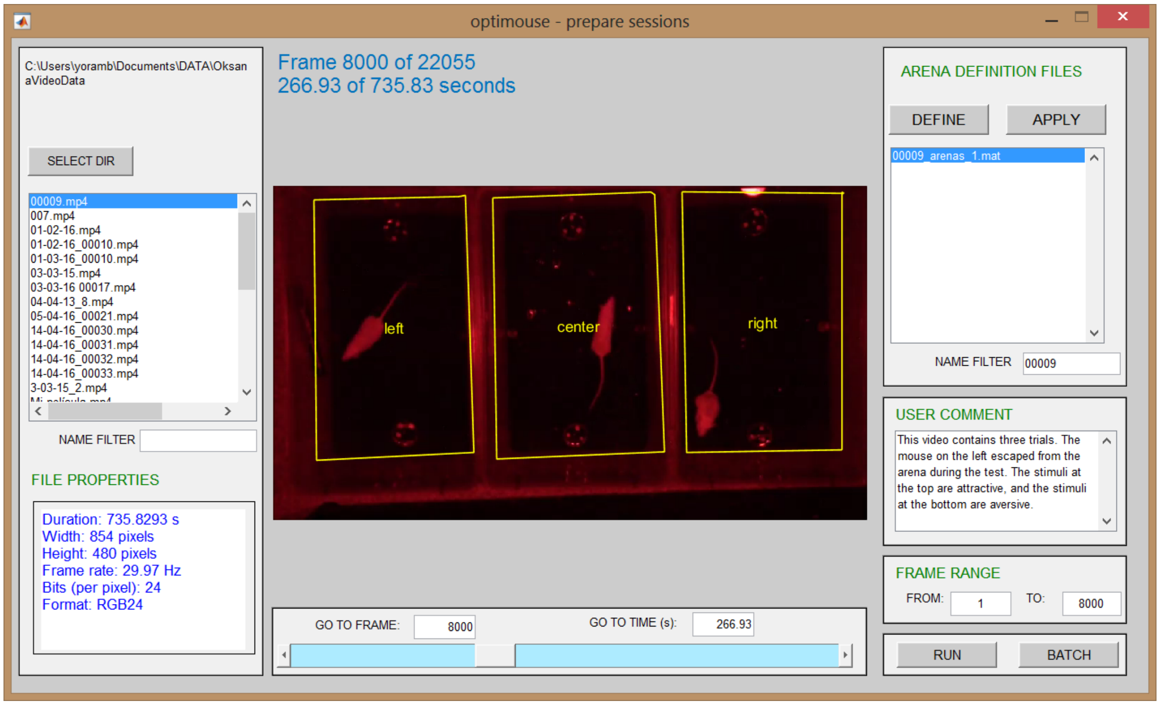

Figure 3.8 Prepare GUI after specifying frame range and entering a user comment

3.7. Preparing sessions in Batch mode

Session preparation time scales linearly with the number of arenas and frames, and depends on video resolution and arena size. Because it can be time consuming, OptiMouse allows storing commands for the creation of multiple sessions, and then executing them all as a single batch process. Pressing the **BATCH** button (rather than the **RUN** button), writes the command for session preparation into a MATLAB text file (*prepare_arena_batch.m*) located in the *optimouse_user_definitions* folder. The batch file is run by typing its name on the MATLAB

command line (>> prepare_arena_batch). Progress made during batch file execution is reported on the command line.

There is no limit to the number of commands in the batch file. With *each* press of the **BATCH** button, commands are added to the batch file. Thus, pressing the button repeatedly will create duplicate copies of the same commands in the file. This should be avoided, since it results in execution of the calculation multiple times. In the same context, even after execution is complete, commands remain in the batch file. It is the user's responsibility to delete or convert to comments any previously executed commands. Thus, before running the batch file it must be examined to confirm that it contains the intended commands. See **Appendix 3** for an example of a batch file and a description of its contents.

4. Setting Detection Parameters

In this stage, parameters for detection of body and nose positions are specified. This is done using the **Detect GUI** (**Figure 4.1**) which is opened by the **DETECT** button in the **OptiMouse GUI**.

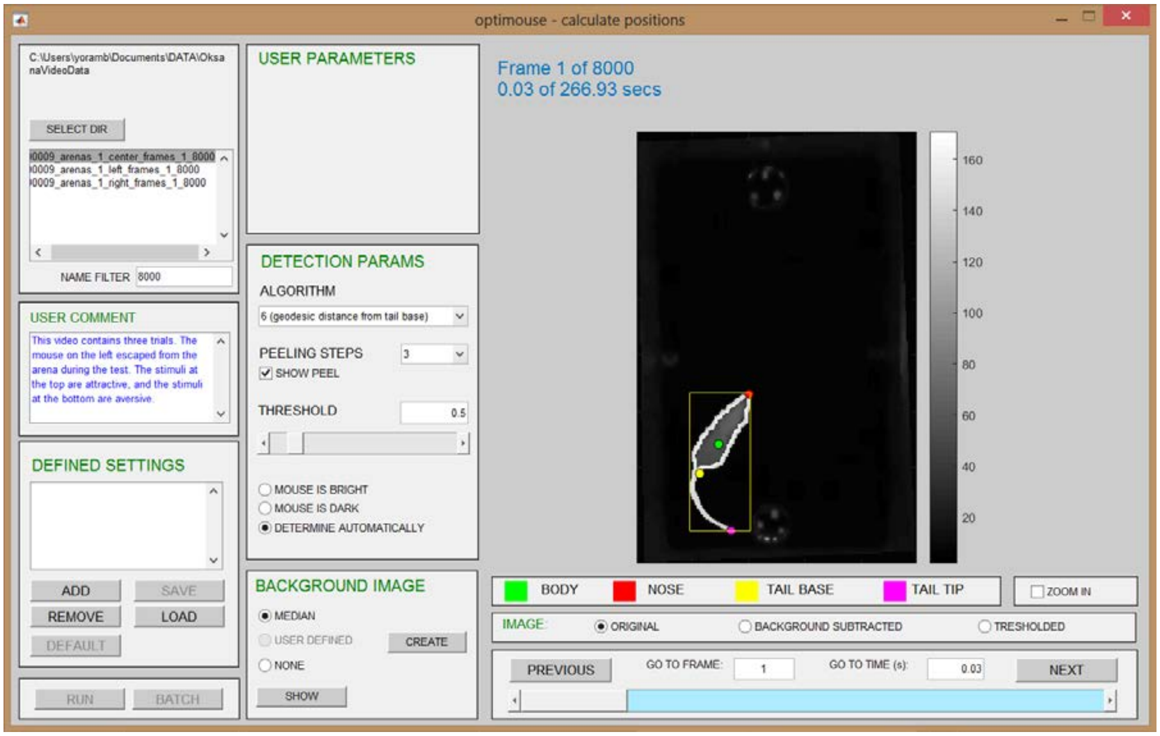

Figure 4.1 The Detect GUI

4.1. Selecting sessions for viewing

The listbox on the upper left of the **Detect GUI** shows all previously created sessions. Each entry in the list corresponds to one session. Sessions are identified as directories within the main video directory containing the string **_arenas** (see **Appendix 1**). The name of the session contains

information about the original video file name, the arena definition file, the arena name (given during definition), and the frame range used. For example, the session highlighted in **Figure 4.1**, was created from video **00009**, the arena file **arenas_1**, an arena named **center**, and includes frames **1** to **8000.** As with all other GUIs, the list can be filtered using **NAME FILTER** edit box. When a session is selected, the first frame will be shown. If a user comment was entered in the previous stage, it will be shown in the **USER COMMENT** panel.

4.2. Viewing the video

The right side of the **Detect GUI** shows frames from the session. At this stage, the frames are a greyscale and are clipped from the original video. The detected positions in each frame are indicated by landmarks plotted over the image (**Figure 4.1**). The color scale to the right of the image shows the intensity values of the image. The yellow rectangle bounds the mouse (or whatever is detected as one). Colored circles indicate specific body positions. A legend is shown below the image. For example, **BODY**, shown in green, corresponds to the center of mass of the mouse (the detected object).

4.2.1. Zooming in on the mouse image

The zoom-in option, to the right of the color legend, will show a magnified view of the rectangular bounding area containing the mouse (**Figure 4.2**).

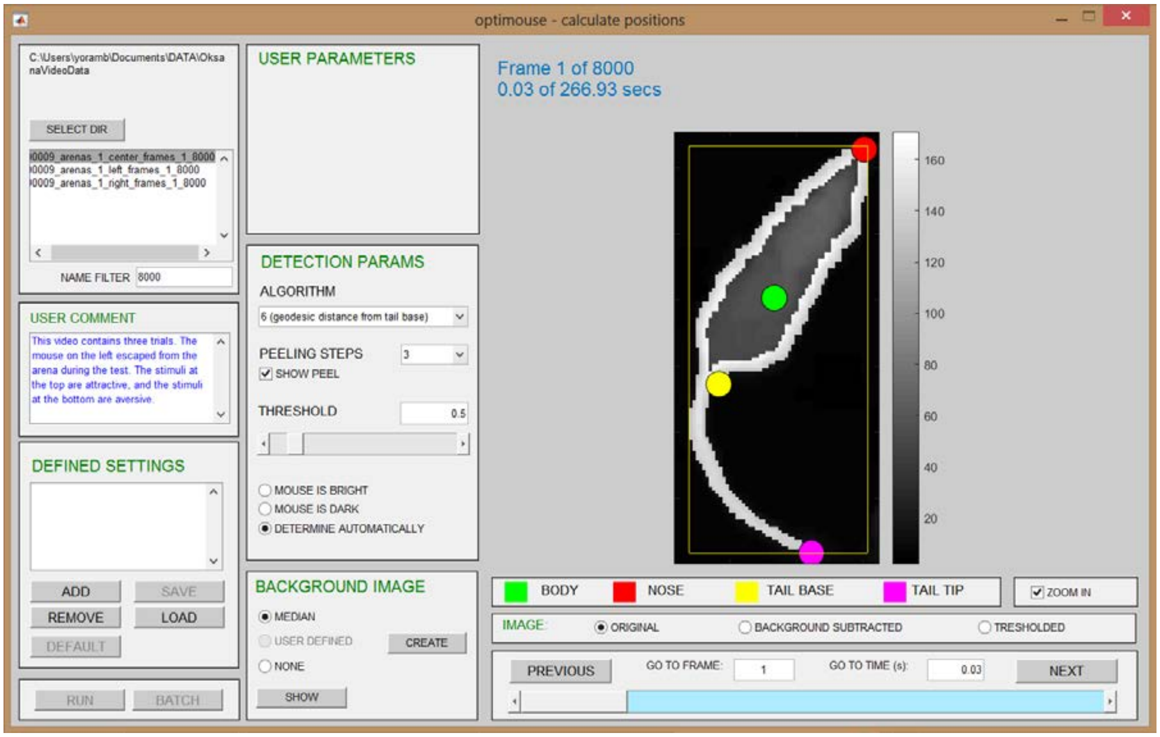

Figure 4.2 The Detect GUI – zoomed in

4.2.2. Navigating the video

Controls in the bottom right panel allow navigation. In addition to the **GO TO FRAME** and **GO TO TIME** edit boxes and the slider, the **Detect GUI** includes buttons for moving to the **NEXT** and the **PREVIOUS** frames. These operations can also be performed using the keyboard: The **>** (right arrow), and **<** (left arrow) keys move to the next and previous frames, respectively. If the ALT key is held while pressing the arrow keys, one second steps will be made. Keyboard controls will not function if the cursor is within one of the edit boxes. See **Appendix 6** for a list of keyboard shortcuts.

4.3. Understanding body and nose detection

A basic familiarity with the detection process is required to understand the **Detect GUI** controls. Position detection begins with object identification based on the different intensity values for mouse and background. This involved *background subtraction*, and thresholding to define the image. Radiobuttons in the **IMAGE** panel, below the video image, can be selected to show the original, the background subtracted, or the thresholded image (see **Figure 4.3** for these various views).

4.3.1. Subtraction of a median background image

Background subtraction is helpful because it eliminates static objects in the arena that could be confounded with the mouse. By default, the median image is subtracted from all video frames. The median image is calculated during session preparation, over the entire range of the session. If the mouse's position varies in different frames, it will be filtered-out in the median image, and the median will provide a good estimate of the arena *without* the mouse. However, if the mouse stays immobile in many frames, it will also appear in the median image, and this could seriously confound detection. Solutions for this case are described in sections **4.3.2** and **4.3.3**. The **SHOW** button in the **BACKGROUND IMAGE** shows the currently used background image (**Figure 4.3D**).

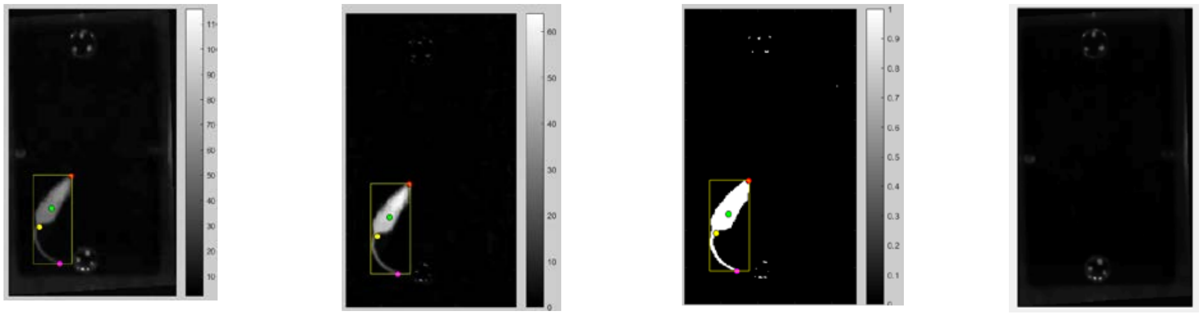

A. Original image **B.** Background subtracted **C.** Thresholded image **D.** Background

Figure 4.3. Different views of the video frames. Only a section of GUI is shown. For this view, the SHOW PEEL checkbox has been unchecked (section 4.5).

4.3.2. Cancelling Background subtraction

If the background itself changes during the session (due to changes in the position of objects, lighting conditions, or camera movement) subtraction of a constant background will actually interfere with correct detection. In such cases, it may be better to avoid background subtraction. To cancel background subtraction, check the **NONE** radiobutton in the **BACKGROUND IMAGE** panel. The cost of avoiding background subtraction is minimal if the mouse is clearly distinct from the background.

4.3.3. Defining a custom Background using the Custom Background GUI

If the median image does not yield a good image of the arena without the mouse, it may be possible to create a good background image from a specific set of frames, from either the current video or another. Definition of a custom background is accomplished with the **CREATE** button in the **BACKGROUND IMAGE** panel. This will open the **Custom Background GUI** (**Figure 4.4**). When called, the **custom background GUI** will show the video containing the current session. The boundaries of the arena and the rectangular region containing it are plotted over the video (yellow and green in **Figure 4.4**).

The listbox on the left side of the GUI allows choosing other videos for background images. Other videos can be used only if they share pixel dimensions with the original video. Only videos that were filmed under the exact same conditions as the original video should be considered for background definition.

The right side of the GUI shows the currently defined background image. Initially, it is empty. A background can be defined from the selected video by one of two methods:

4.3.3.1. Creating a background from a single frame

The **CREATE FROM CURRENT** button will clip the arena boundaries from the current frame (in the **Custom Background GUI**) and apply it as a background image. The resulting image is shown on the right panel. If all session frames contain the mouse, this option will not yield a good background image.

4.3.3.2. Creating a background from a range of frames

A background image can be defined as a median of any subset of frames in a video. The goal is to find a set of frames whose median will include the arena without the mouse. The subset of frame is specified in the edit box above the **CREATE FROM CURRENT** button. The set of frames can be specified using any valid MALTAB array syntax. For example: **1:10** specifies all frames between 1 and 10, **1:1000:22000** specifies every thousandth frame. Strings such as **1:20 200:100 1:3** are also valid. The resulting image, which may require a few seconds to calculate, will be shown in the right side of the GUI (see **Figure 4.4**).

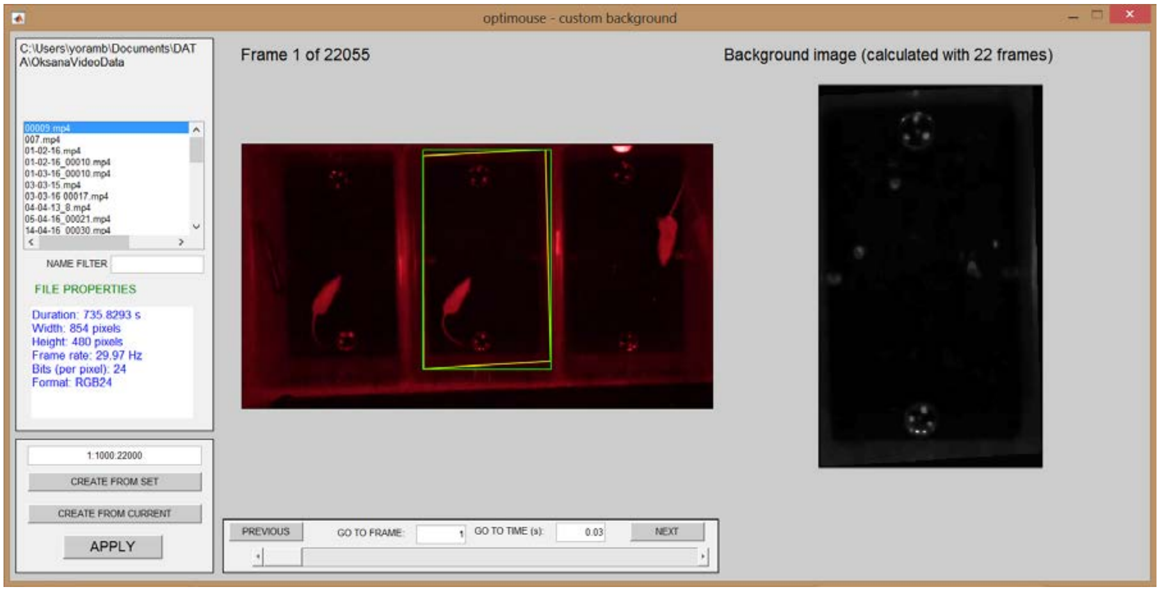

Figure 4.4 The custom background GUI

4.3.3.2. Applying the custom background image

Once a background image is created using either of the two methods, it can be applied to the current session using the **APPLY** button in the **Custom Background GUI** (the button only becomes active after an image is specified). After pressing the **APPLY** button, the **USER DEFINED** radiobutton in the **BACKGROUND IMAGE** panel of the **Detect GUI** will become accessible. However, to apply the custom background it is necessary to select the radiobutton. The custom background image can be viewed with the **SHOW** button in the **BACKGROUND IMAGE** panel.

4.4. Distinguishing mouse from background

After background subtraction, frames are transformed to binary images after thresholding. Pixels with intensity above the threshold are designated as 1, the rest are designated as 0.
Checking the **TRESHOLDED** radiobutton in the **IMAGE** panel will show the thresholded image in the GUI (**Figure 4.3C**). *The thresholded binary images serve as inputs to the detection algorithm, and thus represent a key stage in the detection process.*

4.4.1. Setting the brightness of the mouse relative to the arena

To separate mouse from background, it should be known if the mouse is brighter or darker than the arena. OptiMouse attempts to determine this automatically (the **DETERMINE AUTOMATICALLY** button in the **DETECTION PARAMS** panel is selected by default). This determination can fail however, and when it does, object detection will also fail. In such cases, users should explicitly specify if the mouse is darker or brighter by checking either the **MOUSE IS DARK** or the **MOUSE IS BRIGHT** buttons.

4.4.2. Setting the threshold value

The threshold is determined automatically for each frame by the MATLAB function **graythresh**. However, **graythresh** does not always return a value that separates mouse from background well. Therefore, this automatically determined threshold can be modulated using the **THRESHOLD** edit box, or slider, in the **DETECTION PARAMS** panel. The user-defined **THRESHOLD** value acts as a multiplicative factor over that determined automatically. The only relevant criterion for setting the **THRESHOLD** is the result of object detection. Ideally, the threshold should separate the entire mouse, and nothing but the mouse, from the background. Varying the threshold directly effects the dimensions of the detected object. Note that in all built-in algorithms, the mouse is considered to be the largest object in the arena. Thus, the presence of above threshold non-mouse pixels is not a problem as long as they do not form a continuous patch larger than the mouse.

Excessively high thresholds will preclude object detection and a warning message will be displayed above the frame image. Excessively low threshold values could lead to detection of objects or regions that are not the mouse.

4.5. Detecting body and nose positions - the peeling operation

Once mouse boundaries are properly determined, deriving the body center is straightforward and robust (**BODY**, indicated by the green circle in **Figure 4.1**). Technically, the body center is determined as the center of mass of the mouse object.

Detection of the nose is far more challenging and therefore OptiMouse includes several methods to derive nose position from the mouse image. In addition, OptiMouse supports inclusion of custom algorithms, implemented as MATLAB code files (see **Section 4.11**).

Most built-in algorithms use a common procedure. Detection of the nose is actually based on detection of the tail. Tail identification is based on a “peeling” operation. In each stage of peeling, the pixels on the margins of the mouse are removed. Because the tail is thinner than the head, it will disappear after several cycles of peeling, and a “tailless” mouse will remain. The difference between the tailless mouse and the original mouse allows OptiMouse to identify the tail.

The number of peeling cycles is an important determinant of proper nose position detection. Too few peeling steps will not eliminate the entire tail; too many cycles will eliminate the entire mouse. Ideally, peeling should consistently remove the tail, but leave the body intact. The actual value depends on the dimensions of the mouse (as it appears in the binary image) in pixels.

This number of peeling cycles is set in the **PEELING STEPS** drop down menu. **Figure 4.5** shows the effects of varying the number of cycles. The **SHOW PEEL** checkbox allows viewing or hiding the peel from the mouse image.

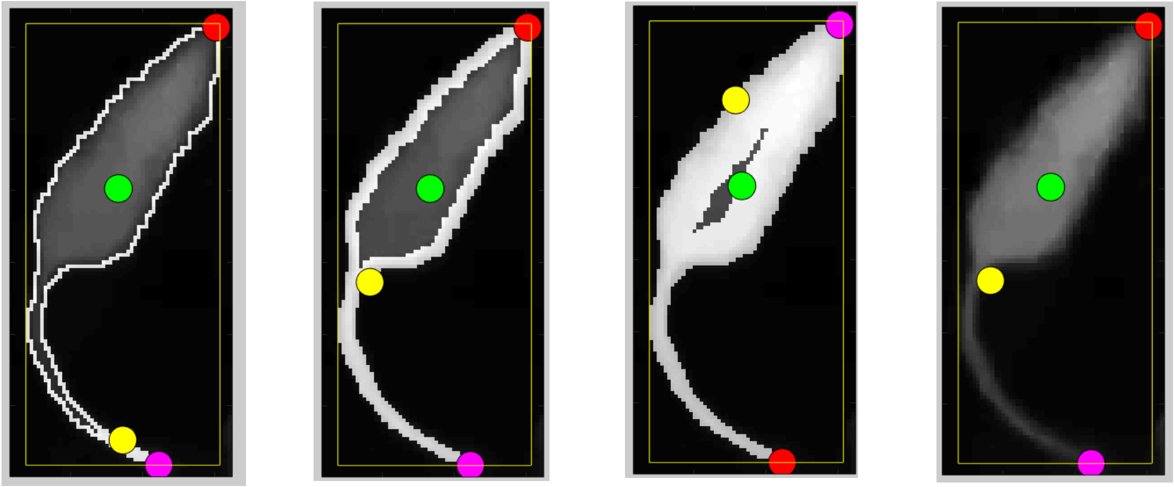

1 peeling cycle 3 cycles 9 cycles 3 cycles, peel hidden

Figure 4.5 **Peeling cycles. Shown are zoomed in images of the mouse after varying numbers of peeling steps. Although both one and three cycles result in correct nose detection (red circle), the choice of three is the correct one, because it peels the entire tail and correctly identified the tail base (yellow). An excessive number of cycles (9) results in confounding nose and tail. If the peeling cycles are increased further, detection will not be possible as nothing will remain after peeling. The image on the right corresponds to detection with three cycles, with the SHOW PEEL checkbox unmarked.**

4.6. Detection algorithms

OptiMouse includes different built-in algorithms for detection of nose positions. All algorithms accept as inputs the binary mouse image and the number of peeling cycles. All algorithms detect the body center identically, but differ in how they determine nose positions. The algorithm is selected with the **ALGORITHM** pull down menu in the **DETECTION PARAMS** panel. Algorithms are briefly described below. All algorithms are implemented in the MATLAB function **get_mouse_position_mm.m**.

Algorithm 1: The nose is defined as the point on the mouse perimeter that shows the largest distance difference between the tail center of mass and the tailless (peeled) mouse center of mass.

Algorithm 2: Like algorithm 1, but the nose must be on the boundary of the bounding box (shown in yellow).

Algorithm 3: The nose is defined as the furthest point from the tail center of mass and must also be on the bounding box (yellow).

Algorithm 4: The nose is defined as the furthest point from the tail *end* (Euclidean distance).

Algorithm 5: The nose is defined as the furthest point from the tail *base* (Euclidean distance).

Algorithm 6: Like algorithm 5, but the distance to the base is determined along a path on the mouse boundary. This is known as the *geodesic* distance, and is particularly advantageous when the tail is curved.

Algorithm 7: Appropriate when the tail is not visible so that the most angular region of the mouse body is the snout, rather than the tail. This algorithm defines as nose, what the preceding algorithms define as the tail.

As with all parameters, the choice of algorithm should only be determined by the success of nose detection. The landmarks shown over the image allow determination of the performance of each of the algorithms, and if they fail, identification of the cause of failure. Note that not all landmarks are applicable to all detection algorithms. For example, algorithm 7 only involves *body* and *nose*, while algorithm 4 involves *body*, *nose* and *tail tip*, but not *tail base*. The most important landmarks to note are of-course the body (green), and the nose (red).

Under most scenarios that we tested, algorithm 6 is the most reliable, and is thus the default. Algorithm 7 is very useful when the tail is not included in the binary mouse image (either because it is hidden from view, or because the threshold is set too high).

A detailed technical description of the Algorithms is provided in **Appendix 10**. See also the documentation within the function **get_mouse_position_mm.m.**

4.7. Running Detection (with a single *setting*)

The overall goal of the **DETECT GUI** is to find an optimal set of detection parameters, known as a *setting*, and then run position detection on all video frames. The *setting* includes all the user specified parameters: algorithm, threshold, whether the mouse is dark or light, number of peeling steps, and the background image (or lack of it). Finding the optimal *setting* requires evaluation of its performance on a range of frames. This is achieved by browsing the video and observing the outcome of the detection. In the process, detection parameters should be modified to achieve the best nose detection throughout the session.

Before applying any set of parameters for detection, they must be added to the settings list. This is done with the **ADD** button in the **DEFINED SETTINGS** panel. When pressed, OptiMouse prompts the user for a name for the setting and then it is added to the list. **Figure 4.6** shows the GUI after adding of one setting, named *default set*.

Once at least one setting is defined, detection can be started using the **RUN** button in the **Detect GUI**. The detection can be cancelled by closing the progress bar. Results are saved in a MATLAB data file denoted as the *position file* for subsequent analysis stages. The position file is saved in a sub-directory, named *positions* under the main video directory. The position file name retains information about the session, and also includes the string **_positions**. For example, the resulting position file for the center arena in the example of **Figure 4.6** will be named: 00009_arenas_1_center_frames_1_8000_positions. See **Appendix 1** for a description of the position file.

The position file can be directly taken for analysis (**Section 6**). However, as explained in the next sections, it is often best to define multiple detection settings and, regardless of the number of settings, to review the results of detection before analysis (**Section 5**).

4.8. Running Detection in Batch mode

Like arena preparation, position detection is a time consuming process. Therefore, the **Detect GUI** provides a batch processing option, which is similar to that for the session preparation stage. Pressing the **BATCH** rather than the **RUN** button will add a command, equivalent to that of pressing the **RUN** button, to a batch file. The batch file for the detection stage is named **calculate_positions_batch.m,** and is located in the **optimouse_user_definitions** folder.

The batch file can be run by typing its name on the MATLAB command prompt (>>**calculate_positions_batch**) or by pressing the Run icon when the batch file is open in the MATLAB editor.

The batch file is intended to be called after a number of commands have been added to it. In using the calculate_positions_batch, the same cautionary steps should be taken as when using the **prepare_arena_batch** file (Section 3.6). See **Appendix 4** for a description of the **calculate_positions_batch** file.

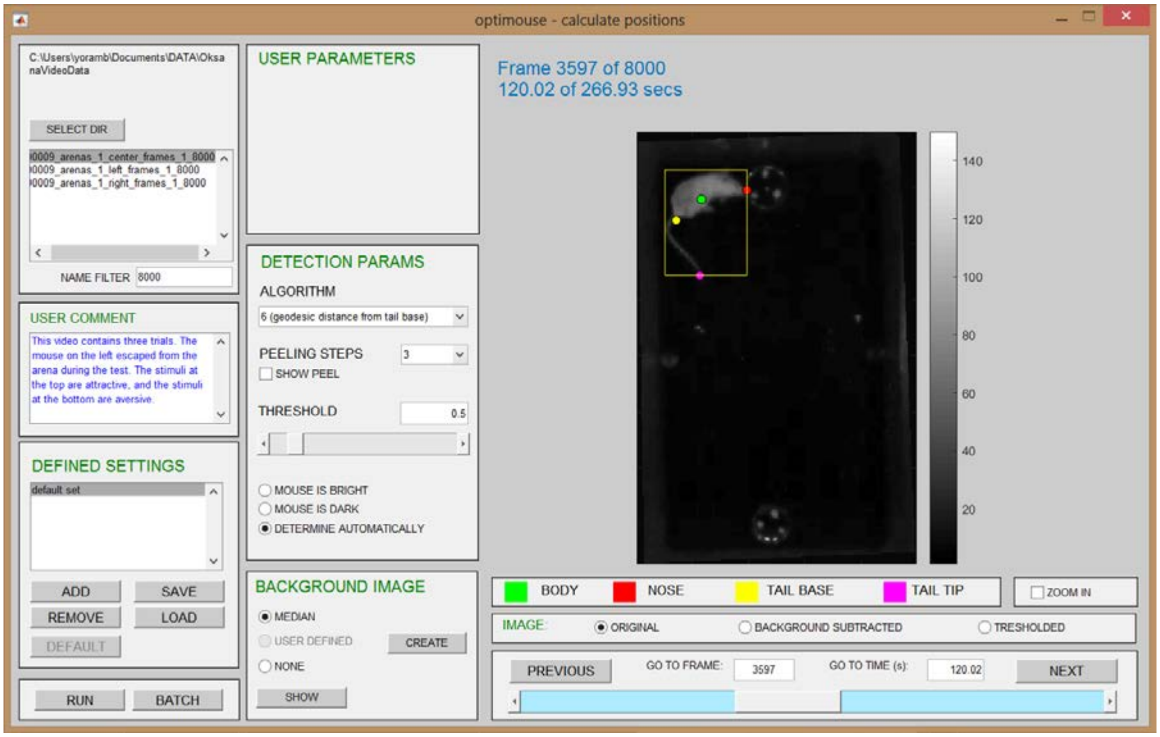

Figure 4.6 **The Detect GUI after one setting has been defined. Note the DEFINED SETTINGS panel.**

4.9. Assigning multiple settings to a single session

Generally, a given *setting* will work well only for a subset of frames in a session. For example, the tail may be visible in some frames but not in others. As another example, in sessions with heterogeneous arenas, background subtraction may be helpful in some regions, but unfavorable in others. To address such variability, OptiMouse allows the definition of up to *6 different settings* per session. The drawback of including more methods is prolonged processing time; a minor cost, especially if the batch mode is used.

4.9.1. Adding and removing multiple settings

The procedure for adding multiple settings is identical to that described for a single setting. When the **ADD** button is pressed, OptiMouse prompts for a name, and the current GUI parameters will be saved as a *setting*. Settings must be unique, both in the parameter values, and in name. The distinction between two methods may be subtle. For example, two settings could differ only in the detection threshold value, or in the number of peeling steps. For the reviewing stage (**Section 5**), it is convenient to assign meaningful names to the settings. When a setting is active (by selecting its name in the **DEFINE METHODS** panel), the parameters associated with it are applied to the currently shown frame. However, changing parameter values will not affect saved settings. To update the parameters associated with a predefined setting, press the **ADD** button, specify that setting's name, and confirm overwriting it. A setting can be removed by selecting it and pressing the **REMOVE** button.

4.9.2. Defining a *setting* as the default

When the list contains multiple settings, the first one serves as the *default setting*. The significance of this designation is explained in **Section 5**. Practically, it is convenien, to set as default the setting that applies to the largest fraction of frames. Pressing the **DEFAULT** button when a setting is selected will move it to the first position in the list, and thus make it the default.

4.9.3. Saving and loading settings

The **SAVE** and **LOAD** buttons on the **DEFINED SETTINGS** panel allow saving, and subsequently loading, settings. This allows application of settings defined in different settings. When saving, OptiMouse suggests a default name for the *settings* file, but any name may be used. It is recommended to retain the prefix *detection_settings* in the name. **Figure 4.7** shows the **Detect GUI** after the definition of three settings.

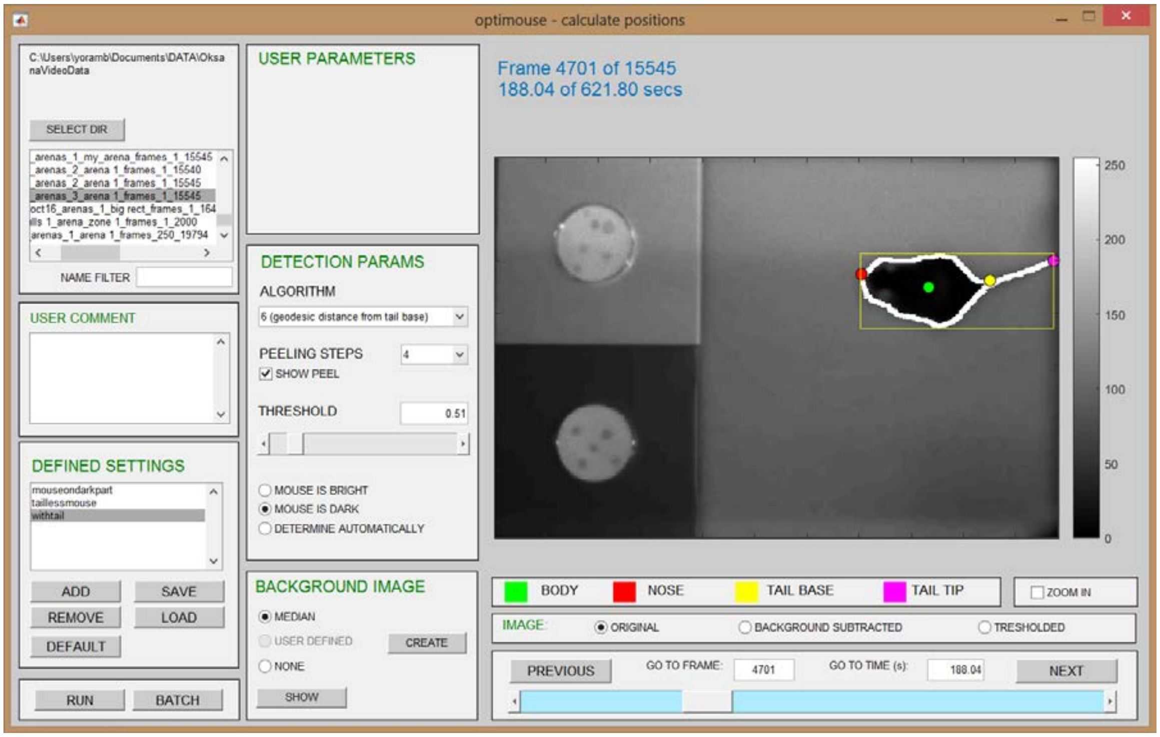

Figure 4.7 **The Detect GUI after three settings were defined. Settings' names indicate the conditions for which they are suitable.**

4.10. Running detection with multiple settings

Running detection with multiple settings is identical to running it with one setting. However, the resulting *positions* file will contain positions determined by each of the settings. Batch processing works with multiple settings as it does with a single settings (See **Appendix 4**).

4.11. Incorporating custom detection functions

Another feature that allows OptiMouse to deal with variable detection scenarios is the ability to integrate custom user-defined functions into the **Detect GUI**. User-defined algorithms may be slight variations of the built in algorithms, or entirely distinct from them. User defined functions must return body and nose positions as outputs and ideally, they should also return other parameters associated with detection. The significance of these parameters will be evident in the next stage (**Section 5**). Custom function must be defined in a designated file named *user_defined_detection_function_description.m* under the *optimouse_user_definitions* folder.

Once custom detection functions are defined, they will appear in the **ALGORITHMS** drop down list box in **DETECT PARAMS** panel. User defined functions can accept as input any of the parameters used by the built-in algorithms, and the GUI can accommodate up to five additional parameters for these function. **Figure 4.8** shows the **Detect GUI** with a user defined algorithm selected. The algorithm selected in **Figure 4.8** was associated with five additional parameters. For all intents and purposes, once defined user defined functions are treated by OptiMouse as if

they were built-in detection algorithms. A detailed explanation of the definition and use of custom detection functions is given in **Appendix 5**.

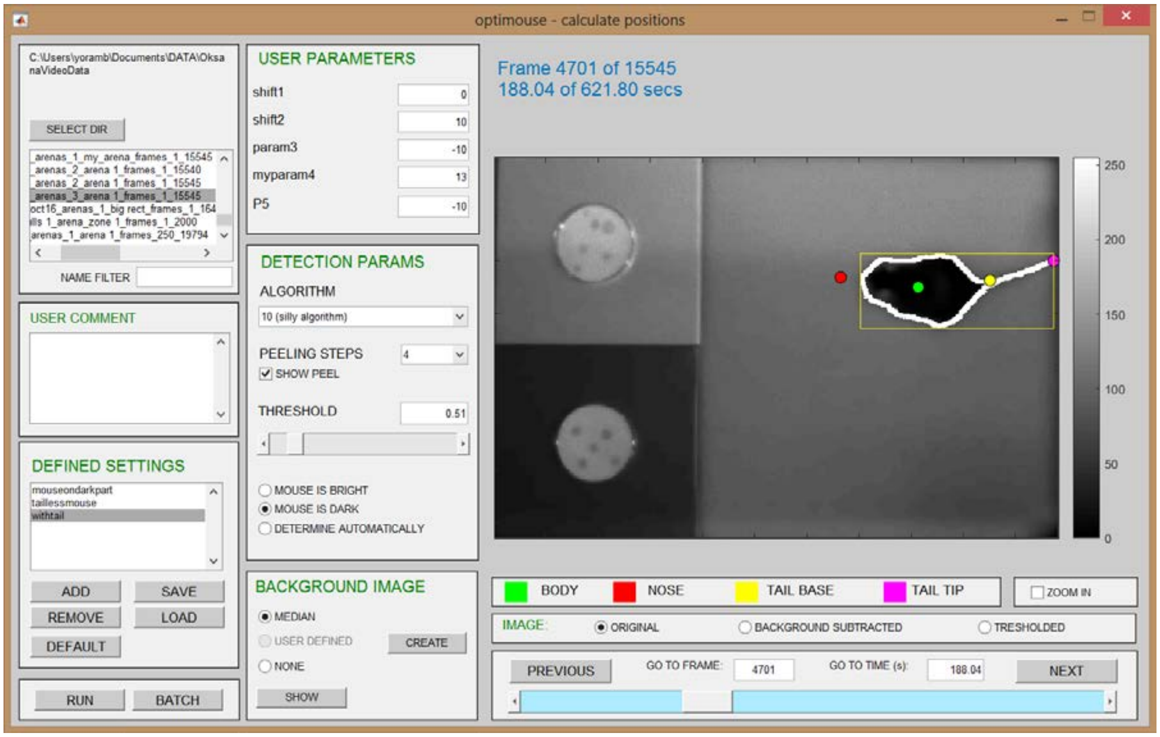

Figure 4.8. **Detect GUI with a custom algorithm selected. This algorithm merely imposes a shift on positions detected using one of the built-in algorithms (compare with Figure 4.7). It is intended only for illustration purposes.**

5. Reviewing Position Detection

The reviewing stage is initiated by pressing the **REVIEW** button in main OptiMouse interface. **Figure 5.1** shows the **Review GUI**, with a session that was processed with three detection settings (see **Figure 4.8**).

The upper left listbox shows all position files within the *positions* subdirectory (See **Appendix 1**). Position files are identified as mat files ending with the string **_positions**. As with other GUIs, a **NAME FILTER** can be entered to limit the list. The **USER COMMENT** panel shows previously entered user comments, if defined in the session preparation stage.

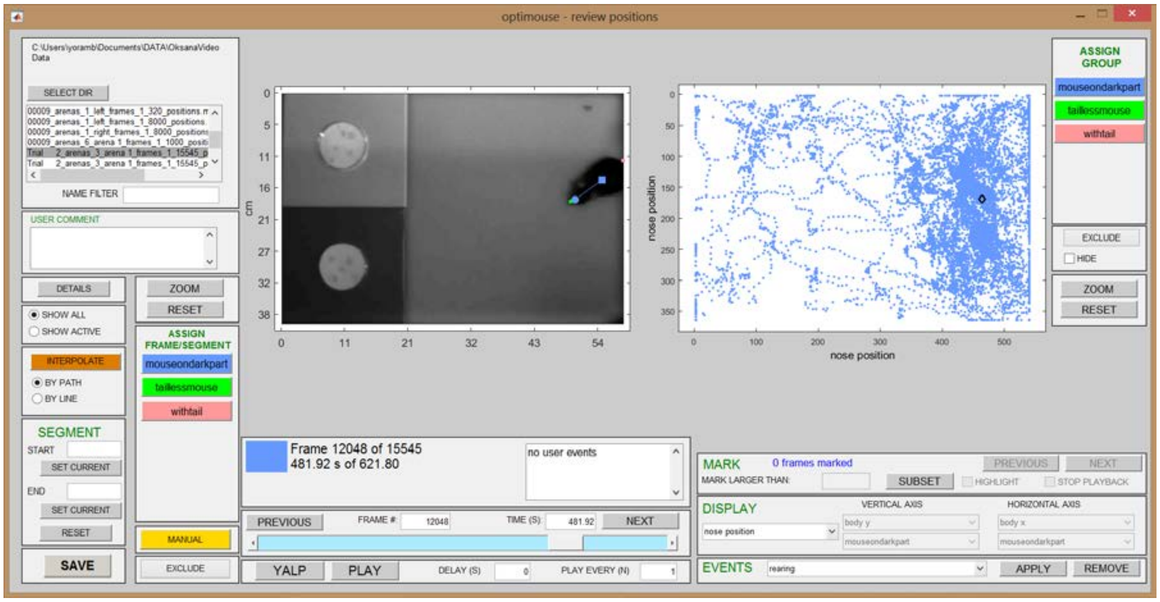

Figure 5.1. Prepare GUI, a session with 3 settings.

5.1. Browsing frames and position detection

The center of the GUI shows session frames. Frames can be accessed by specifying their number or their time into the edit boxes, or by moving the slider. Recall that a small slider step corresponds to one second, while the larger steps correspond to one minute. The **PREVIOUS** and **NEXT** buttons move to the previous and next frames. They can be activated from the keyboard using the right arrow (>) and left arrows keys (<).

In addition, the **Review GUI** includes a continuous playback option. The **PLAY** button can be toggled to play or pause movie playback in the forward direction. The **YALP** allows playback in reverse. The **DELAY** and **SKIP** edit boxes allow slow motion and accelerated playback. If a **DELAY** value is entered, playback will pause for the specified duration (in seconds) between consecutive frames. The **SKIP** value allows a quick run through the movie. For example, if the value is 10, then every 10th frame will be shown, effectively speeding up playback 10-fold. A value of 1 corresponds to normal playback. The **YALP** and **PLAY** actions can be activated from the keyboard using the left and right arrows while the **ALT** key is pressed. Pressing the left or right arrows while the **SHIFT** key is pressed moves in one-second steps.

Appendix 6 provides a summary of all keyboard shortcuts.

5.1.1. The Active Setting

In each frame, detection results from all previously defined settings are shown. For the session shown in **Figure 5.1,** three settings were defined. Each setting is associated with one color. In any given frame, there is one *active setting* which determines the final coordinates assigned to each frame. The major goal of the reviewing stage is to apply correct setting to each of the frames. As described below, this can be achieved in multiple ways.

Initially, the default setting from the previous stage (**Figure 4.8**) is applied to all frames. The position associated with the active setting is indicated by a square (body), a circle (nose) and a line connecting them. For other, non-active settings, only nose positions are shown, as smaller circles. The active setting for any given frame is also indicated by the color of the rectangle below it. Thus, in **Figure 5.1**, for frame 12048, the first setting is the active setting. This is indicated by the blue line connecting the blue square and circle (nose positions determined by the other settings are shown as smaller green and pink circles), and the blue rectangle below the mouse image.

Viewing only the active setting

By default, detection results of all settings are shown. Check the **SHOW ACTIVE** radiobutton (below the **DETAILS** button) to show the active setting only. Checking the **SHOW ALL** radiobutton will show all settings.

Viewing setting details

The **DETAILS** button, under the **USER COMMENT** panel, creates a display with complete descriptions of each of the settings (**Figure 5.2**).

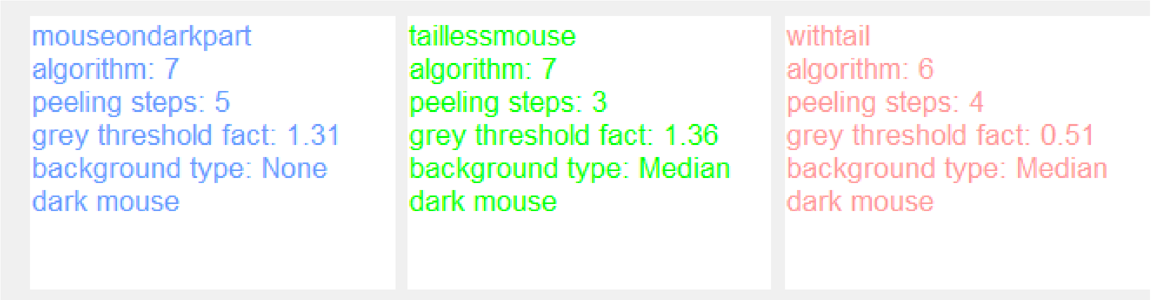

Figure 5.2. Display of setting details, generated when the DETAILS button is pressed. Font color corresponds to the setting color.

Zooming in on the image

The **ZOOM** button under the **USER COMMENT** panel allows zooming-in on the image. This will change the cursor to a cross allowing rectangle definition. Drag the vertex of the rectangle to define its dimensions. Once present, the rectangle can be moved over the image (by dragging it from the center) or resized (by dragging one of the edges or the vertices). When the rectangle is double left-clicked, the region within it will be shown. Rectangle definition uses the MATLAB *imrect* function. The **RESET** button will reset the zoom. **Figure 5.3** shows a zoomed-in section of the frame.

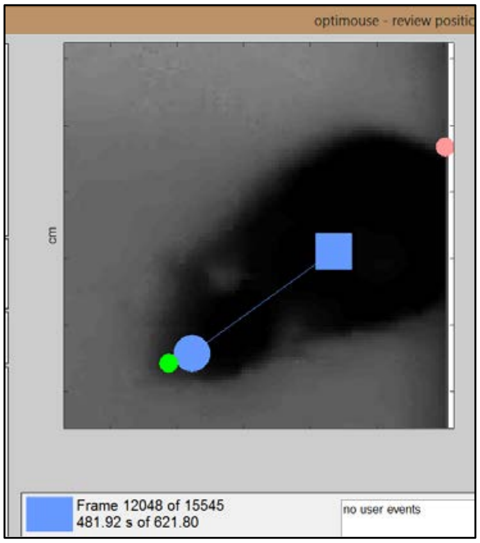

Figure 5.3. Zoomed view of mouse (frame 12048). Only part of the GUI is shown.

5.1.2. Frames without valid positions

Sometimes, a given setting does not yield a valid position for a particular frame. Technically, the position assigned by that setting (in that frame) is a MATLAB NaN (not a number). If it is the *active setting* that is not associated with a valid position, then the frame itself will not be associated with a position. **Section 5.6.2** describes how to identify frames without a valid position. Note that a valid setting is distinct from a correct setting.

5.2. Modifying settings in individual frames

5.2.1. Changing the active setting for a single frame

Any previously defined setting can be applied to the current frame by pressing the corresponding button in the **ASSIGN FRAME/SEGMENT** panel. The panel contains one button for each setting. The buttons' color and name identify the setting they are associated with.

For example, as seen in **Figure 5.1**, the green and blue settings result in correct nose detection in frame 12048, whereas the pink one does not.

In contrast, the default setting (blue) does not work well in frame 1577 (**Figure 5.4**). In this frame, only the second (green) and the third (pink) settings correctly identify the nose. Pressing the corresponding buttons will make the settings active. **Figure 5.5**, shows the same frame after applying the second (green) method.

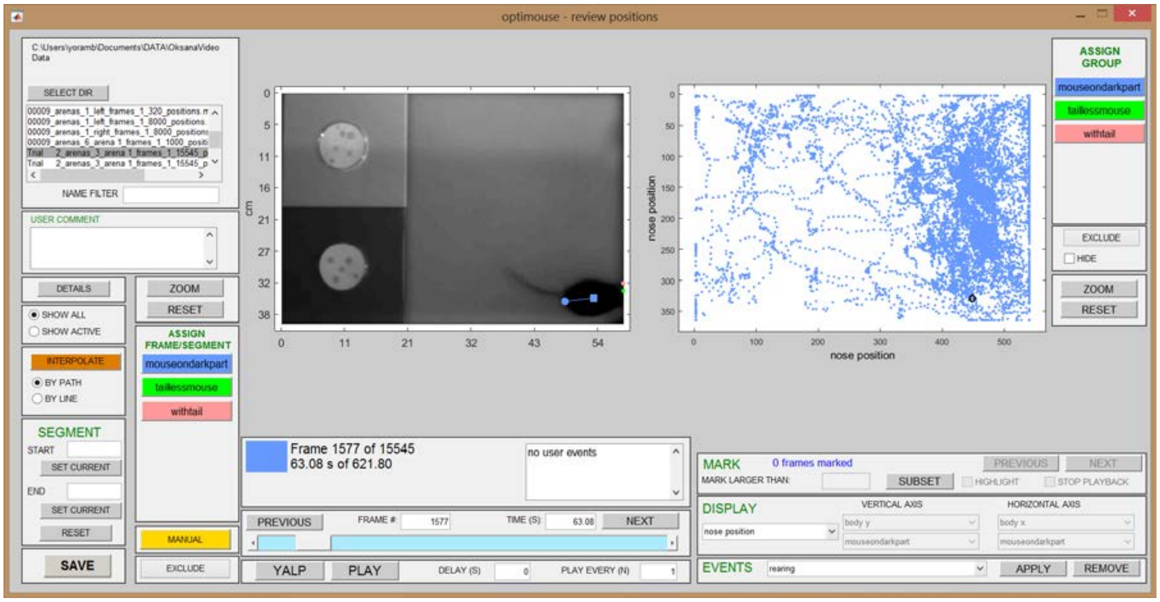

Figure 5.4. In this frame, the default setting fails to identify correct nose position.

Settings can also be applied from the keyboard. Pressing keys 1-6 will apply the corresponding settings to the current frame (if these settings were defined). For example, the second and third settings can be toggled with the **2** and **3** keys.

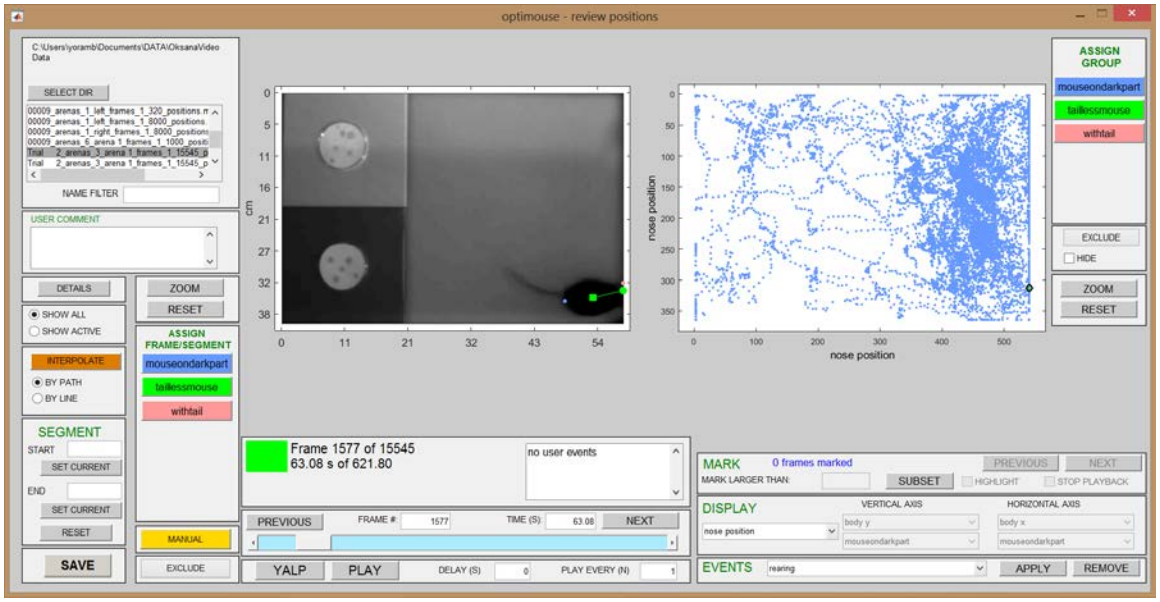

Figure 5.5. The second setting does correctly identify the nose position in this frame.

5.2.2. Setting a manual position

Sometimes, none of the settings provide *correct* detection in a given frame. In rare cases, none will yield a *valid* position (i.e. positions detected by all settings will be NaNs). OptiMouse provides several ways to handle such situations.

One option is to define positions manually, using the **MANUAL** button. After pressing the button, the cursor, when positioned over the frame image, will appear as a cross. The mouse position is specified as a line starting at the body center and ending at the nose. To define the line, left click the mouse, and while holding, drag to define the second endpoint and release. Once a line is drawn, it can be modified by dragging either of its ends. The position is set only after double clicking it.

The manually defined position automatically becomes the active setting for that frame. This is reflected by yellow the color of the square, circle and line connecting them. The manual position can always be overridden by the other settings. **Figure 5.6** shows a manually defined position.

If a manual position has already been defined for the current frame, but is not active, the **MANUAL** button will make it active. If the manually defined positioned is active, the **MANUAL** button will initiate a new definition, overriding the original. The definition of the manual position uses the MATLAB function *imline*.

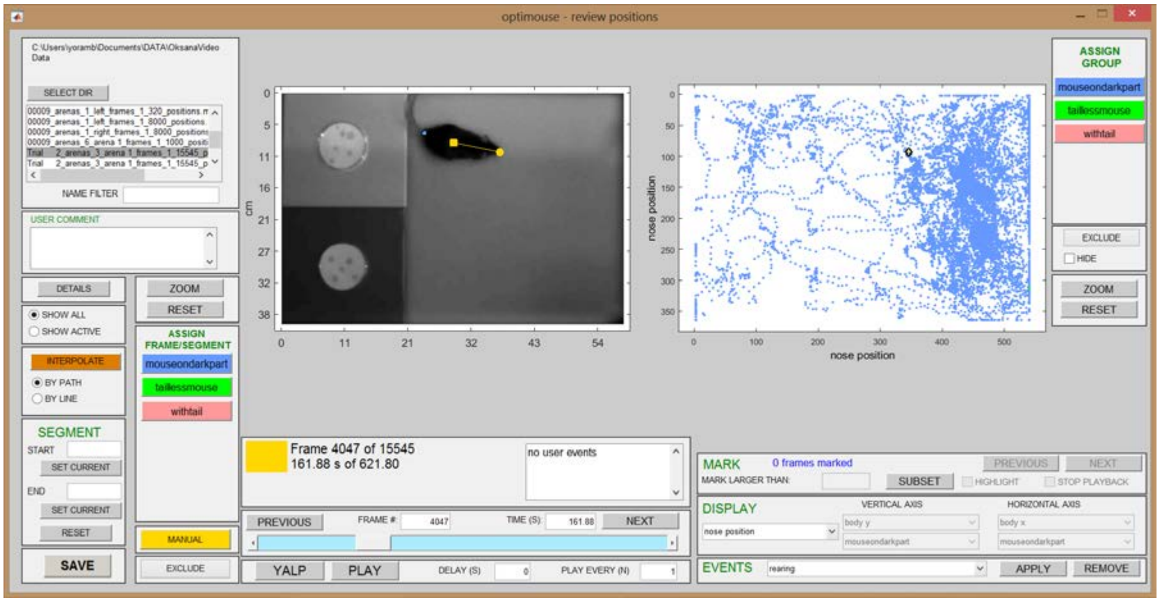

Figure 5.6. A frame after setting a manual position.

5.2.3. Excluding individual frames

Frames can be excluded from further analysis. When a frame is excluded, it is not associated with a position. The **EXCLUDE** button in the left side of the GUI excludes the current frame (the **x** key does the same thing). Excluded frames are identified by a gray rectangle below them and the absence of a line connecting nose and body coordinates. Frame exclusion can be reversed by applying any valid setting, or by defining a manual position. **Figure 5.7** shows an excluded frame. Positions in explicitly excluded frames, as in frames without valid active settings, are also designated as NaNs, and are ignored subsequent analyses.

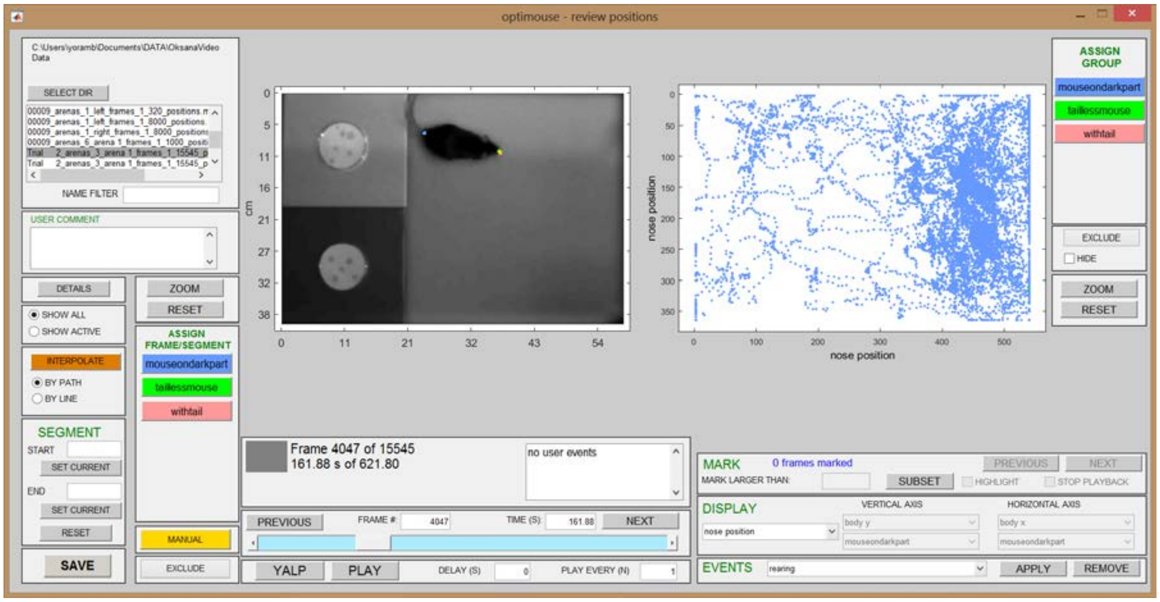

Figure 5.7. **The same frame shown in Figure 5.6, after excluding it. Note the gray square below the image, and the lack of a connecting line.**

5.3. Operations on entire segments

5.3.1. Applying settings to segments

Applying settings to each individual frame is time consuming and not practical for entire sessions. OptimMouse provides several methods to allocate settings to multiple frames. One method is to apply a setting to a continuous range of frames - a *segment*. A *segment* is defined by *start* and *end* frames, set using the **START** and **END** edit boxes in the **SEGMENT** panel. Alternatively, pressing the **SET CURRENT** buttons, located below the edit boxes, will assign the current frame as either the *start* or *end*. Segment *start* and *end* can also be set from the keyboard using the **s**, and **e** keys, respectively.

When a valid segment is set, the buttons for each of the settings (in the **ASSIGN FRAME/SEGMENT** panel) will apply the setting to *all* frames in the segment (this is also true for the keyboard shortcuts associated with these settings). Similarly, pressing the **EXCLUDE** button when a segment is defined, excludes all frames within the segment.

Manual positions cannot be set for a segment, but as described in **Section 5.3.2** below, positions in a segment can be *interpolated* based on the *start* and *end* frames.

5.3.2. Interpolating positions in a segment of frames

It may be that an entire sequence of frames will not be correctly detected with any predefined settings. Setting manual positions to each frame is impractical. To address such cases, OptiMouse provides an option for *interpolation*. When a valid segment is defined, as described above, the **INTERPOLATE** button interpolates positions in all frames between *start* and *end* frames. Interpolation can be performed from the keyboard using the **i** key.

Body and nose positions at the *start* and *end* frames serve as anchors for interpolation, and are not modified. Thus, to serve as anchors for interpolation, the *start* and *end* frames must have valid (non NaN) positions associated with their active setting.
Interpolated frames are automatically assigned to the interpolated class which are shown in ochre. When one of these frames is selected, an **INTERPOLATED** button appears in the **ASSIGN FRAMES/SEGMENT**. Interpolation is reversible, and other settings can always be applied as described above (and also below).

OptiMouse provides two interpolation algorithms, determined using the two radiobuttons under the **INTERPOLATE** button.

5.3.2.1. Interpolation by PATH

The default interpolation option is **BY PATH**. Here, vectors defined by the mouse angle and length are taken from the **start** and **end** frames. The angles and lengths of these vectors are then linearly interpolated for all the other frames within the segment. Each of these vectors is then applied to each frame, with its origin placed at the body center.

5.3.2.2. Interpolation by LINE

In the **BY LINE** interpolation algorithm, body and nose positions are each linearly interpolated, using the *start* and *end* frame positions. Thus, the paths of both body and nose positions will define a straight line from *start* to *end* frames.

The conditions that determine which algorithm is more suitable are complex. Practically, both algorithms must be tested to determine which provides better results. Generally, interpolation only works well for short segments with gradual monotonic changes. For example, if the mouse angle and length change in a complex, non-monotonic way, the **BY PATH** approach will not work well. The **BY LINE** algorithm will only work if the mouse trajectory within the segment defines a straight line at a constant speed. Applying interpolation in cases where the requirement of linearity are violated can lead to very misleading outcomes. *Whichever algorithm is used, the results of interpolation must be monitored.*

Figure 5.8 shows the same frame as in **Figure 5.6** and **5.7**, with interpolated positions (rather than manual position).

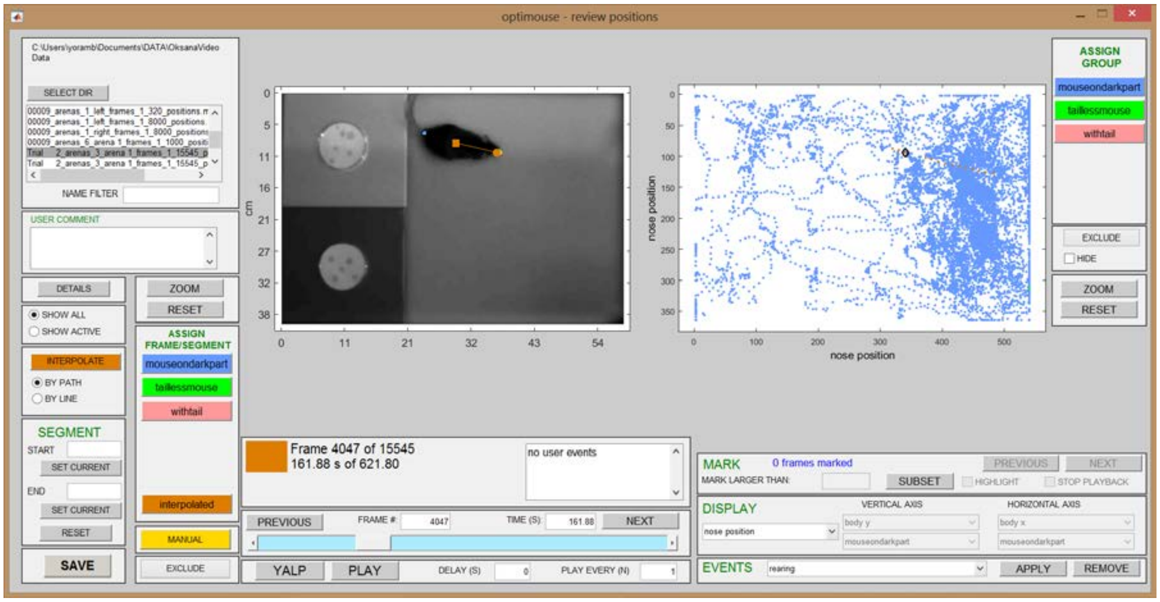

Figure 5.8 The same frame shown in Figure 5.6, and 5.7, after interpolating frames. In this example, frames 4044 and 4048 were used as anchors and the BY PATH option was used for interpolating positions. The previously defined manual position is still stored, but is not active. Interpolated positions are indicated in ochre.

5.4. Annotating frames

OptiMouse provides basic annotation with user defined events.

Annotation requires an initial definition of character strings that can be used as annotation events. The definitions are made in the file *user_annotation_events.m* in the *optimouse_user_definitions* folder. See **Appendix 7** for a description of this file and how to define events.

The list of events is read from the file and used to populate the pull down menu in the **EVENTS** panel (on the bottom right side of the **Review GUI**). It is advisable to assign short event names, so they will not be truncated in the **Review GUI**.

Events in the list can be assigned to selected frames using the buttons in the **EVENTS** panel. The **APPLY** button adds the selected event (from the pull down menu) to the current frame, or segment (if a valid segment is defined). The **REMOVE** button *removes* the selected event from the current frame, or segment. A frame can be associated with multiple events. The **APPLY** and **REMOVE** buttons can be activated from the keyboard using the **a** and **r** keys, respectively. **Figure 5.9** shows an annotated frame.

Events associated with the current frame are indicated in the text box below the frame image. A procedure for identifying all annotated frames within the session is described in **Section 5.6.2**.

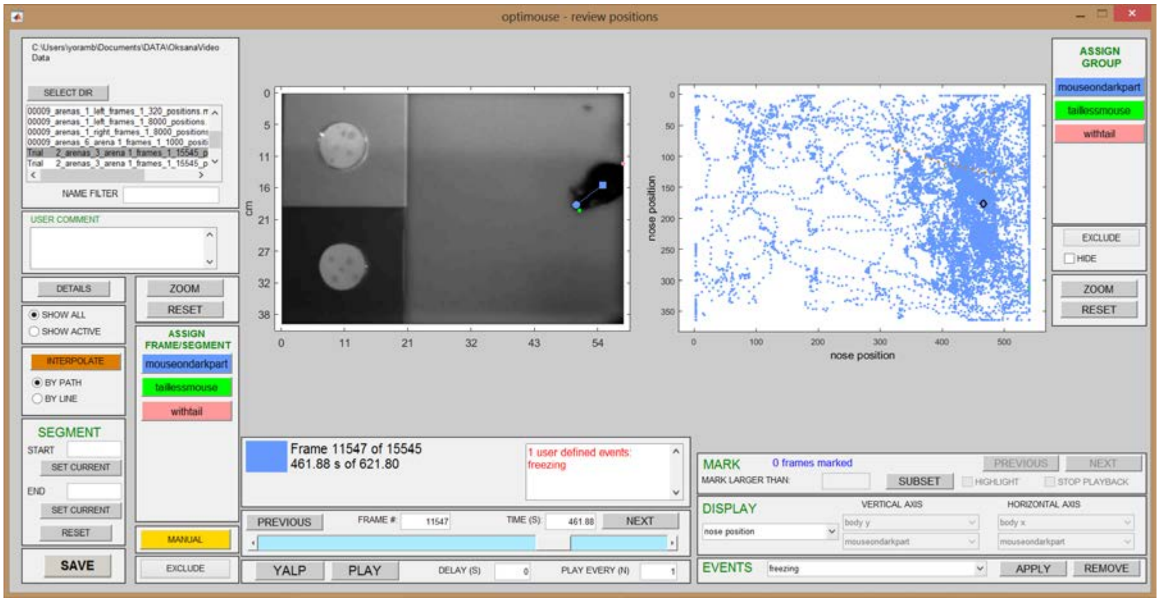

Figure 5.9 **A frame assigned with the event *freezing* as indicated by the red text below the mouse image. The event was defined in the user_annotation_events.m file. It was added to the frame by pressing the APPLY button when it was selected in the EVENT panel listbox. A frame can be associated with multiple events.**

5.5. Combining navigation tools and keyboard shortcuts to view and correct positions

The tools described thus far allow browsing the session to monitor, and if necessary, to correct, erroneous detections in individual frames or segments. Below is a suggested procedure for achieving this goal:

1. View the movie using the **PLAY/PAUSE** button and/or by advancing individual frames using the **NEXT** and **PREVIOUS** buttons. This can be done more efficiently with keyboard shortcuts (>,<, for individual frames, and ALT>,ALT< for playback).
2. Whenever a frame that requires correction is found, pause the movie.
3. In each such frame, move one or two frames back in time using the **<** key, identify the first frame that requires correction, mark it as a segment start (using the keyboard shortcut, **s**). Then move forward using the **>** key (one frame) or **shift >** (an entire second) to find the last frame that requires correction. Mark it as a segment end (using the keyboard shortcut, **e**).
4. If a frame, or an entire segment, requires correction, do one of the following:
   - **4.1.** If a predefined setting is suitable for all frames in the segment, apply it using the corresponding keyboard shortcuts (1,2,3,4,5,6).
   - **4.2.** If there is no suitable predefined setting, but the frames do include a mouse with a defined position, then
     - **4.2.1.** Interpolate the frames between segment start and segment end using the keyboard shortcut (**i**), or

4.2.2. Define manual positions for the frames. More efficiently, manual positions can be defined for a few frames, and these can then serve as anchors, allowing to interpolate positions in others frames.

4.3. If frames do not include a valid mouse image, exclude them using the keyboard shortcut (**x**).

Frames can be efficiently annotated by combining movie navigation shortcuts, segment definitions shortcuts and annotation shortcuts.

Completion of this procedure will result in correct position detection in the entire movie. However, it may be very time consuming, particularly for long movies requiring many corrections. The other set of controls in the **Review GUI,** described below, is designed to facilitate the reviewing and correcting process.

5.6. The Parameter View

The right side of the **Review GUI**, which we refer to as the *parameter view*, allows viewing parameters associated with each frame, and reciprocally, *accessing* frames with particular parameter values. This display and the associated controls are designed to facilitate the identification and correction of frames with erroneous position detection. We first describe the various displays and controls, and then, explain how they can be applied to facilitate correct position detection.

In the parameter view, each frame is represented by one dot. The position of the dot is determined by the frame's attributes (see below). Its color is determined by the setting associated with it, following the color scheme described above.

When a session is initially selected, the *nose position* view is shown. In this view (shown in all previous figures of the **Review GUI**), dot positions represent the nose position according to the *active setting*. Thus, initially, all dots will be blue (the color associated with the first, default setting).

The dot representing the current frame is surrounded by a black diamond. As different frames are selected, and when the movie is played, the diamond changes position.

In the *nose position* view, frames that do not have a valid position will not be shown as dots. Note the absence of a diamond in **Figure 5.7**, and its presence in **Figures 5.5** and **5.6**.

5.6.1. Accessing frames according to particular attributes and Zooming-In

Clicking near one of the dots will show the frame corresponding to the selected dot. Note that it is necessary to click *near* rather than *on* a dot. Thus, selecting a dot within a very dense cluster may be difficult and requires zooming-in. This is done with the **ZOOM** and **RESET** buttons on the right side of the **REVIEW GUI**, using the same procedures described for the frame image zoom.

5.6.2. The DISPLAY panel

This panel contains five pull-down menus which determine which parameters to shown in the *parameter view*. The menu on the left is at the top of the hierarchy. The other menus become active only when the *parameter pairs* option is selected from this menu (see **Section 5.6.3**). We demonstrate the different views with an example of a session with four different settings

Nose position (**Figure 5.10**)

Dots indicate the position of the nose, as determined by the active setting for each frame.

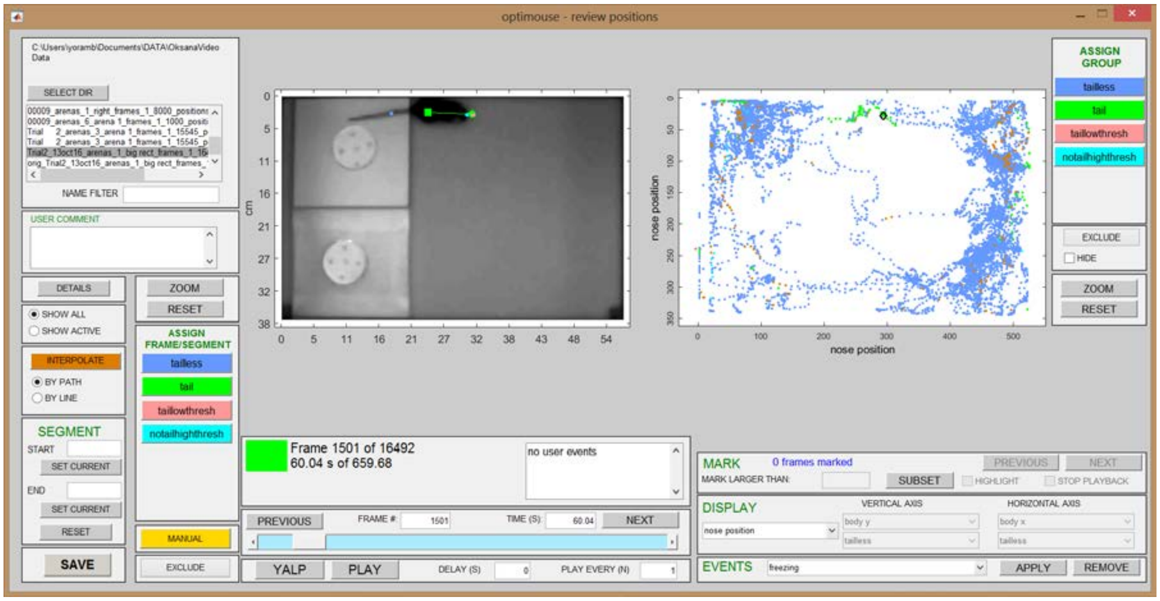

Figure 5.10 The nose position view

Body position (**Figure 5.11**)

Dots indicate the position of the body (mouse center of mass), as determined by the active setting for each frame.

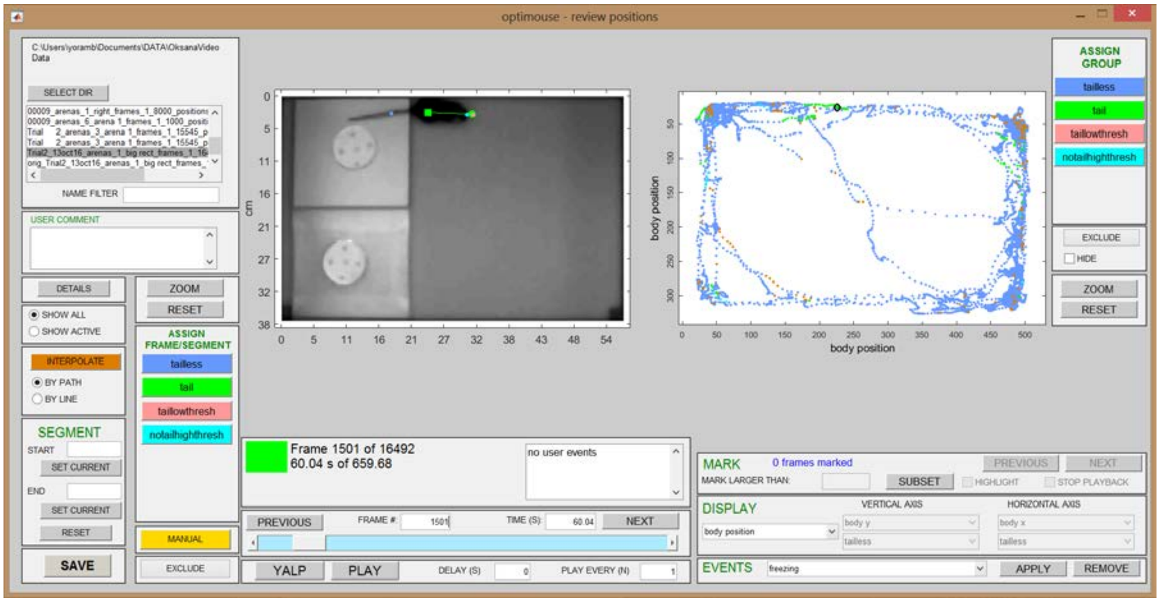

Figure 5.11 The body position view

Active setting (Figure 5.12)

Dots indicate the setting associated with each frame (frame number appears on the horizontal axis). In addition to each of the user defined settings (indicated numerically in the vertical axis as 1, 2, 3, 4, 5, 6) there are entries for excluded frames (denoted as X in the vertical label), manually-set positions (denoted as UD), and interpolated frames (INT). The NaN category denotes frames without a valid position. The values in parentheses on the vertical axis indicate the number of frames associated with each category. Clicking near dots in the NaN category will show those frames for which the active setting (indicated by the dot color) does not provide a valid position. As described above, such frames may well have a valid position using other settings, and if not, manual positions can be defined. **Sections 5.8 and 5.9** describe how all NaN frames can be accessed efficiently.

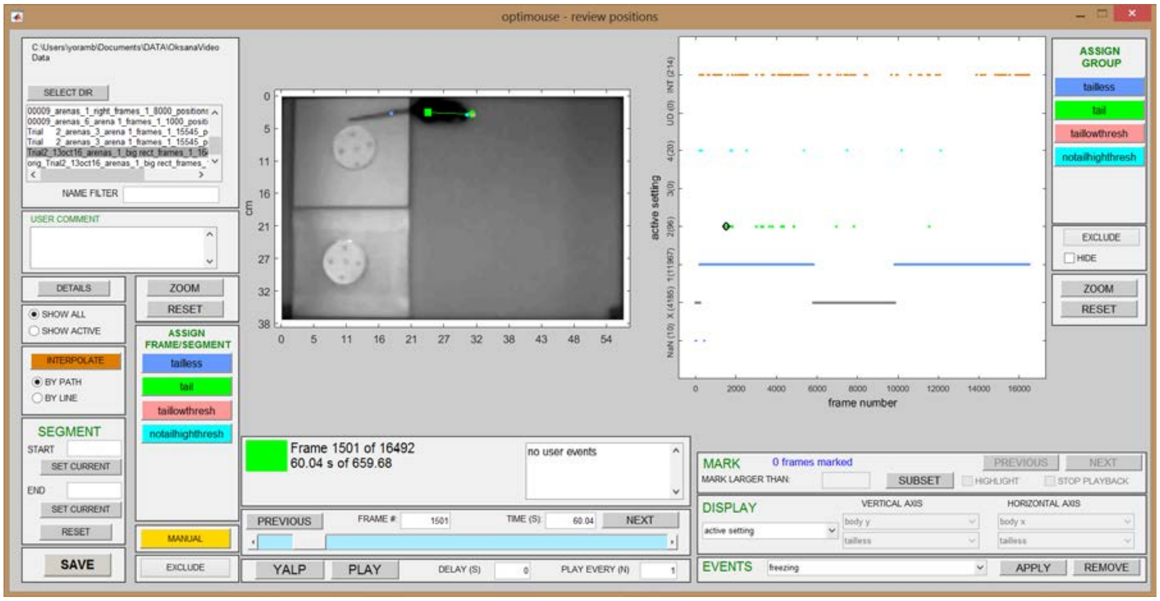

Figure 5.12 The active setting view

Angle Change (Figure 5.13)

Dots indicate the absolute difference, in degrees, between mouse angles on consecutive frames. The value for any given frame is the *absolute* value of the angle difference between it and the one proceeding it. This view is useful for identifying frames with incorrect positions: mice do not make 180° body flips within a fraction of a second, but a very common detection error is to confound nose and tail positions, which can lead to such abrupt changes between consecutive frames if detection is correct in one, and incorrect in another.

Thus, selecting dots corresponding to large changes will usually reveal incorrect detection on either the frame itself or the one preceding it. On the other hand, small angle change values do not necessarily imply correct detections (an entire sequence of frames may have similar incorrect detections, resulting in small angle changes between them).

When the angle change view is selected, three vertical lines appear in the display. Their significance and purpose are explained in **Sections 5.8 and 5.9** on the **MARK** and **CORRECT** panels. These panels are designed to facilitate viewing and correcting such fast angle changes. The **CORRECT** panel appears only when the angle change view is selected.

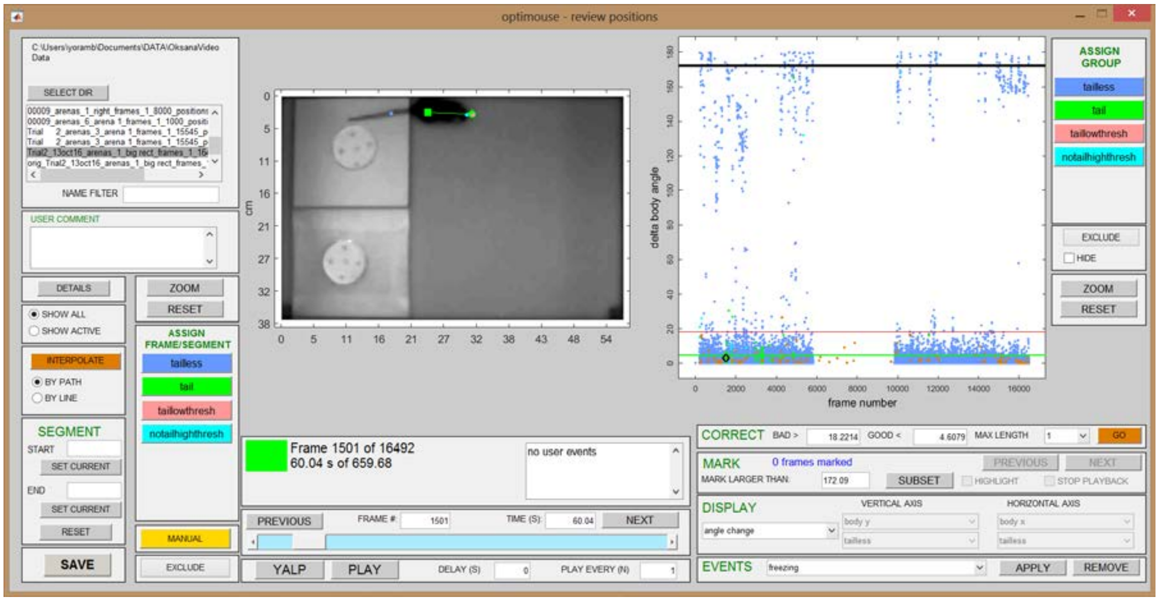

Figure 5.13 The angle change view

Mouse angle (Figure 5.14)

Dots represent the mouse angle as a function of frame number. Angles are defined between 0° and 360°. Note that this view is not very useful for detecting abrupt angle changes, since changes between e.g. 1° and 359° may appear large on the display, while they are actually small.

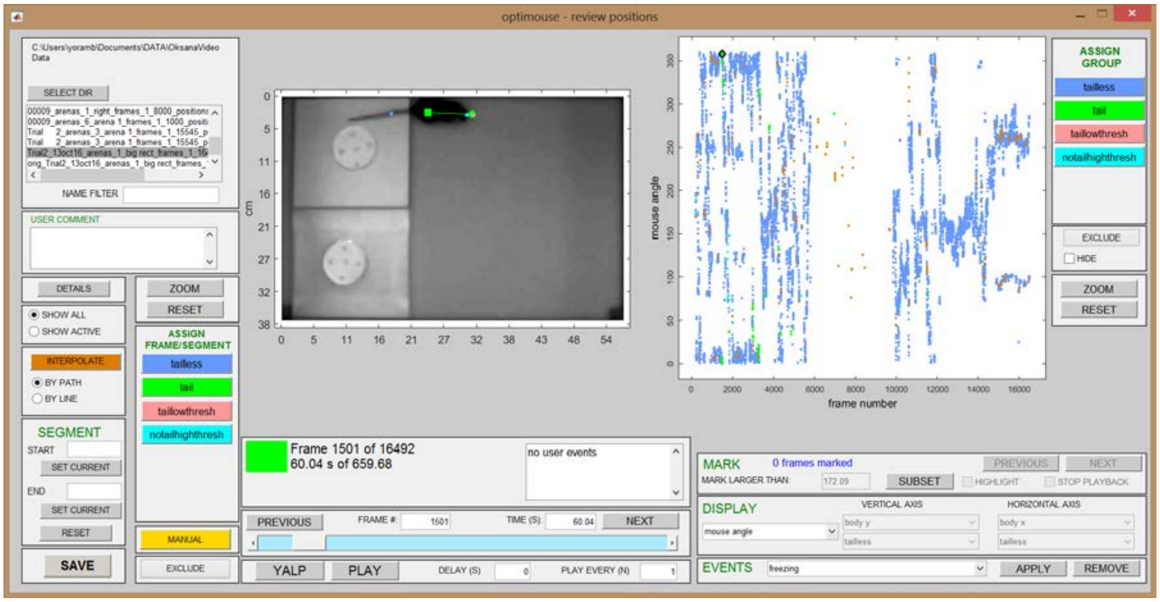

Figure 5.14 The mouse angle view

Body Speed (Figure 5.15):

Dots represent the speed of the mouse as a function of frame number. The value in any given frame represents the speed from the preceding frame into the current frame. This view includes a horizontal black line whose significance is explained in **Sections 5.8** and **5.9** on the **MARK** panel.

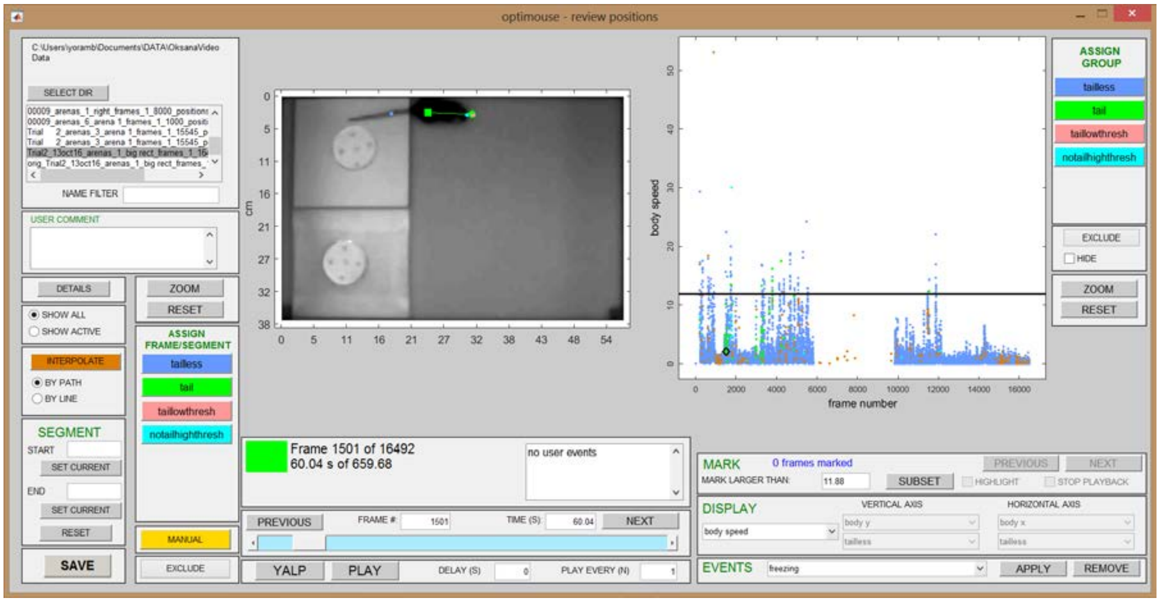

Figure 5.15 The body speed view

Nose speed (**Figure 5.16**)

Dots indicate the speed of the nose relative to the body (as a function of frame number). This measure is highly correlated with, but not identical to, the angle change parameter. As with *angle change* and *body speed* views, the value represents the speed going into the current frame. Extremely high values are usually due to erroneous detections, in either the current, or the preceding frame. This view also includes a horizontal black line described in **Sections 5.8** and **5.9**.

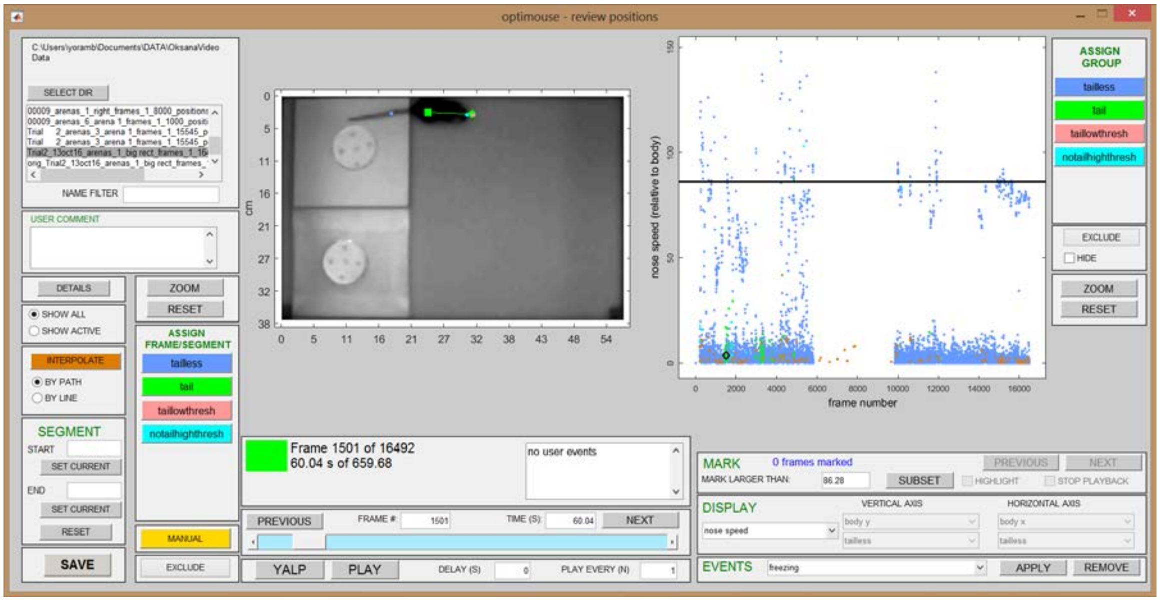

Figure 5.16 The nose speed view

Annotated events (**Figure 5.17**)
In this view, the dots indicate, for each frame, whether an event has been assigned to it. Although the identity of the event is not indicated in this display, clicking near a dot in the upper row will reveal the frame and all events associated with it.

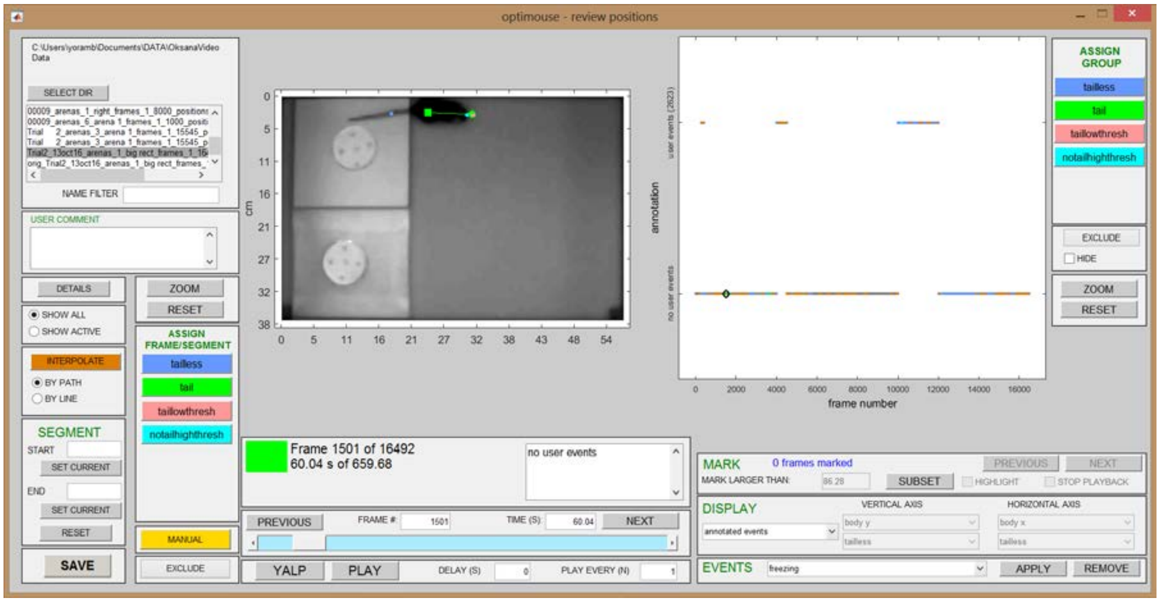

Figure 5.17 The annotated events view

5.6.3. Parameter Pairs

Selecting the *parameter pairs* option activates the other pulldown menus in the **DISPLAY** panel (they are inactive in **Figures 5.10-5.17**). Using these menus, a variety of parameter pairs can be viewed on the vertical and horizontal axes. For each axis, it is required to select which parameter to show using the *upper* pull down menus. The *lower* pull down menus specify the setting from which the parameter value is derived. Note that here, the dot positions do not necessarily represent values associated with the active setting. Their color however, does show the active setting. Axes labels assume the color of the setting that they represent. The ability to select the settings and the parameters for each axis provides many options for identifying frames with particular properties.

5.6.3.1. Viewing the same parameter as detected by different settings

To provide one example, it is possible to show mouse angles determined by the one setting *vs.* those determined by another setting. The dot selected in **Figure 5.18** is on the diagonal, indicating that similar angles were determined by both settings. Selecting an off-diagonal dot, corresponding to a ~180° difference, will reveal frames in which at least one of the settings yields a wrong nose position.

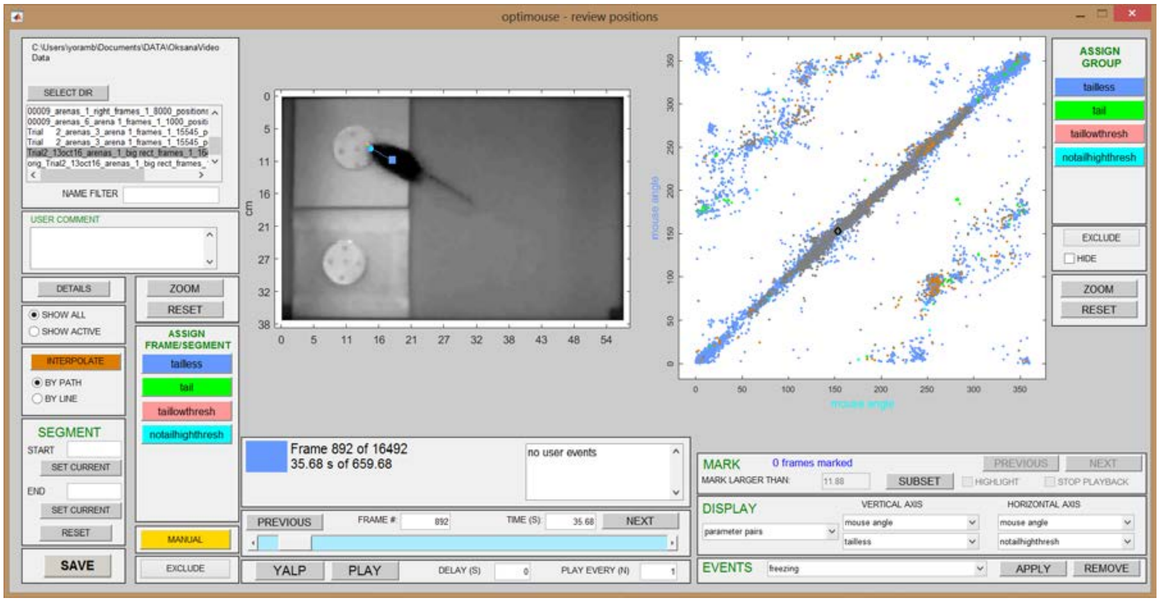

Figure 5.18 Parameter pairs view showing mouse angle as detected by different methods

5.6.3.2. Viewing two different parameters from the same setting

Another possibility is to view the relationship between two parameters from the same setting. For example, **Figure 5.19** shows mouse length and mouse area as determined by the first setting. This is an example where outlying dots, like the one selected in **Figure 5.19**, can reveal problematic frames that should be excluded (see **Section 5.7.2**). The example in **Figure 5.19** is clearly extreme, but more subtle problems can also be revealed outlying dots.

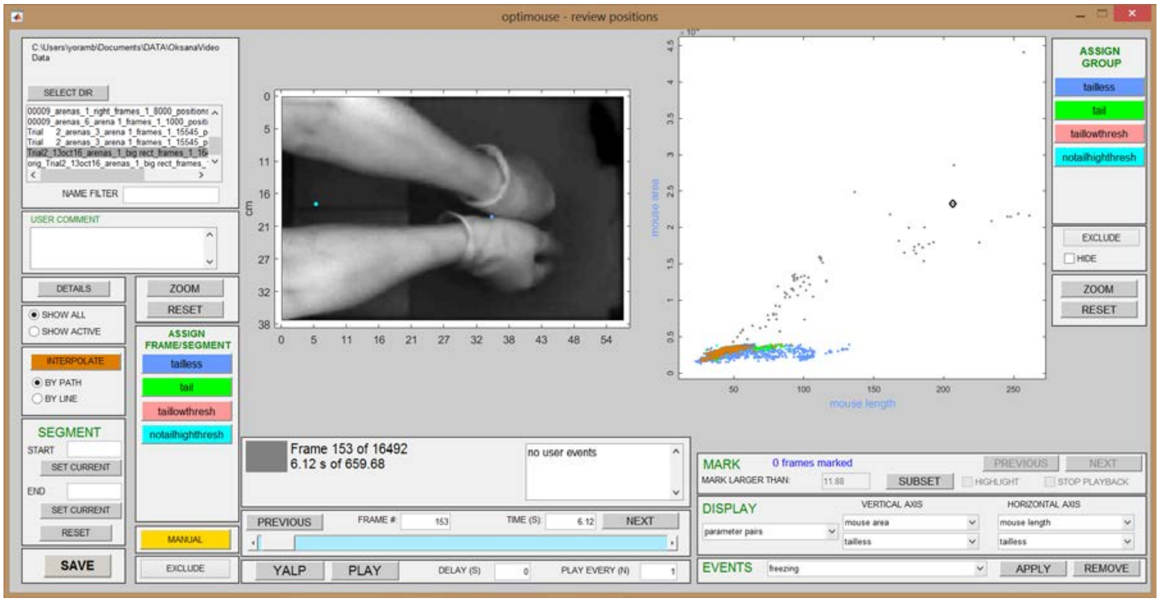

Figure 5.19 Parameter pairs view showing mouse area *vs.* mouse length for a single setting.

The number of possible parameter combinations is very large and it is definitely not intended to examine each of them. The rationale behind the parameter display is that some parameters may characterize frames with important attributes. Different parameters may be useful for different sessions and for different analyses.

5.6.3.3. A description of all parameter pairs

Below is a description of all parameters.

Parameters related to mouse position

body x: horizontal position of the mouse center of mass.

body y: vertical position of the mouse center of mass.

nose x: horizontal position of the mouse nose.

nose y: vertical position of the mouse nose.

Note that the vertical position (as defined by the video image display) can be plotted on the horizontal axis of the parameter view, and vice-versa.

mouse angle: the angle of the line connecting the nose and the body center of mass.

Parameters related to mouse shape

trim factor: ratio between the length of the mouse before trimming to the length after trimming. This indicates whether the tail of the mouse has been properly identified. Values will typically be large if the tail is detected and is in the same orientation as the body.

mouse perimeter: perimeter of the mouse before peeling.

thinned mouse perimeter: perimeter of the mouse after peeling.

perimeter ratio: ratio of the perimeters before and after peeling.

Parameters related to mouse dimensions

mouse area: total number of pixels associated with the detected object.

mouse length: distance between the body center of mass and the nose.

Parameters associated with image intensity

grey threshold: software determined threshold values for the frame.

mouse intensity mean: mean intensity of mouse pixels.

mouse intensity var: variance of the intensity of mouse pixels.

mouse intensity range: difference between the minimal and maximal intensity pixels of the mouse.

background intensity (mean): mean intensity of the rectangle containing the mouse object without mouse pixels.

Frame number
Allows viewing how another parameter changes as a function of frame number.

5.7. Applying settings to frames based on common attributes
The ability to mark, and change settings of frames using polygons is especially useful when frames with similar attributes form clusters under particular parameter combinations. For example, when the mouse is in certain parts of the arena with a distinct background color, one particular setting may be preferable over others. Such frames can be identified using the background color parameter. Or, when the tail is hidden, there may also be one particular optimal setting. Such frames can be identified using the mouse length or perimeter. To give a third example, when a mouse is moving with an elongated body, there may also be one ideal setting. Such frames can be identified using the length and speed parameters. The parameter view allows applying a setting to a group of frames whose dots cluster in the parameter view. This is accomplished by surrounding selected dots with a polygon, a procedure that technically is identical to the definition of polygon shaped arenas.

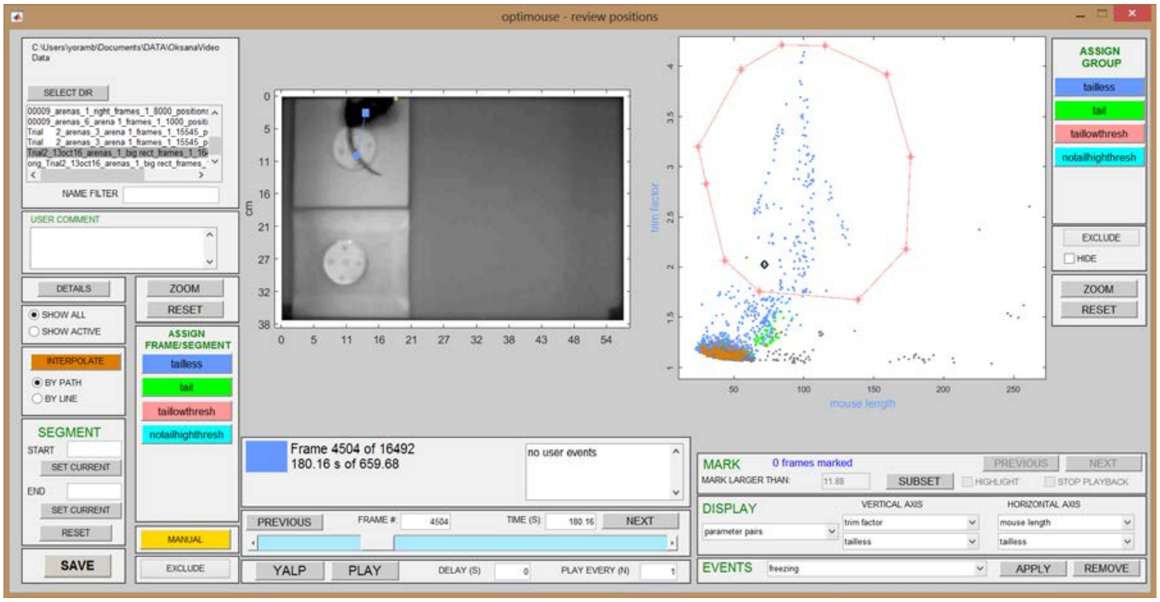

Figure 5.20 **Application of a setting to a group of frames using the ASSIGN GROUP panel. To complete definition it is still required to double click the polygon, as shown in the next figure.**

5.7.1. Applying settings to frames using the parameter view
The **ASSIGN GROUP** panel on the right side of the **REVIEW GUI** includes buttons for each of the settings. When one of these buttons is pressed, the cursor changes to a cross hair, and allows defining a polygon on the parameter view. The procedure for defining polygons is identical to that described in **Section 3.3.2.2**. Once definition is complete, all the points within the polygon are assigned with the button's setting. **Figures 5.20** and **5.21** illustrate the application of the third

setting (pink button) to a group of frames in which the default setting fails. These frames are identified by characteristic trim factor and length values. **Figure 5.20** and **Figure 5.21** show the definition before and after the polygon was double clicked, respectively. Note the change in the current frame image following the definition.

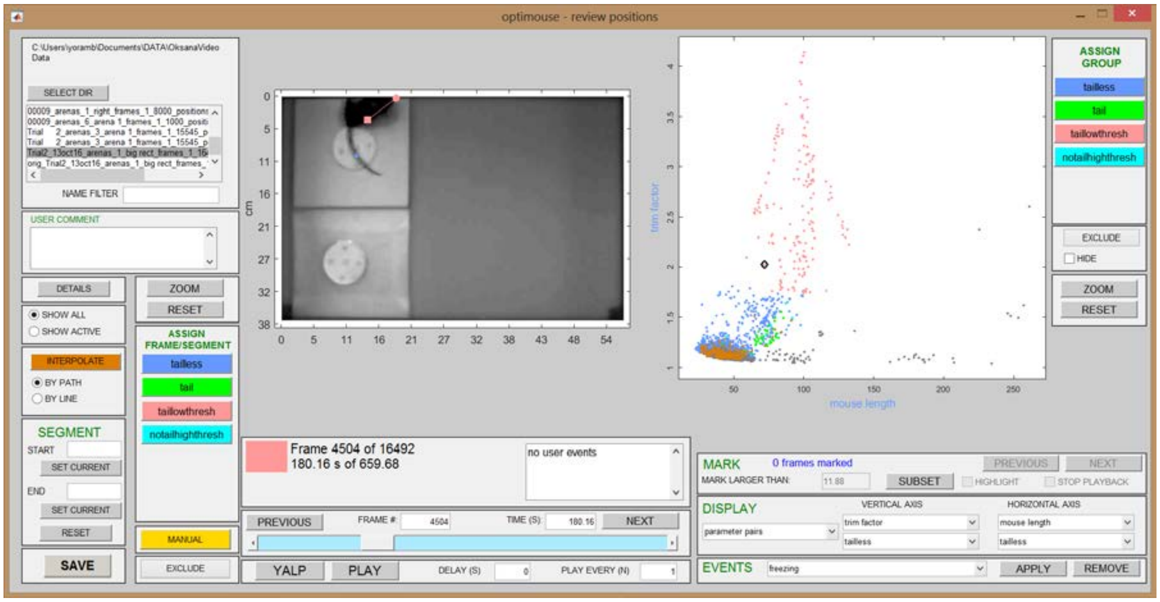

Figure 5.21 After double clicking the polygon shown in Figure 5.20, all frames corresponding to dots inside it are associated with the third (pink) setting.

5.7.2. Excluding frames using the parameter view

Polygon definitions can also be applied to exclude a group of frames using the **EXCLUDE** button on the right side of the **Review GUI.** After drawing a polygon around the dots designated for exclusion, they will be shown in gray (**Figure 5.22**). Excluded frames will not be shown in the parameter view if the **HIDE** checkbox under the **EXCLUDE** button is checked. This is useful when dots corresponding to the excluded frames impair visibility of relevant frames. Note that even when frames are hidden in the parameter view, they are still shown in the video display.

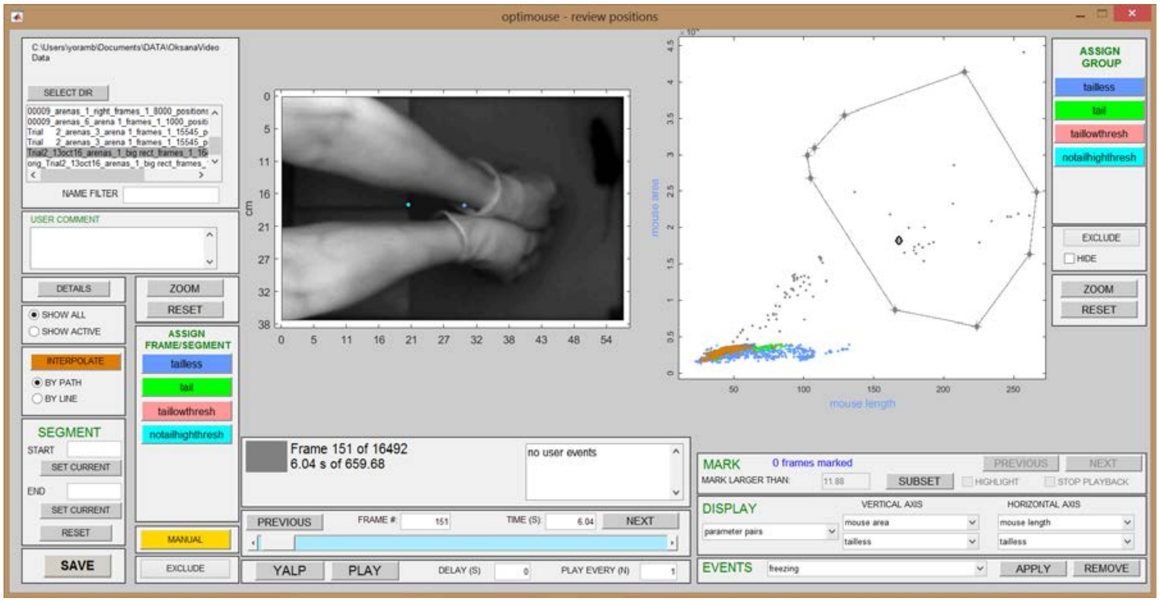

Figure 5.22 **excluding a group of frames using the EXCLUDE button. To complete exclusion definition it is still required to double click the polygon.**

5.8. Marking and focusing on a subset of frames

Controls in the **MARK** panel allow highlighting frames with particular parameter values for closer inspection. Frames can be marked in one of two ways.

5.8.1. Marking frames using the SUBSET button

The **SUBSET** button allows marking frames using a polygon on the parameter view. The number of marked frames and the percentage that they constitute of the total will be indicated. Once a subset has been defined, the other controls within the panel, described below, become active.

5.8.2. Marking frames according to large parameter values

The second way to define a subset is only applicable to the *angle change*, *body speed*, or *nose speed* views. Parameters associated with these views are unique because large values are often indicative of incorrect detection (see also **Section 5.9**). When one of these views is selected, a black horizontal line is shown. The height of the line corresponds to the 99th percentile of the parameter value distribution and is indicated in the **MARK LARGER THAN** edit box. To modify the height of the black line (and the subset of marked frames), enter the desired value into the **MARK LARGER THAN** edit box, and press the *enter* key. As a result, all frames with values exceeding the specified value will be marked, overwriting any previously marked group of frames. **Figure 5.23** shows the **Review GUI** after frames associated with high nose speed were marked. All marked frames are shown as larger dots, and the number and percentage of marked frames is indicated in the **MARK** panel.

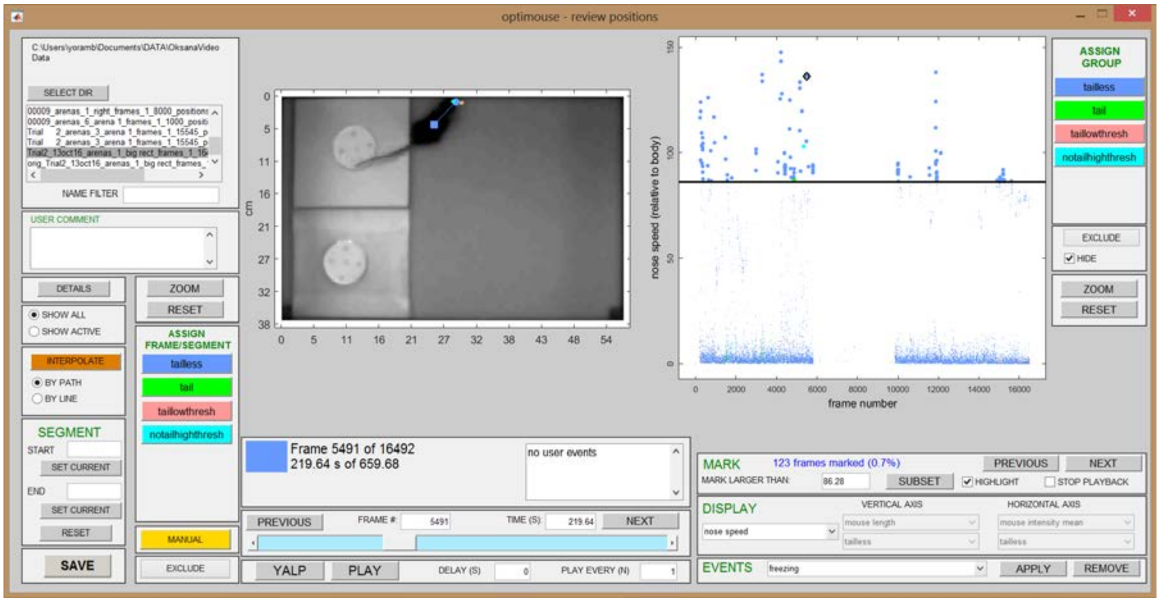

Figure 5.23 **Marking frames with high nose speed values. Frames were marked by entering the value in the MARK LARGER THAN edit box.**

5.8.3. Examining marked frames

Selecting the **HIGHLIGHT** checkbox shows all marked frames as large dots, and all unmarked frames as small dots. This checkbox is automatically selected when a subset is defined. When marked frames are highlighted, it is possible to identify frames marked in one view, under another view. This is illustrated in **Figure 5.24** which shows the nose position view with frames marked in a different view.

Regardless of which method was used to mark a subset of frames, the **PREVIOUS** and **NEXT** buttons within the panel (not to be confused with those in the main navigation panel) will move to the previous and next frames *in the marked subset,* skipping unmarked frames. When the control (Ctrl) key is held, the **<** and **>** keys also advance to the previous and next marked frames.

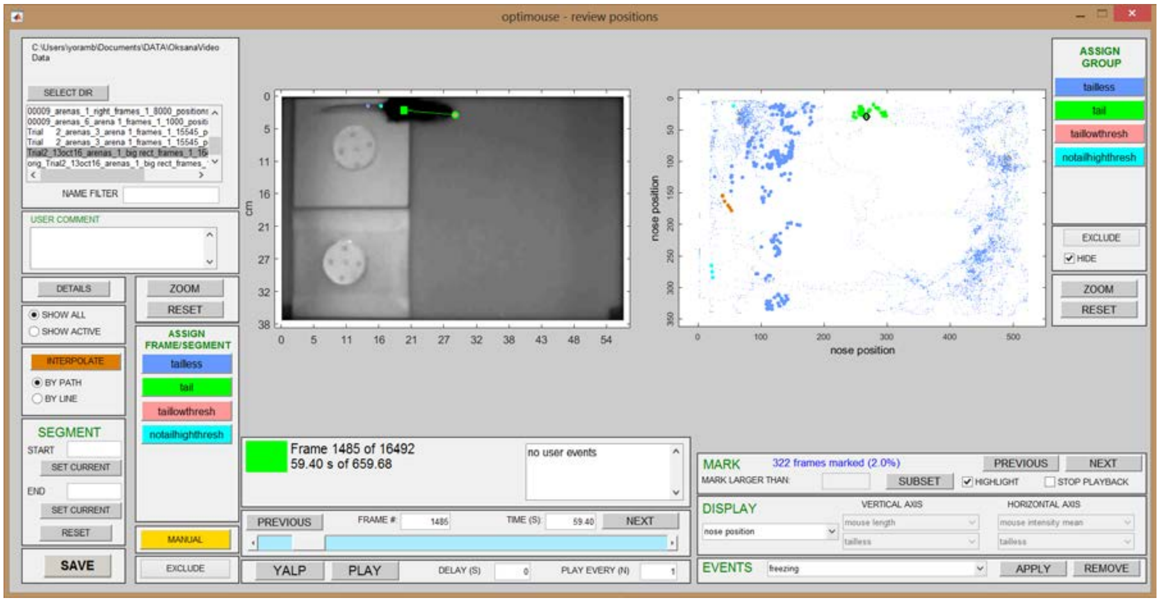

Figure 5.24 **Frames were marked according to mouse length using the SUBSET button. The view was then changed to nose position. Note that frames with longer mouse lengths tend to occupy particular arena locations. Note also that the number of marked frames is indicated (322, 2%).**

When the **STOP PLAYBACK** checkbox in the **MARK** panel is selected, continuous playback (using the **PLAY** or **YALP** buttons), automatically pauses when a frame in the marked subset is reached. This allows selectively stopping playback on frames requiring closer inspection.

5.8.4. Focusing on frames in particular arena locations

One use of the **MARK** panel is to focus on frames in particular arena locations. For example, if the arena includes an odor source, accurate detection of nose positions away from the source may be unnecessary. By combining the *body position* view with the **SUBSET** button, it is possible to mark frames in which the mouse is located near some region of interest. Once marked, frames can be accessed using the **MARK** panel controls. By limiting in-depth examination to a subset of important frames, the reviewing process becomes much more efficient. **Figure 5.25** shows the GUI after marking all frames near the lower odor source.

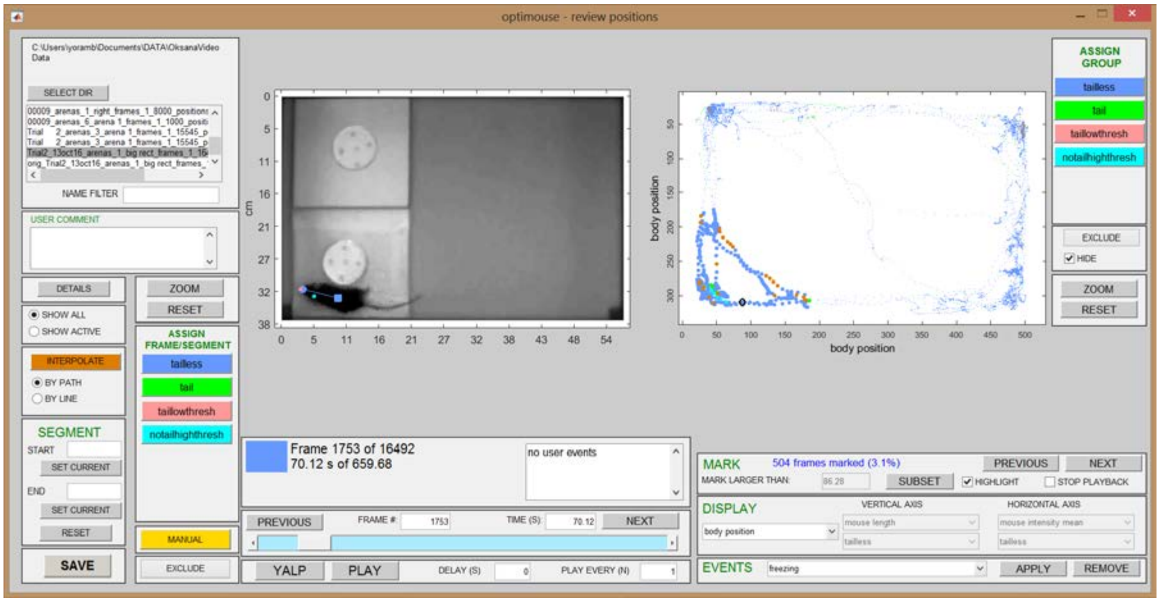

Figure 5.25 The Review GUI after marking frames in which the mouse occupied the lower left part of the arena.

5.9. Correcting brief detection failures using the CORRECT panel

The **CORRECT** panel is designed to identify, and correct, brief sequences of wrong detections.

5.9.1. The concept of transient error correction

Erroneous detections are typically characterized by fast angle changes between consecutive frames (see *angle change* in **Section 5.6.2**). If a small number of such “reversed” detections occur within a sequence of correct detections, this will result in brief transients in calculated angle. To illustrate, consider a series of six frames in which the actual mouse direction is 90° (up, in **Figure 5.26**). If detection is correct in all frames, angles are detected as [90°,90°,90°,90°,90°,90°], and the *angle change* between consecutive frames will be [0°,0°,0°,0°,0°] (**Figure 5.26A**). If a reverse (incorrect) detection occurs in the third frame, the sequence of detected angles will be [90°,90°,270°,90°,90°,90°], with the corresponding angle changes: [0°,180°,180°,0°,0°] (**Figure 5.26B**). Note that changes are specified in absolute values, as the direction of change is irrelevant in this context.

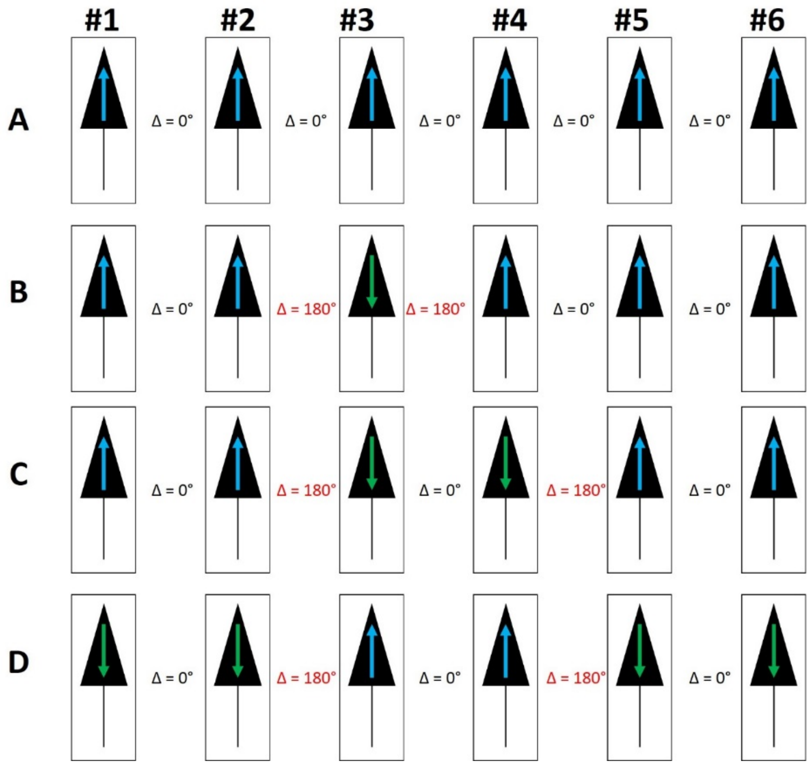

Figure 5.26 **Four scenarios (A-D) illustrating the concept of correcting brief detection errors. Each scenario includes a sequence of six frames. Values between frames indicate the change in detected angles between them. The different scenarios are explained in the text.**

A similar scenario is a sequence of two consecutive reverse detections. Using the same example, a detected sequence of [90°,90°,270°,270°,90°,90°], will result in the following sequence of changes: [0°,180°,0°,180°,0°] (**Figure 5.26C**) which can also indicate a transient failure.

We stress that such sequences of angle change can only detect abrupt transitions, but provide no information regarding whether the fast transitions, or the flanking frames, represent wrong detections. For example, if the sequence of *real* mouse angles is [90°,90°,90°,90°,90°,90°], but is incorrectly detected as [270°,270°,90°,90°,270°,270°], this will also appear as a fast transition (**Figure 5.26D**). If "corrected", it will be altered to [270°,270°,270°,270°,270°,270°], which is obviously wrong. ***The crucial point is that automatic correction works only if incorrect detections occur in a minority of frames, over a dominant background of correct detections.***

5.9.2. Application of transient error correction

Practically, correction is applied using the **GO** button in the **CORRECT** panel, visible only in the angle change parameter view (**Figure 5.27**). The algorithm will identify all angle transients in the data, and interpolate the positions in the frames associated with the quick transients. Note that with real data, differences between frames with correct detections will hardly ever be as low as 0°, while abrupt transitions will rarely be as large as 180°. To find transient changes, the algorithm

searches for abrupt changes over a background of smooth changes. Thus, one must define what constitutes an abrupt change as well as what constitutes a smooth change.
Error correction therefore depends on these parameters, specified in the **CORRECT** panel:
BAD>, value in degrees that defines how large of an angle change (absolute value) constitutes an abrupt transition. Must be larger than 60°.
GOOD<, value in degrees that defines how small the angle change must be to qualify as a smooth change. Cannot be larger than 30°.
MAX LENGTH: The maximum length of transient that will be corrected. The maximum transient length allowed is 5 frames long (the transient length in **Figure 5.26C** is two).

After setting these parameters and pressing the **GO** button, OptiMouse will identify all such transient segments and inform the user of the number of segments and frames detected. If the user approves, these segments will be corrected and assigned to the *interpolated* class (shown in ochre). The modified frames can be easily monitored using the *active setting* view and examining interpolated frames. **Figure 5.27** shows the GUI after fast transition correction. A total of 187 frames were corrected, including the currently shown frame.

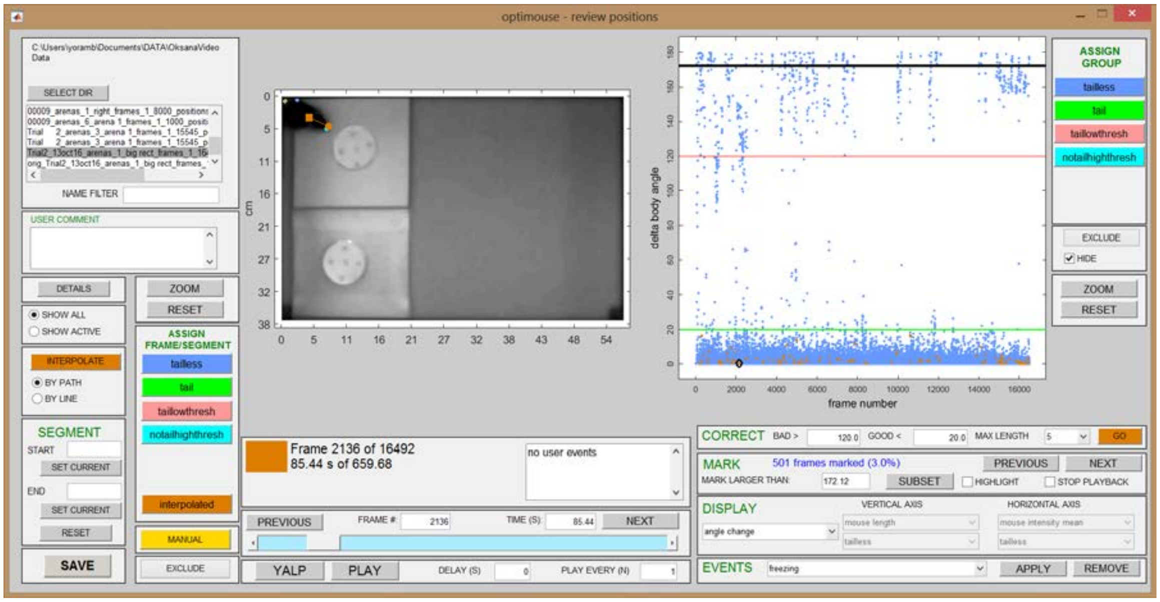

Figure 5.27 Review GUI after application of the transient correction procedure.

5.10. Summary of methods for identifying wrongly detected frames

Below is a summary of the main approaches for identifying frames with a high likelihood of incorrect position detection.

1. Highlight and navigate to frames with abrupt transitions, identified using the *body speed*, *nose speed*, or the *angle change* views (**Section 5.8**). For the angle change parameter, transient changes can be corrected automatically (**Section 5.9**).

2. Highlighting and navigating to frames with NaN values using the *active setting* view. After marking NaN frames with the **SUBSET** button, frames lacking valid positions can be viewed and corrected. Note that even if frames lack a valid position with their current setting, they may still have valid positions with other settings. Recall that, as with all views, the active setting is indicated by the dot color. Active settings for all NaN frames can be changed in a single operation by applying a new setting using polygon based definitions (**Section 5.7.1**).
3. Identifying frames with particular, often outlying, parameter values. Relevant parameters can include object dimensions, arena locations, times during the session, intensity values (of the mouse or the background), or shape parameters. Ideally, frames with particular characteristics will form clusters, facilitating application of a common setting to all such frames in a single operation (using polygon based definition).
4. Identifying frames with discrepancies in parameter values as determined by different settings. While differences in some parameters (i.e. mouse length, area, trim factor, etc.) are expected between different settings, discrepancies in detected positions or angles indicate that at least one setting is wrong. The most useful comparison is probably among body angles. See **Section 5.6.3.1** and **Figure 5.18**. The parameter view and its associated panels facilitate marking and if appropriate, changing settings for all the frames.

5.11. Combining parameter views, navigation tools and keyboard shortcuts to efficiently correct frames

Section 5.5 showed how to identify problematic frames by serially scanning the entire session. The methods described in **Sections 5.6-5.8**, can expedite this procedure considerably. Namely, rather than viewing frames serially, the parameter display views and the **MARK** panel controls allow targeting incorrectly detected frames (see **Section 5.8.3**). The procedure described in **Section 5.5**, starting with step **3**, can then be applied to each frame or segment that needs correction.

Once positions are corrected, irrelevant frames excluded, and event annotations added (if desired), changes should be saved with the **SAVE** button, and the next stage, analysis, can be initiated. The **SAVE** button stores all changes in the associated *positions file* which is used for analysis. Note that the reviewing process may be completed in several working sessions. When a session in opened in the **Review GUI**, previously stored changes are recalled.

6. Analysis

The **ANALYZE** button in the OptiMouse GUI opens the **Analyze GUI** (**Figure 6.1**). Many buttons on the GUI provide a graphical representation of the results. All analysis results can be saved to a data file, for custom analyses of data in individual sessions, or population-level analysis. Some measures can also be displayed on the MATLAB command line as text.

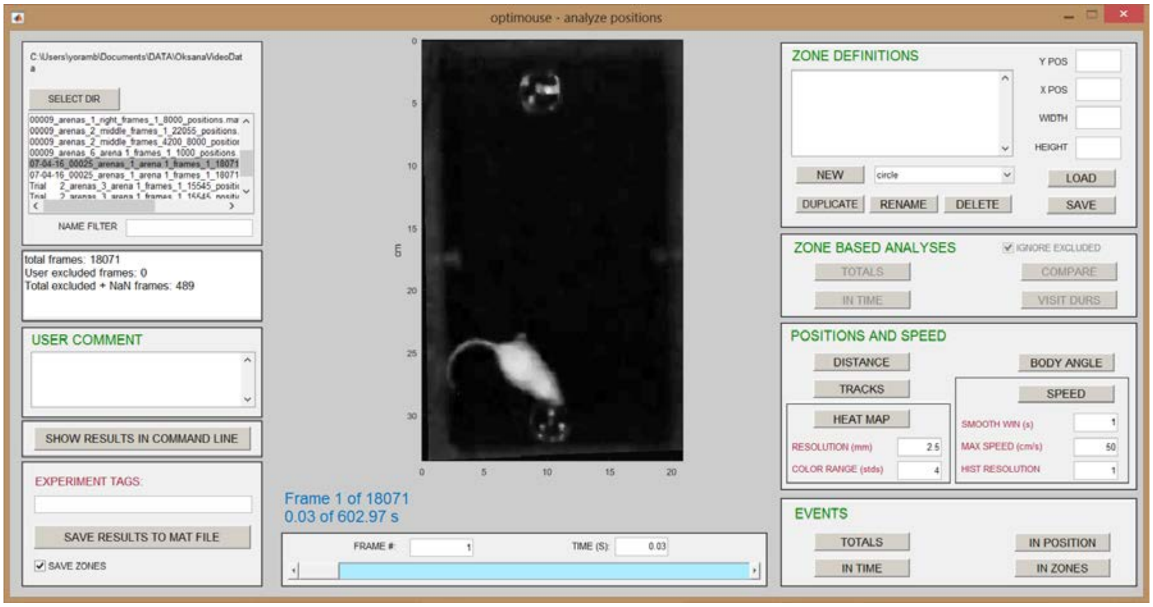

Figure 6.1 The Analyze GUI

6.1. Selecting position files and navigating the video

Analysis requires position files, generated during detection, and potentially modified during the reviewing stage. The upper left box on the **Analysis GUI** lists position files for analysis. The panel below the list shows the total number of frames in the selected file, the number of user excluded frames, and the sum of user excluded and NaN frames (see **Section 5.2.3**).

The **USER COMMENT** panel shows the user comment, if one was entered during the session preparation stage (a comment is now shown in **Figure 6.1**). Navigation tools in the **Analysis GUI** are identical to those in the **Session GUI** (**Section 3.2**). Panels on the right side of the GUI include buttons for various analyses.

6.2. Analysis of positions and speed

6.2.1. Distance

The **DISTANCE** button creates a display of the distance travelled by the mouse *body* as a function of time (**Figure 6.2**).

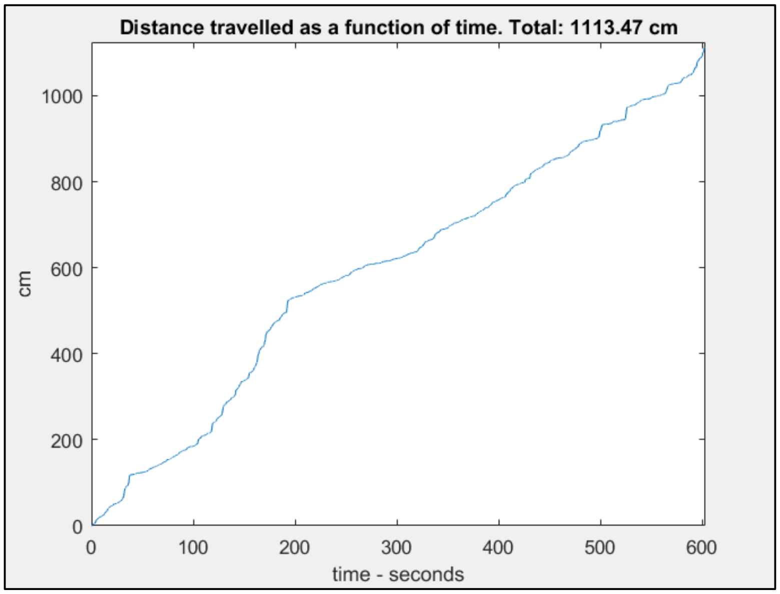

Figure 6.2 Display of distance as a function of time

6.2.2. Tracks

The **TRACKS** button generates two figures showing mouse locations in the arena. One figure shows *body* positions and the other shows *nose* positions (**Figure 6.3**).

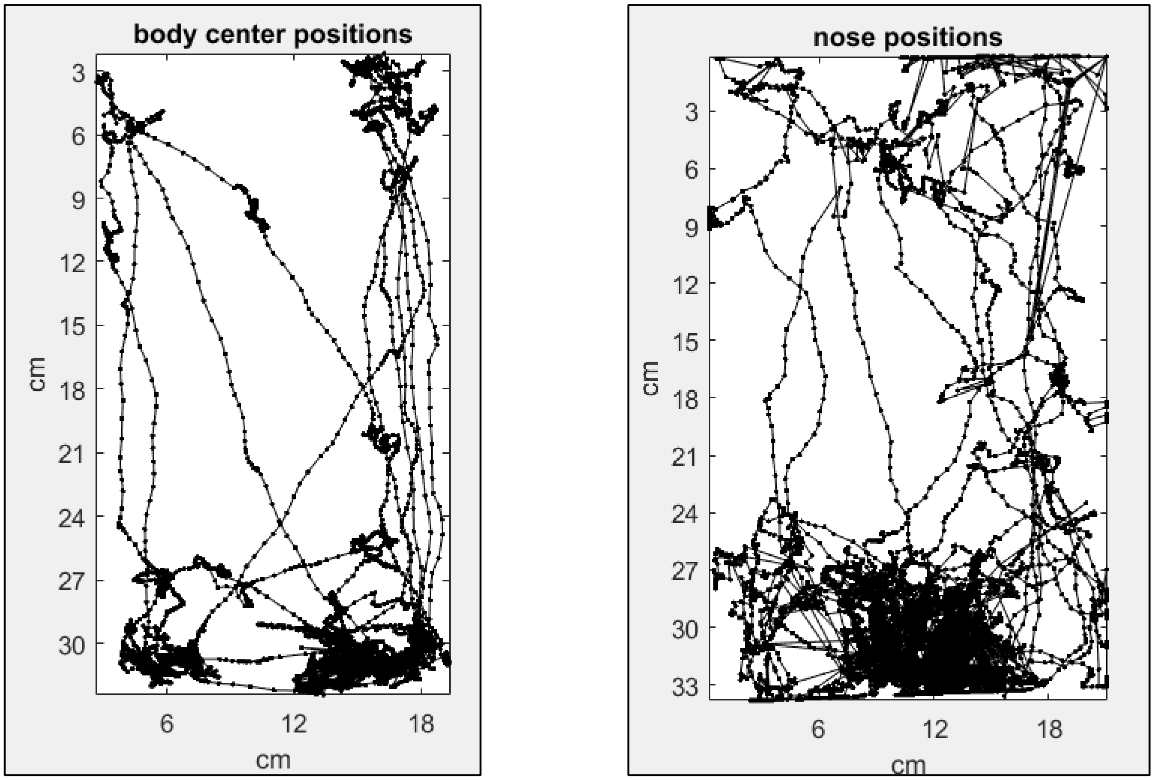

Figure 6.3 Tracks made by the body (left) and the nose (right) across the entire session.

6.2.3. Heatmap

The **HEATMAP** button creates heat maps of body and nose positions in the arena (**Figure 6.4**). Bin colors indicate the frequency of being in each position. The **RESOLUTION** edit box determines the size of the heatmap bins (in mm). The **COLOR RANGE** edit box determines the range of the colormap. The default is four standard deviations, meaning that the color scale will be clipped for values larger than four standard deviations from the mean. This is useful because some bins may contain very high values (for example, if the mouse remains immobile in a certain region for many frames) potentially condensing the dynamic range of the heatmap.

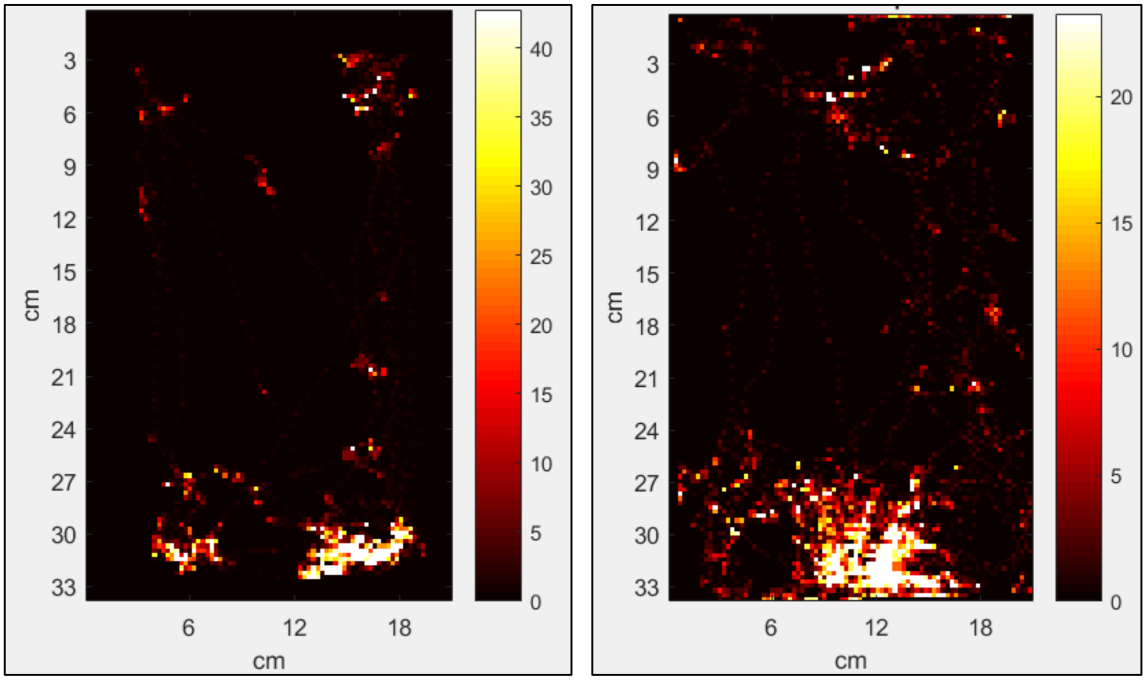

Figure 6.4 Heatmaps of body (left) and nose (right) locations. These figures were generated using bins of 2.5 mm and a 3 SD display cutoff.

6.2.4. Body Angle

The **BODY ANGLE** button shows a polar histogram of the body angles across all frames (**Figure 6.5**). The body angle is defined by the line connecting the body and the nose.

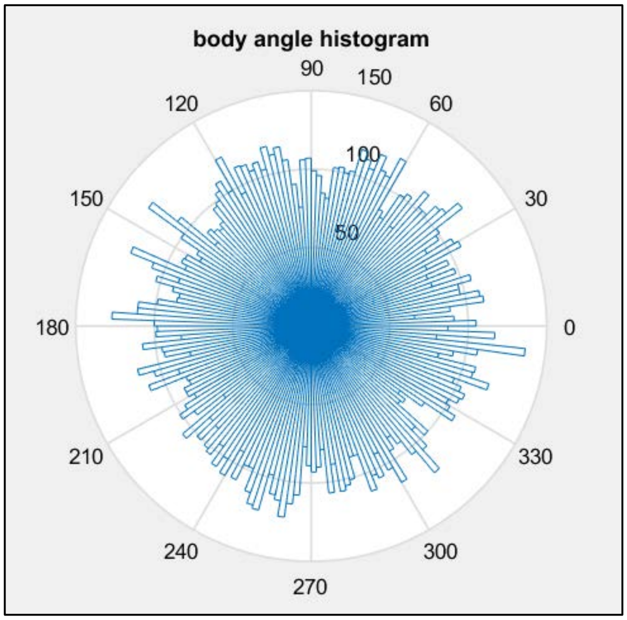

Figure 6.5 Body Angle Histogram

6.2.5. Speed

The **SPEED** button generates a figure with two panels (**Figure 6.6**). On the left is the *body* speed as a function of time. Although the horizontal axes shows all video frames, including excluded or NaN frames, body speed values are not specified for *excluded* and NaN frames. The right panel shows a histogram of speeds. The histogram is rotated so that its vertical axis, representing speed, is congruent with the left panel. Three user-defined parameters control this display. **SMOOTH WIN** determines the temporal smoothing window for the left panel. **MAX SPEED** limits the vertical axis. **HIST RESOLUTION**, determines the bin size (in cm/s) of the speed histogram.

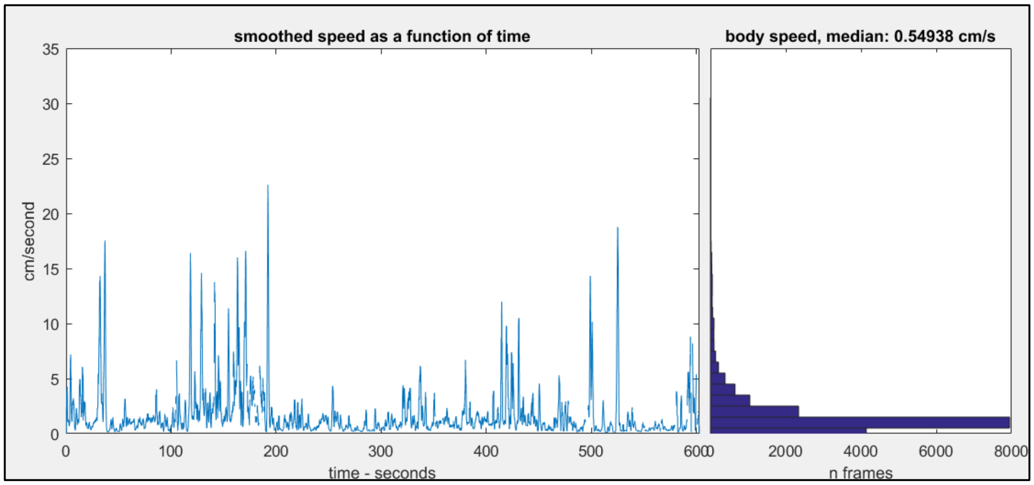

Figure 6.6 Speed Display

6.3. Defining Zones

Many analyses of mouse position data involve the time spent in particular zones within the arena. For such analyses, one or more zones must be defined using the **ZONE DEFINITIONS** panel. Technically, this is identical to arena definition (**Section 3.3.2**), and thus zones can be circles, squares, rectangles, ellipses, polygons, or freehand shapes. The **NEW** button creates a new zone with a shape determined by the listbox to its right. As with arena definitions, zones can be renamed, deleted, duplicated, and modified after creation. **Figure 6.7** shows the **Analysis GUI** after definition of four zones. Note that different zones can overlap.

Zone definitions can be saved (using the **SAVE** button) and loaded (using the **LOAD** button). Zone files are saved in a dedicated *zones* directory, under the main video directory (See **Appendix 1**).

Zones dimensions are saved in mm units, rather than pixels so that zones defined in one video may be applied to another video with a different (pixel/mm) scaling.

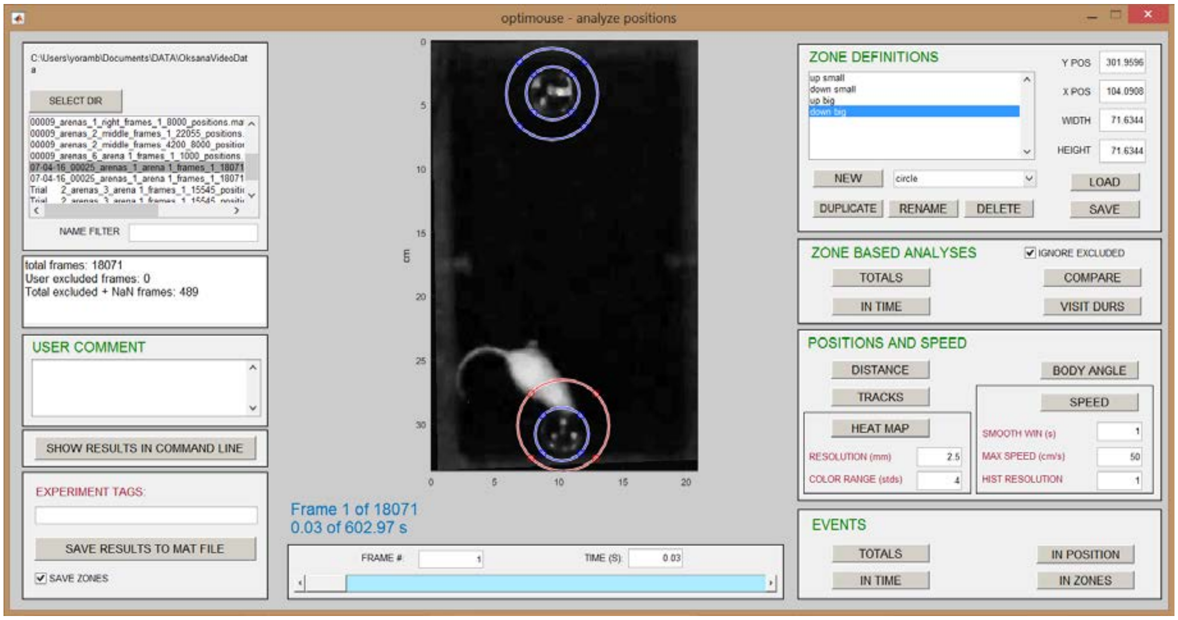

Figure 6.7 **The analysis GUI after definition of four zones. Note that meaningful names were assigned to the zones, and that zones may overlap. After zone definitions, the ZONE BASED ANALYSES panel is active.**

6.4. Zone based analyses

Controls in this panel become active only after one or more zone have been defined (see **Figure 6.7**).

6.4.1. Summarized zone statistics

The **TOTALS** button creates two displays with a summarized description of nose and body positions in each zone (**Figure 6.8** shows the display for nose positions). Bars in the top panel show the *total time* spent in each zone. Bars at the bottom panel show the *enrichment score* for each zone, defined as the ratio between the actual time, and the expected time spent in each

zone. Enrichment scores above 1 indicate preference, whereas values smaller than 1 indicate avoidance of a zone. The expected time is calculated under the assumption of uniform sampling of arena locations. Thus:

$$\textbf{expected time in zone} = \frac{\textbf{total session time} \times \textbf{area of zone}}{\textbf{area of arena}}$$

Since they normalize for zone area, enrichment scores allow comparison of preferences between differently sized zones.

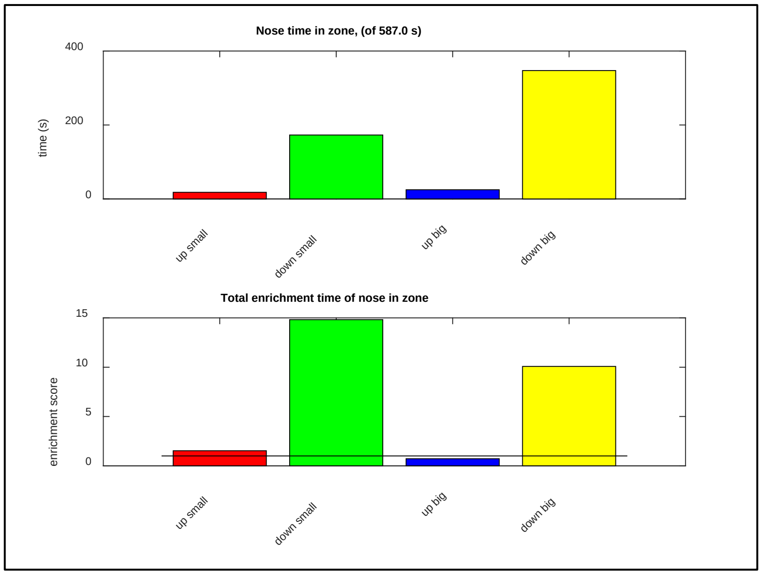

Figure 6.8 **Summarized zone statistics for nose positions in four zones. An analogous display is generated for body positions.**

6.4.2. Ignoring excluded frames

The fraction of time spent in each zone and the enrichment score depend on the total session duration. However, the total duration could either include all video frames, or ignore excluded and NaN frames. Which time base to use is determined by the **IGNORE EXCLUDED** checkbox. The selection influences all analyses in the **ZONE BASED ANALYSES** panel, except those made using the **VISIT DURS** button. The appropriate choice depends on the specific analyses performed.

6.4.3. Zone statistics as a function of time

The **IN TIME** button shows zone statistics as a function of time for nose and for body position. **Figure 6.9** shows the display for nose positions. The top panel shows zone occupancy with time, with different zones indicated on the vertical axes. A colored marker indicates that the nose was inside the zone at the corresponding time point. The middle panel shows the cumulative time spent in each zone. The expected time for each zone is also shown, using lines of the same color.

These lines are barely visible in **Figure 6.9** because the actual number of frames in two of the zones far exceeds the expected values. The expected duration is derived from the zone area and the time elapsed. The bottom panel shows the enrichment scores in each of the zones as a function of time. This is an important measure, because it can often reveal that zone preferences change during the session.

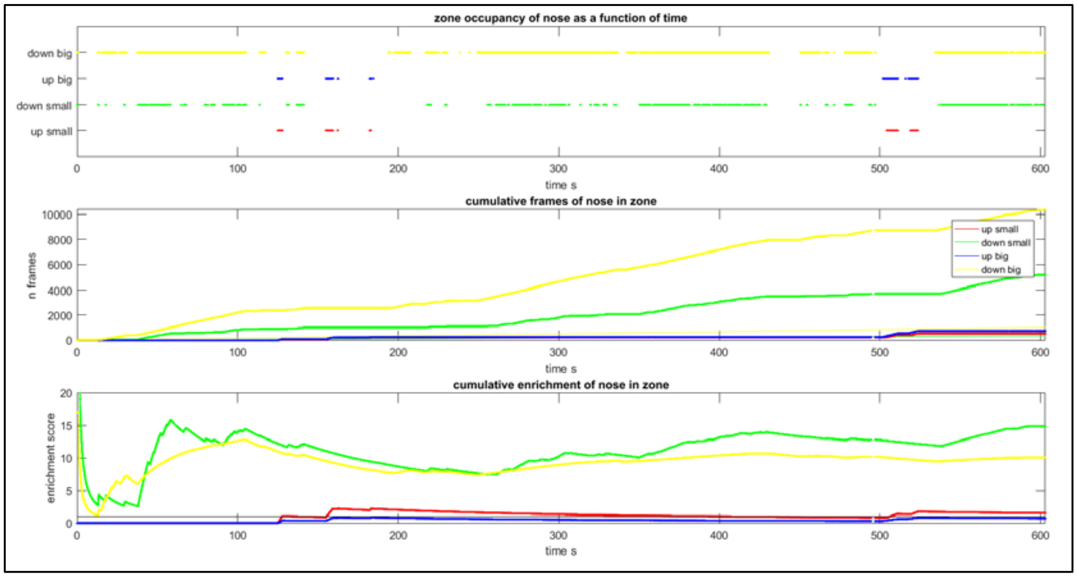

Figure 6.9 **Nose positions in zones as a function of time. An analogous figure is created for body positions.**

6.4.4. Pairwise comparisons among zones

The **COMPARE** button shows pairwise zone preference comparisons (**Figure 6.10**). One figure is created for body positions and one for nose positions. Each figure shows a matrix of pairwise *zone preference indices*. For two zones, A and B, the index is defined as:

$$\text{Zone preference index }(A, B) = \frac{[\text{enrichment zone A} - \text{ enrichment zone B}]}{[\text{enrichment zone A } + \text{ enrichment zone B}]}$$

The index can assume values between -1 and 1. The matrix is antisymmetric around the diagonal.

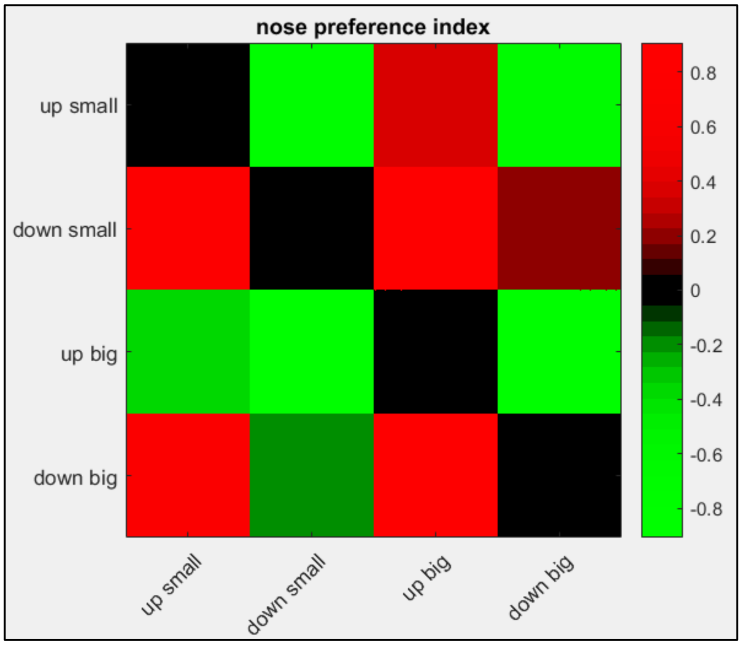

Figure 6.10 Matrix of zone preference indices. The matrix indicates that "down" zones are preferred over "up" zones, and that the smaller zones are associated with higher enrichment scores compared to the larger zones.

6.4.5. Statistics of visit durations

The **VISIT DURS** button shows histograms of visit durations in each of the zones (**Figure 6.11**), defined as the interval between a zone entry and the subsequent exit. Separate figures are created for body and for nose positions with each figure containing one panel per zone. Bar heights indicate sorted individual visit durations.

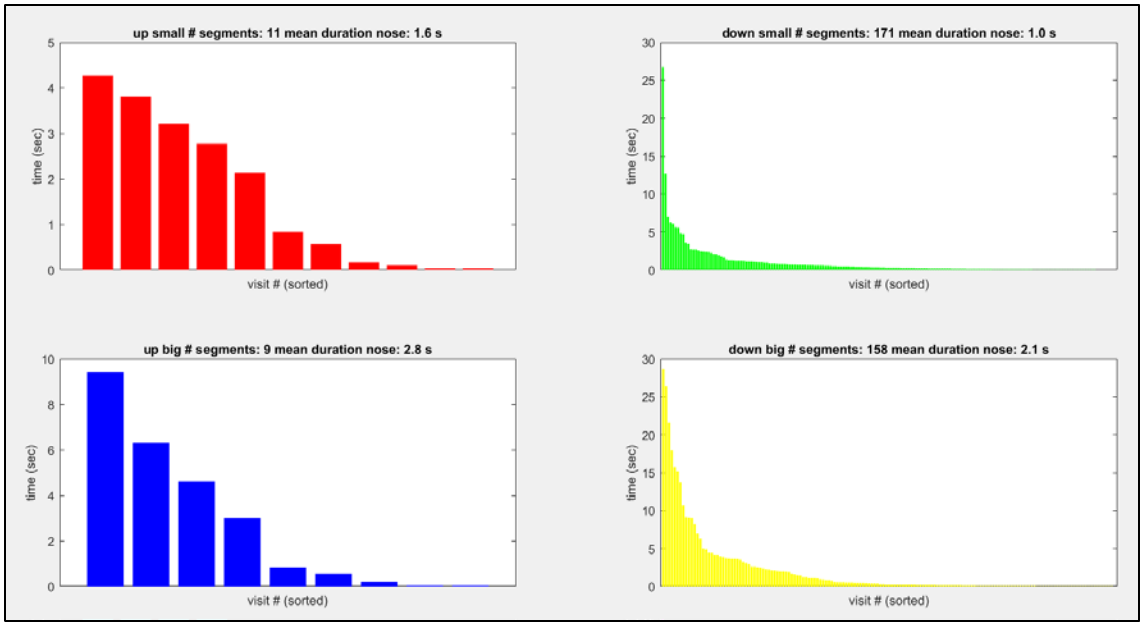

Figure 6.11 Zone visit durations. Shown here are durations based on nose positions.

6.4.6. Application of zones to position displays

When defined, zones are also shown in displays created by the **TRACKS** and **HEAT MAP** buttons (**Figure 6.12**).

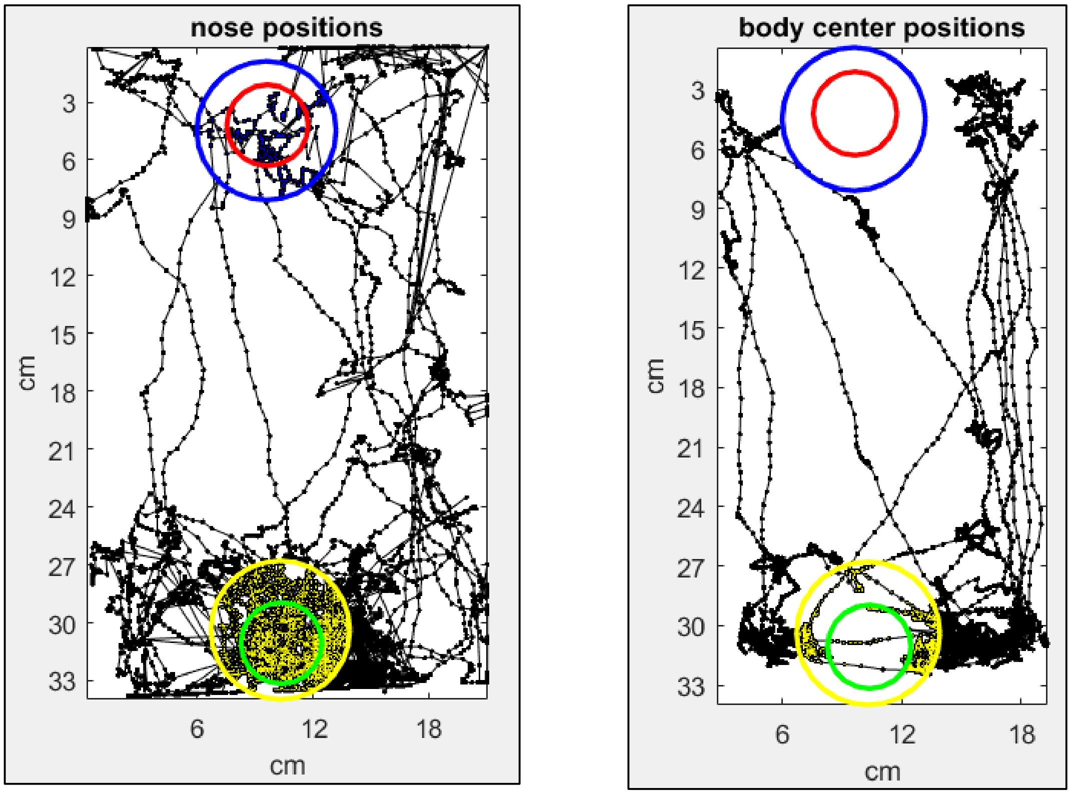

Figure 6.12 Analysis of nose (left), and body (right) tracks after zones have been defined. This display underscores the importance of examining nose, rather than body positions to detect preferences.

6.5. Analyses of event times

Buttons in the **EVENTS** panel will be active if events were defined (see **Appendix 7**) during the reviewing stage.

6.5.1. Total event count

The **TOTALS** button shows the total number of frames associated with each event (**Figure 6.13**). In this and other event related analyses, all frames, including excluded and NaN frames, are considered.

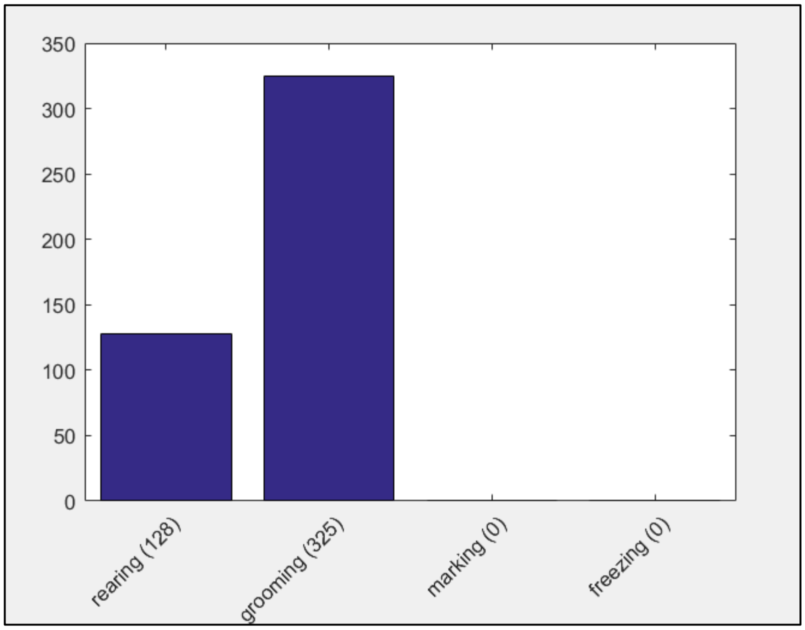

Figure 6.13 Total events display. Four events were defined in the events file (only two were actually assigned to some of the frames).

6.5.2. Event occurrence in time

The **IN TIME** button shows when each of the events occurred (**Figure 6.14**). The lower panel shows mouse speed as a function of time allowing a rough comparison of event occurrence and movement activity. The **SMOOTH WIN, MAX SPEED**, and **HIST RESOLUTION** settings in the **POSITIONS AND SPEED** panel apply to this display as well.

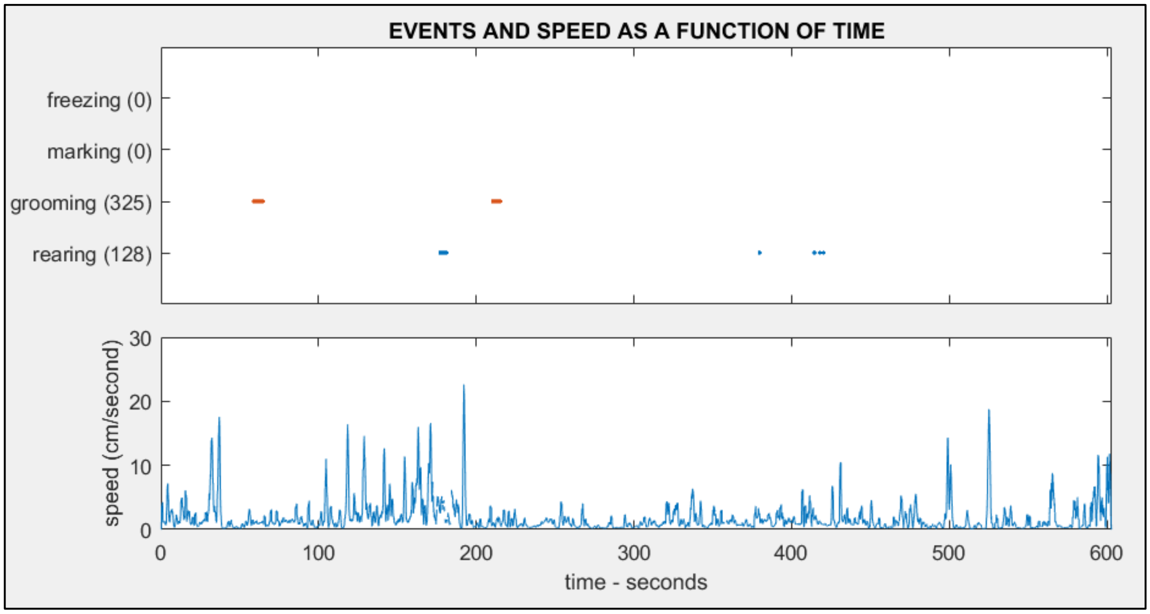

Figure 6.14 Event occurrence as a function of time.

6.5.3. Analysis of event occurrence as a function of position

The **IN POSITION** button generates, for each event, a display showing where it occurred (**Figure 6.15**). In this display, frames associated with the event are shown with red asterisks. In each figure, the left and right panels show nose and body positions, respectively.

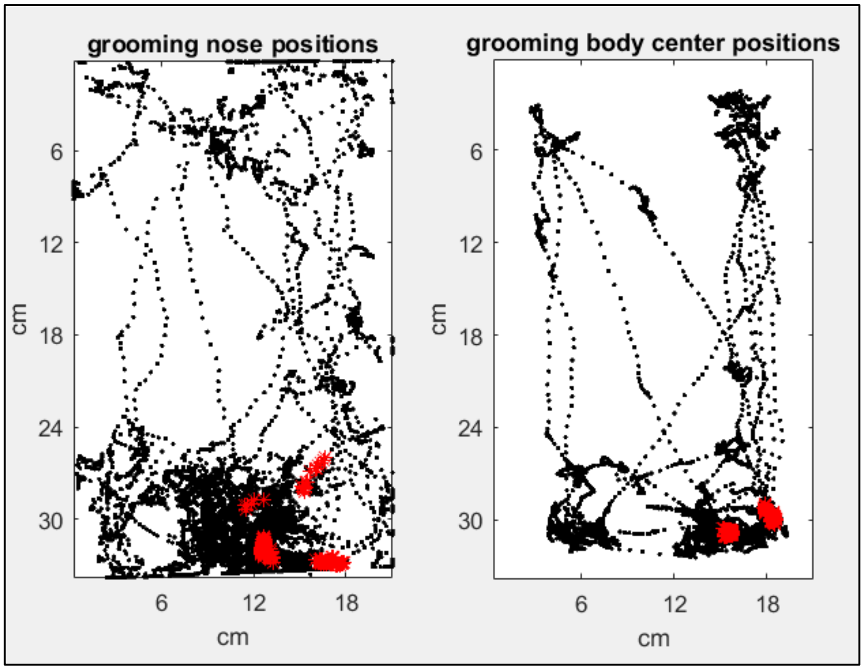

Figure 6.15 Display of one event (grooming) as a function of arena location. Positions associated with the event are indicated by red asterisks. The left panel is for nose positions and the right panel is for body positions.

6.5.4. Analysis of event occurrence in different zones

If zones and events are defined, it is possible to see how many event-frames occurred in each of the zones. The **IN ZONES** button will generate one display for each event that happened at least once in the session (**Figure 6.16**). The left panel of each figure shows the number of frames associated with the event. The right panel shows the *ratio* between the number of in-zone frames associated with the event, and the total number of in-zone frames. For example, if the mouse was inside the zone for 1000 frames, and 200 of these were also associated with the event, the fraction is 0.2. Here, in-zone frames are defined as those in which the *body* (rather than the nose) was in the zone.

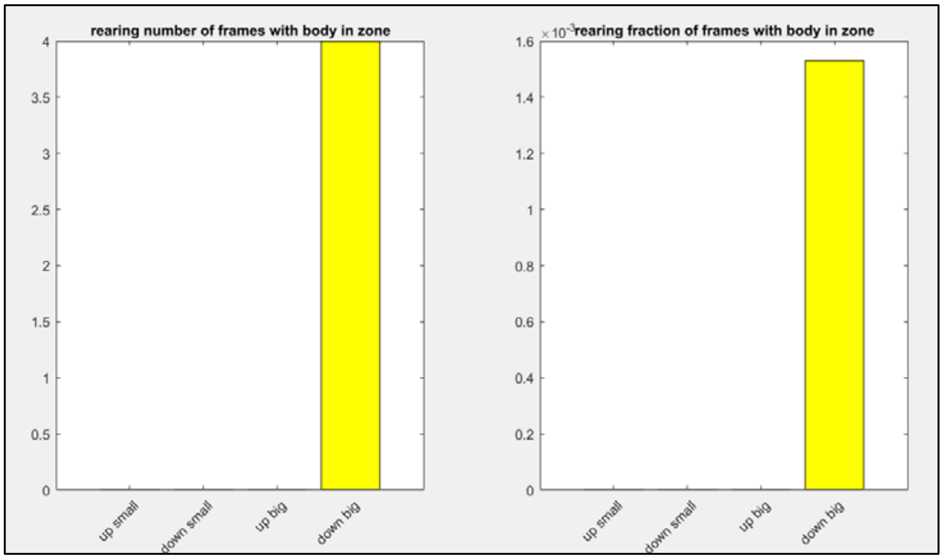

Figure 6.16 **Display of events in zones. In this session, the rearing event occurred only within the zone named "down big".**

6.6. Displaying summary results in the command line

The **SHOW RESULTS IN COMMAND LINE** button provides a summarized report of the results in the command line.

6.7. Saving results to a MATLAB data file

The **SAVE RESULTS TO MAT FILE** saves all analysis results to a MATLAB data file. The file is denoted as a *results file*, and is saved in the *results* directory, under the main OptiMouse video directory. The *results file* contains a single structure, Res, which is described in detail in **Appendix 8**. The structure includes all values associated with the graphical displays and more. It serves as a starting point for analyses not implemented by the GUI.

If the **SAVE ZONE** option is checked, zones will also be saved to a zone file when the **SAVE RESULTS** button is pressed. This is equivalent to pressing the **SAVE** button in the **ZONE DEFINITIONS** panel, except that the zone file name will be set without user input.

6.8. Associating descriptors with files for subsequent analysis

For population level analyses, it may be useful to associate data files with particular tags. When present, tags are saved into the *results file*, allowing subsequent identification of all sessions associated with particular tags (e.g. subjects with a particular phenotype, sessions conducted under certain conditions). Tags are entered as text into the **EXPERIMENT TAGS** edit box. The list will be parsed, with spaces separating distinct tags.

Importantly, to be saved with the *results file*, tags must be first defined in a separate text file, named *optimouse_experiment_tags.txt,* and located in the *optimouse_user_definitions* directory. This file controls for accidental inclusion of tags with typos, which could make it difficult to subsequently identify related sessions. Each line of the text file can include one tag, which is a

character string without spaces. When the **EXPERIMENT TAGS** list is read, OptiMouse checks if the parsed strings are included in the text file. If, and only if they are, they will be saved in the Res structure (**Appendix 8**). Note that tags are *case sensitive.*

7. Appendices

Appendix 1. OptiMouse user directories and files

OptiMouse uses a large number of files and folders. They are described in this appendix by following the analysis of one video file. In this description, folders are shown in **red** font and files are shown in **green** font. To illustrate the various files and folder, we begin with a video folder called **movies**, with only one video file, named **myvid.mp4**. See **Figure A.1** for a graphical display of the folders created during the process.

The session preparation stage

This file is selected for session preparation in the **Prepare GUI** (**Figure 3.1**), the **DEFINE** button is pressed and three arenas are defined in the **Arena Definition GUI** (**Figure 3.2**). These arenas are named, *left*, *center* and *right*.

At the end of the arena definition (after pressing the **SAVE & APPLY** button in the **Arena Definition GUI**) the arenas file, named **myvid_arenas_1 .mat** is created in a folder named **arenas**. The **arenas** folder is located under the main video directory, **movies** in this example.

The arena file contains information about each of the three arenas.

Next, this arena file is applied to the **myvid.mp4** file, a frame range of 1-333 is specified, and session preparation is initiated by pressing the **RUN** button in the **Prepare GUI.**

At the end of the preparation process, three new folders are generated under **movies**, each corresponding to one of the arenas. They are named

myvid_arenas_1_center_frames_1_333
myvid_arenas_1_left_frames_1_333
myvid_arenas_1_right_frames_1_333

Each of these folders contains a number of MATLAB data files. For example, the first folder contains the following files:

myvid_arenas_1_center_frames_1_333_1.mat
myvid_arenas_1_center_frames_1_333_2.mat
myvid_arenas_1_center_frames_1_333_3.mat
myvid_arenas_1_center_frames_1_333_4.mat

Each of these files contains video frames converted to MATLAB format. The video frames are divided into blocks to facilitate loading and processing. The number of blocks thus created is a function of the frame range selected.

In addition, three new files are written to the arenas folder. In this example, they are named

myvid_arenas_1_center_frames_1_333_info.mat
myvid_arenas_1_left_frames_1_333_info.mat
myvid_arenas_1_right_frames_1_333_info.mat

Each of these files contains arena definitions for one of the three arenas defined (center, left and right).

The position detection stage

When the **Detect GUI** is opened, the three arenas are listed in the upper left box. We will continue the explanation with the center arena.
To run detection, settings have to be defined.
If the **SAVE** button is pressed, then a *settings file* is created. Unless the user specifies a different name, this file, which was created will be named
detection_settings_myvid_arenas_1_center_frames_1_333.mat and saved under a new directory called **detection_settings**, located under the main video directory.

When the **RUN** button pressed, detection is initiated. Regardless of whether the user explicitly saved the settings (as described above), they are written into a file in the **detection_settings** folder. The file format is identical to that saved by the user, but it has a unique name identifier (associated with the time of its creation). In our example, the settings file is called:
detection_settings_myvid_arenas_1_center_frames_1_33307-Nov-2016 09_46_53.mat
The unique identifier is required for the batch processing option (**Appendix 4**).

The results of detection are saved in another file, located with the **positions** directory, which is itself under the main video directory. The position file in our example is called **myvid_arenas_1_center_frames_1_333_positions**.

The reviewing stage

The listbox on the upper left side of the **Review GUI** includes all positions file in the **positions** directory. The changes made to the file are saved in the *position file*. No new files are created at this stage.

The analysis stage

If saved, zone definitions made in this stage are stored in a zone file under the **zones** folder, under the main video directory. The zone file in our example, given its default name, is **myvid_arenas_1_center_frames_1_333_zones_1.mat**.
The **SAVE RESULTS TO MAT FILE** button saved the results in another matlab data file, under the **results** directory.
In our example, the default name for the file is
myvid_arenas_1_center_frames_1_333_result_1.mat.

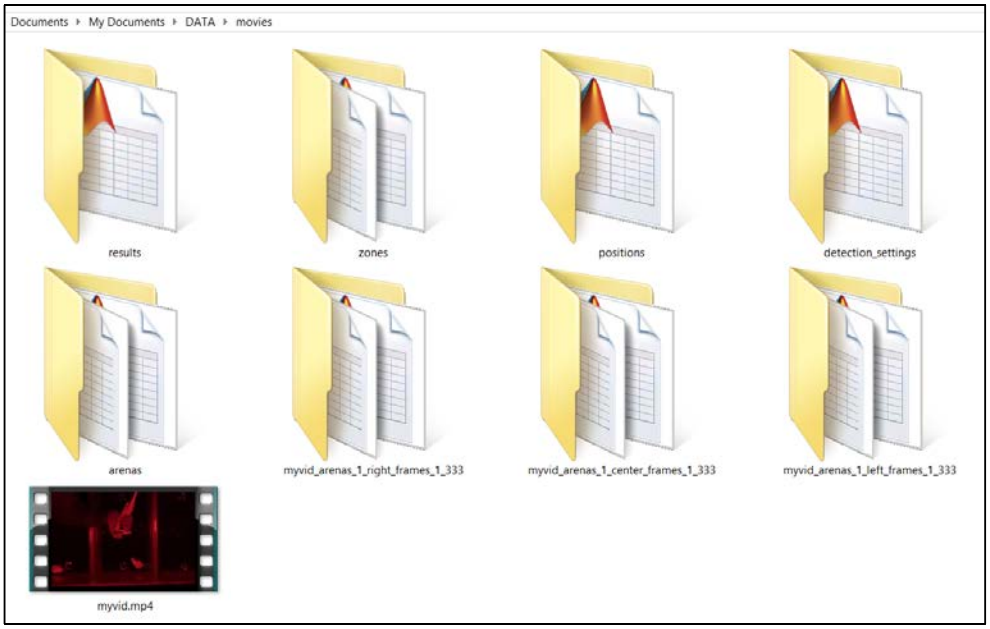

Figure A.1 **Graphical representation of files created under the main video folder (here named movies). All directories and files shown here resulted from the analysis of three arenas for one video.**

Appendix 2. Inside the arena preparation stage

Arena preparation involves transforming each video frame to a MATLAB format, and extracting the relevant part of the frame corresponding to each of the arenas. The conversion to MATLAB data files results in considerably faster processing than would be possible by directly reading each video frame. The extracted segment, per arena, is the smallest rectangle containing the entire arena. The arena margins (the regions between the arena boundaries and the enclosing rectangle) are assigned with the median intensity value across all frames within the frame block. The data is saved as unsigned integer (uint8 data type) grayscale matrices.

As described in **Appendix 1**, the process yields a new folder for each arena.

Within each of these folder are relevant video frames, divided into blocks, each containing **100** frames. This value represents a tradeoff between minimizing the number of blocks and the cost of loading them *vs*. the memory load associated with each block. The block size is determined by the variable *framesperblock* defined in the file *prepare_arena_data.m*.

Once the arena preparation is complete, an "info" file, containing all the definitions for each specific arena is created in the *arenas* folder.

Appendix 3. The prepare batch file

When the **BATCH** button in the **Prepare GUI** is pressed, the command that would be otherwise executed by the **RUN** button is written into a MATLAB text file. The file is named *prepare_arena_batch.m* and is located in the *optimouse_user_definitions* folder.

Each press of the **BATCH** button adds six lines to the file. Using the example of **Appendix 1**, a frame range of 1-600, and starting with an empty batch file, after pressing the **BATCH** button, the batch will contain the following lines (font colors are those assigned by the MATLAB editor):

```
% batch command from 07-Nov-2016 10:34:58
% video file: myvid
% arena file: myvid_arenas_1
% frames: 1 to 600
disp('now running
prepare_arenas(C:\movies\myvid.mp4,C:\movies\arenas\myvid_arenas_1.mat,1,600,
100,)')
prepare_arenas('C:\movies\myvid.mp4','C:\movies\arenas\myvid_arenas_1.mat',1,
600,100,'')
```

The first four lines are meant as documentation providing information about the date, original video file name, arena file and frame range. In the MATLAB editor, lines beginning with the % character are considered as comments and ignored.
The fifth line (beginning with the word *disp*) will result in a command line report allowing the user to monitor progress when the batch file is running. The sixth line, beginning with the word *prepare*, is the actual command.

As described in **Section 3.6**, the user is responsible for checking that the previously executed lines in the batch file are deleted or converted to comments. Otherwise they will be executed again. Commenting lines, rather than deleting them, can make the batch file serve as a work log of sessions that were prepared. Pressing *ctrl r* in the MATLAB editor turns selected lines to comment lines by adding the % character at their start.

Appendix 4. The detect batch file

When the **BATCH** button in the **Detect GUI** is pressed, the command that would be otherwise executed by the **RUN** button is written into the file **calculate_positions_batch.m** which is located in the **optimouse_user_definitions** folder. This file is similar to the Prepare GUI batch file described in **Appendix 3**.
Each press of the **BATCH** button adds five lines to the file. Using the example of **Appendix 1**, a frame range of 1 to 333, and an empty batch file, after pressing the **BATCH** button, the file will contain the following lines:

```
% batch command from 07-Nov-2016 11:00:38
% arena file: myvid_arenas_1_left_frames_1_333_info
% position file: myvid_arenas_1_left_frames_1_333_positions
disp('now running
calculate_positions_mm(C:\movies\detection_settings\detection_settings_myvid_
arenas_1_left_frames_1_33307-Nov-2016 11_00_38.mat)')
calculate_positions_mm('C:\
movies\detection_settings\detection_settings_myvid_arenas_1_left_frames_1_333
07-Nov-2016 11_00_38.mat')
```

The first three lines are comments providing information about the date, the arena file, and the output (position) file name. The fourth line (beginning with the word *disp*) will result in a command line display allowing the user to monitor progress when the batch file is running. The

fifth line, beginning with the word *calculate*, is the actual command. Note that the input to the calculation function (*calculate_positions_mm*) is MATLAB file name. This is the *detection settings* file described above (**Appendix 1**), associated with a unique name identifier. This file contains all the required parameters for detection, including multiple settings and background images.

Appendix 5. Defining custom functions

As described in **Section 4.11**, OptiMouse allows the incorporation of user defined detection algorithms. This is an advanced feature that requires familiarity with the MATLAB programming language. Custom algorithms are specified as dedicated MATLAB m files. The files should define functions that accept pixels intensity values (in one frame), return nose and body positions and ideally, as described below, other parameters as well.

All detection functions, whether built-in (all built-in algorithms are implemented via the file *get_mouse_position_mm.m* file**)** or user defined, are called in two contexts. The first is when the algorithm is selected in the **Detect GUI** and a frame or a parameter is modified (i.e. when an update is required)**.** In this situation, the detection algorithms are called by the function *update_arena_images.m*. The second context is during the detection procedure, whether using the **RUN** button or the **BATCH** option. In this context, the calling function is *calculate_positions_mm.m*, which was already mentioned in the context of the *calculate_positions_batch.m* file.

All custom functions must be "declared" in the file *user_defined_detection_function_description.m* located in the *optimouse_user_definitions* folder. The file returns a structure array, named *user_detection_functions.* Each element in the array corresponds to one function. The structure has the following fields:

runstring: the string that will be evaluated to run the algorithm.

name: the name of this algorithm in the **Detect GUI** listbox. This variable is also a string.

param_names: a cell array of parameter names. These names will appear in the **Detect GUI** when the function is selected from the list of algorithms. Up to 5 parameters can be defined and accessed from the **Detect GUI**. All params are numerical, and must be single numbers (not arrays).

param_range: a cell array, with the same number of elements as the param_names, specifying the minimal and maximal values for the corresponding parameters.

Below is the beginning of the *user_defined_detection_function_description* file, which contains a definition of one such function:

```
user_detection_functions = user_defined_detection_function_description

% a funciton with definition of user defined functions for detection
user_detection_functions = [];
% %
% %
% Result =
user_definedfunction(MedianRemovedImage,trim_cycles,GreyThresh_fact,user_pname1,user_pname2);
```

```
user_detection_functions(1).runstring = 'Result =
user_defined_detect_func_1(ThisFrame,trim_cycles,GreyThresh_fact,shift1,shift2);';
user_detection_functions(1).name = 'user_func 1';
user_detection_functions(1).param_names{1} = 'shift1';
user_detection_functions(1).param_range{1} = [-10 10];
user_detection_functions(1).param_names{2} = 'shift2';
user_detection_functions(1).param_range{2} = [-10 10];
```

When the **Detect GUI** is opened, it searches for the *user_defined_detection_function_description.m* file. If the file exists, and if it contains algorithm definitions, then these algorithms will be added to the **ALGORITHM** listbox. When one of the user defined functions is called from the **ALGORITHM** listbox, all parameters associated with this file (up to 5) are added to the **USER PARAMETERS** panel. **Figure A.2** shows the **Detect GUI** after the function declared above (*user_func 1*) was selected. When values are entered into the shift1 and shift2 edit boxes in the **USER PARAMETERS** panel, OptiMouse will check if entered values are within the range defined in the *user_defined_detection_function_description.m* file (-10 to 10 in this example).

Whenever a user defined algorithm is selected from the list, it is applied to the current frame (by executing the *runstring* file entry defined for it) and the resulting position detection is plotted over the image.

For example, in the present example, OptiMouse will attempt to execute the following command:
'Result = user_defined_detect_func_1(ThisFrame,trim_cycles,GreyThresh_fact,shift1,shift2);'
For this to result in valid detection, several conditions must be met.

1. There must be a function in the MATLAB path named *user_defined_detect_func_1.m*. This function should be located in the *optimouse_user_definitions* directory.
2. The function must accept inputs as specified in the commend string. In this call, the first three parameters are variables that are defined within the functions that call the algorithms. Specifically:
   ThisFrame is the frame image, after background subtraction, as determined in the **Detect GUI**.
   trim_cycles is the number of cycles of trimming, as determined in the **Detect GUI.**
   GreyThresh is the user specified **THRESHOLD** as determined in the **Detect GUI.**
   Any user defined function must take as input a frame image, otherwise it cannot detect positions, but the other parameters are not necessarily required. For example, one could write an algorithm that does not need *trim_cycles* or the *GreyThresh* parameters.
   The other parameters are optional. In this example, there are two additional parameters, *shift1* and *shift2* which are retrieved from the GUI and passed to the function. These parameters are also optional, and are limited to 5. They must be numerical variables (not strings, or arrays). The parameter names in the function call (as specified in the *runstring* field) must be identical to those specified in the *param_names* field of the function.
3. As for the output side, the function returns a single structure called *Result*, with the following fields:

Absolutely required values:

Result.mouseCOM	=	1x2 array of mouse body center positions specified in pixels. The first element is the X dimension, the second is the Y dimension.
Result.nosePOS	=	1x2 array of mouse nose positions in pixels

Optional values for plotting results in the Detect GUI:

Result.BB	=	1 x 4 element array specifying the bounding box of the mouse. (X,Y,Width,Height)
Result.PerimInds	=	indices of mouse perimeter pixels. Specified as a cell with two arrays. The first for the column, and the second for the row indices of all perimeter pixels.
Result.ErrorMsg	=	a string with an error message if detection did not succeed
Result.tailbasePOS	=	1x2 array of tail base positions in pixels
Result.tailendPOS	=	1x2 array of tail end positions in pixels

Optional values for the parameter display in the Review GUI:

Result.GreyThresh	=	MATLAB determined grey threshold
Result.TrimFact	=	ratio of length of mouse before and after peeling.
Result.MouseArea	=	mouse area in pixels
Result.MousePerim	=	mouse perimeter in pixels
Result.ThinMousePerim	=	thin mouse perimeter in pixels
Result.BackGroundMean	=	intensity value of image background
Result.MouseMean	=	mean intensity value of mouse object
Result.MouseRange	=	range of intensity values of mouse object
Result.MouseVar	=	variance of intensity values of mouse object

We reiterate that the only absolutely required values are mouse COM and nosePOS. The other values are required for plotting the output of the result in the **Detect GUI**, or for assisting with frame detection in the parameter display in the **Review GUI**. Note that if these parameters are not returned as outputs, OptiMouse will replace them with 0 or NaN values. The consequence is that some display option in the **Detect GUI** or **REVIEW GUI** will not be available. For example, the yellow bounding box and mouse perimeter could not be viewed if the corresponding parameters are not returned. Likewise, if the MouseArea parameter is not returned, then it would not be possible to view it in the parameter pair view display.
More information about these fields can be obtained by viewing the documentation in the file *get_mouse_position_mm.m* that contains all the built-in detection algorithms.

The file *user_defined_detection_function_description* in the *optimouse_user_definitions* directory is contains definitions of four custom functions. The MATLAB *.m files for these

functions are also included. Note that these function serve only to illustrate how the user defined function concept works, and are not useful for detection. The functions are:
user_defined_detect_func_1.m
user_defined_detect_func_2.m
user_defined_detect_func_3.m
user_defined_detect_func_4.m
The first three functions are based on algorithms in *get_mouse_position_mm.m*. They differ in the *head method* parameter, which defines the detection algorithm, and also in the names and numbers of custom parameters defined. In these examples, for illustration, the parameters apply a shift on the positions detected by the *get_mouse_position_mm* function. The fourth function returns random X and Y positions for the nose and the body. The image input is only used to derive frame dimensions. This function does not return any other parameter other than the X and Y position (**Figure A.4**).

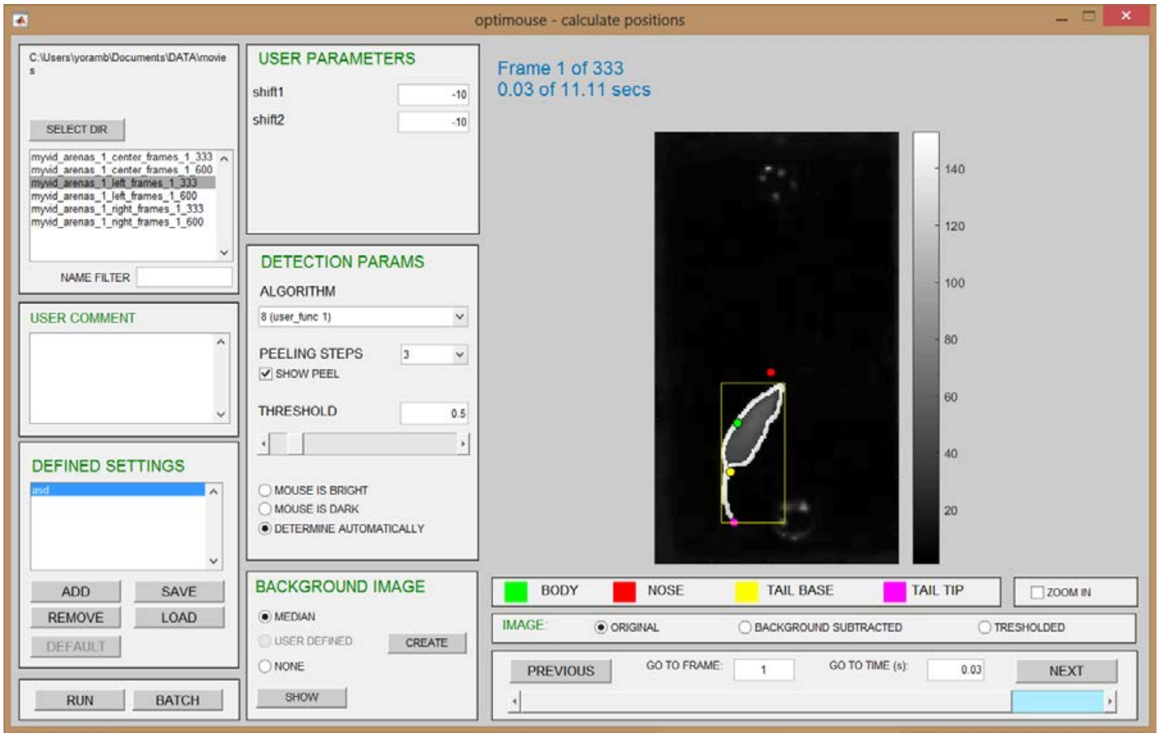

Figure A.2 Detect GUI when a user defined function with two parameters are selected.

Figure A.3 and **A.4** shows the **Detect GUI** after selecting of two different user defined functions. One with five parameters, and one without parameters.

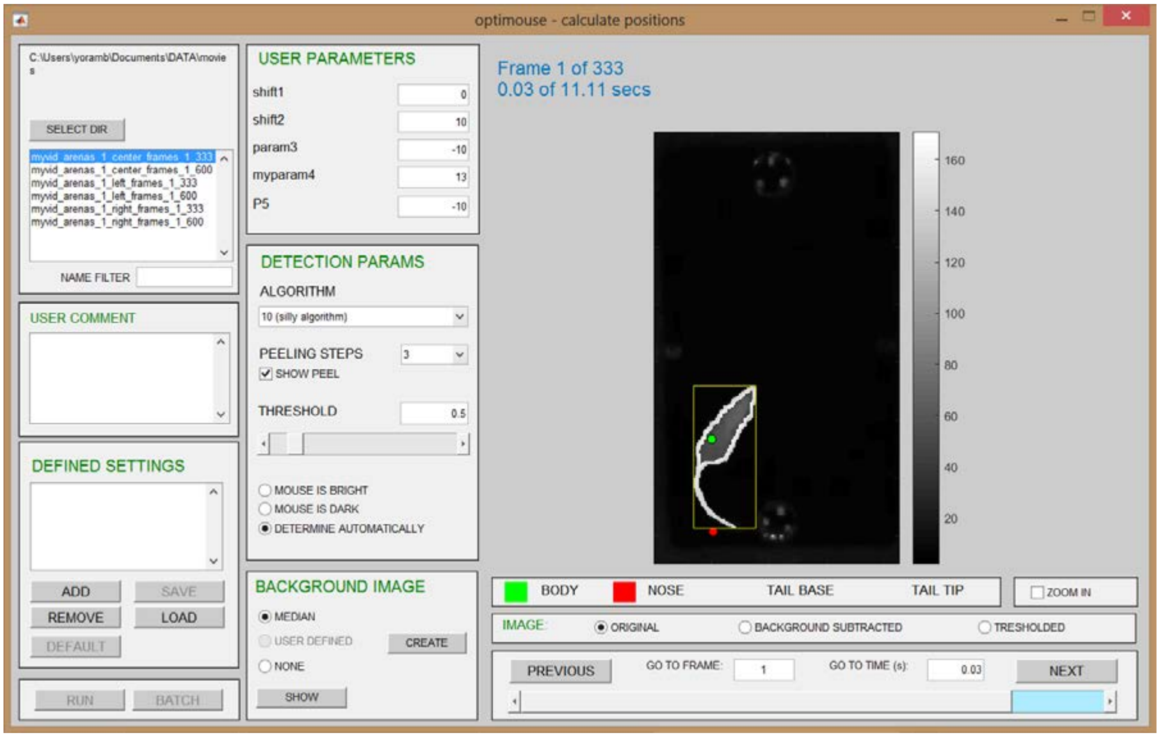

Figure A.3 Detect GUI when a user defined function with five parameters are selected. This function does not return the tail base or tail tip

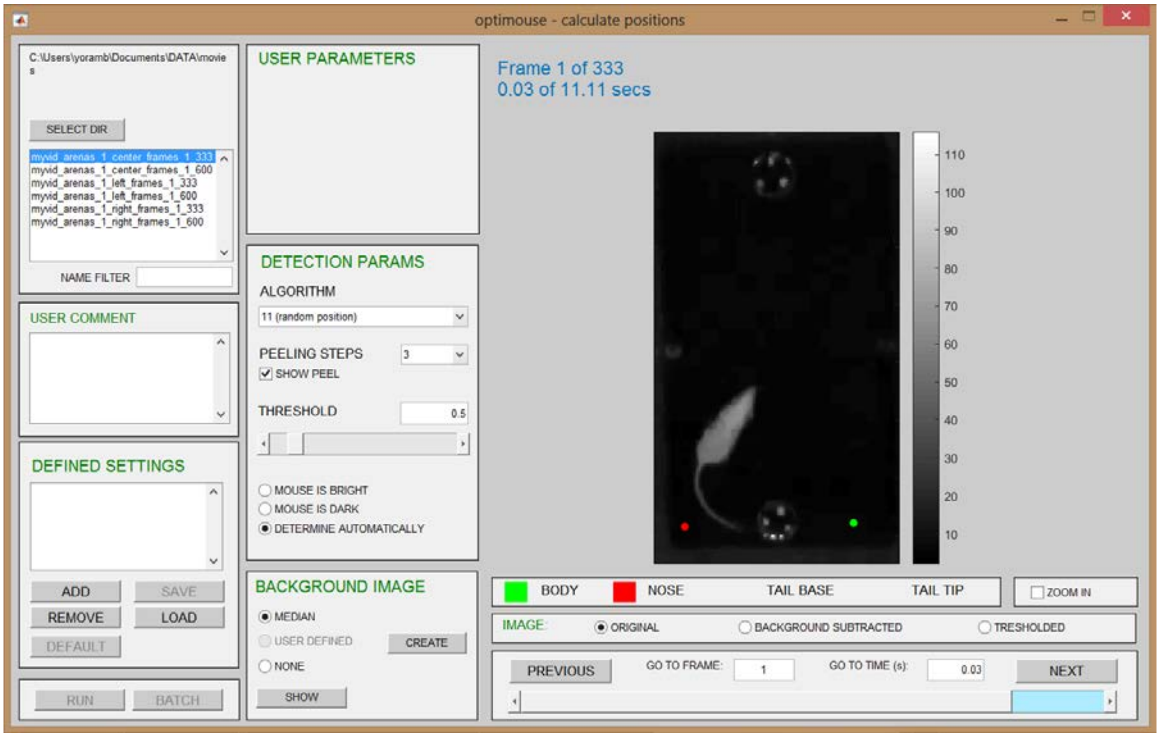

Figure A.4 A user defined function that does not return any outputs other than random tail and nose positions.

Appendix 6. Keyboard shortcuts

Keyboard shortcuts for the Detect GUI

Navigation

> move forward one frame

< move back one frame

shift > move forward one second

shift < move backward one second

Keyboard shortcuts for the Review GUI

Navigation

> move forward one frame

< move back one frame

shift > move forward one second

shift < move backward one second

alt > forward playback (continuous)

alt < reverse playback (continuous)

control > move to the next marked frame

control < move to the previous marked frame

Frame assignment

1,2,3,4,5,6 assign settings (1,2,3,4,5,6, respectively) to current frame or segment (if defined).

x exclude current frame or segment

i interpolate frame positions from first to last frames in segment

Segment definition

s set current frame as segment start

e set current frame as segment end

Annotation

a apply selected event (from the listbox in the **EVENT** panel) to current frame or segment (if defined).

r remove selected event from current frame or segment.

Appendix 7. The event file

To annotate frames in the **Review GUI** (using the **EVENT** panel listbox), the list of valid events must first be defined in the file *user_annotation_events.m* in the *optimouse_user_definitions* folder. The events are defined as a cell array of strings. To illustrate, in the example of the event file shown below, the **EVENT** listbox will contain 4 events, called rearing, grooming, marking, and freezing.

```
function annotation_events = user_annotation_events
% user defined events
annotation_events{1} = 'rearing';
annotation_events{2} = 'grooming';
annotation_events{3} = 'marking';
annotation_events{4} = 'freezing';
```

Event can be added by adding lines:

```
function annotation_events = user_annotation_events
% add or remove events using the same conventions used in this file
annotation_events{1} = 'rearing';
annotation_events{2} = 'grooming';
annotation_events{3} = 'marking';
annotation_events{4} = 'freezing';
annotation_events{5} = 'walking';
annotation_events{6} = 'running';
annotation_events{7} = 'jumping';
annotation_events{8} = 'galloping';
```

or removed:

```
function annotation_events = user_annotation_events
% add or remove events using the same conventions used in this file
annotation_events{1} = 'rearing';
annotation_events{2} = 'grooming';
```

```
function annotation_events = user_annotation_events
% add or remove events using the same conventions used in this file
annotation_events = [];
```

Appendix 8. The results file

The *results file* includes a single structure, called Res with various fields containing analyzed position data. In addition, some of the fields in Res are structures themselves, each which multiple fields, some of which are also structure arrays. Thus, Res is an elaborate multi-level structure that contains not only analysis results, but also all the raw position data required for analysis, as well as inherited information from previous stages. To facilitate the description below, the fields are organized into groups. Information about most of these fields can be found in the text of the manual.

General attributes

Res.position_file full name of position file analyzed.
Res.experiment_tags cell array with user entered experiment_tags associated with this file.
Res.deltaT time interval in seconds between frames
Res.frame_times array of times for each frame, including excluded frames
Res.good_frame_times array of times for the non-excluded frames

Res.good_frames a logical array, with a value of 1 for every frame that is not excluded and does not have a NaN entry for position. 0 otherwise.

General movement statistics

Res.cms_travelled array of doubles with the cumulative distance travelled in each frame.
Res.total_cms_travelled total distance travelled in cm, equivalent to the last element in cms_travelled
Res.delta_body_cm_s body speed in each frame (cm/s)
Res.smoothed_speed smoothed body speed in each frame. Smoothing parameters as specified in the GUI.
Res.smooth_win_sec smoothing window for speed as specified in GUI.
Res.smooth_points number of points used for smoothing
Res.mean_body_speed mean body speed in cm/s
Res.median_body_speed median body speed in cm/s
Res.std_body_speed standard deviation of body speed

Zone related measures

Res.zone_names a cell array with the names of user defined zone names
Res.ZV cell array with each element containing the vertices of each of the zones. The number of vertices depends on the type of zone.
Res.ZAcm2 array of doubles containing area of each zone (in cm2)
Res.allZPbody logical ZxN array, with Z being the number of zones, and N being the number of good (i.e. non excluded) frames. A value of 1 in element (z,n) indicates that the body position was inside zone z in frame n
Res.allZPnose same as allZPbody with values indicating nose rather than body positions.
Res.cumsumZPbody double ZxN array, with the cumulative number of frames spent in each zone as defined by body position.
Res.cumsumZPnose double ZxN array, with the cumulative number of frames spent in each zone as defined by nose position.
Res.cumZbody_enrichment double ZxN array, with cumulative enrichment score, of body position in each frame for each zone.
This value depends on the total number of good frames, which in turn depends on whether NaN frames were excluded or not.
Res.cumZnose_enrichment double ZxN array, with cumulative enrichment score, of nose position in each frame for each zone.
This value depends on the total number of good frames, which in turn depends on whether NaN frames were excluded or not.
Res.totalZbody_enrichment ZX1 array with the total enrichment of the body in each zone. This value is the same as the last element of cumZbody_enrichment.
Res.totalZnose_enrichment ZX1 array with the total enrichment of the nose in each zone. This value is the same as the last element of cumZnose_enrichment.
Res.totalFramesZPnose ZX1 array with the total number of frames that the nose spent in each zone

Res.totalFramesZPbody ZX1 array with the total number of frames that the body spent in each zone
Res.totalTimeZPnose ZX1 array with the total time that the nose spent in each zone (in seconds)
Res.totalTimeZPbody ZX1 array with the total time that the body spent in each zone (in seconds)
Res.totalTimeZPbody_CM2 ZX1 array with the total time that the body spent in each zone, normalized by area of zone (i.e. total time per cm2)
Res.totalTimeZPnose_CM2 ZX1 array with the total time that the nose spent in each zone, normalized by area of zone (i.e. total time per cm2)
Res.fractionTimeZPbody ZX1 array with the fraction of time that the body spent in each zone. This value depends on the total number of good frames, which in turn depends on whether NaN frames were excluded or not.
Res.fractionTimeZPnose ZX1 array with the fraction of time that the nose spent in each zone. This value depends on the total number of good frames, which in turn depends on whether NaN frames were excluded or not.
Res.pairwise_zone_pref_index_body A ZxZ matrix of pairwise preference indices as measured by body positions for each pair of zones. The index assumes values between -1 and 1. The matrix is antisymmetric around the diagonal
Specifically:

$$\text{pairwise\_zone\_pref\_index\_body}(i,j) = \frac{[\text{totalZbody\_enrichment}(i) - \text{totalZbody\_enrichment}(j)]}{[\text{totalZbody\_enrichment}(i) + \text{totalZbody\_enrichment}(j)]}$$

Res.pairwise_zone_pref_index_nose A ZxZ matrix of pairwise preference indices as measured by nose positions for each pair of zones. Analogous to pairwise_zone_pref_index_body, but refers to nose positions
Res.nose_zone_visits cell array, with one element for each zone, where each element is an N by 2 array with N being the number of segments within a zone. Thus, each row corresponds to one segment. The first column is the entry time. The second column is the exit time.
Res.body_zone_visits same as nose_zone_visits, but for body rather than nose positions.
Res.nose_zone_durations cell array, with one element for each zone, where each element is an N by 1 array of segment durations.
Res.body_zone_durations same as nose_zone_durations, but for body rather than nose posititions.

Event related measures

Res.event_names a cell array with user annotated event names. Only events which are non-empty are included (that is, events that are not associated with any frame in the session are not included.
Res.event_inds cell array, with one element for each event (corresponding to the event_names field), with each element itself an array of indices of the frames associated with each of the events. The number of elements for each such event is thus the number of frames associated with the event
Res.N_this_event_in_this_zone An M x Z array, where M is the number of events, and Z is the number of zones. Each element contains the number of event frames occurring within each of the zones.

Res.fraction_events_in_this_zone like N_this_event_in_this_zone, but normalized for the total number of (non excluded) frames.

Position data

Position data is specified in **pD**, itself a structure which is inherited from the position file containing all the position data (pD stands for position Data).

Final position data

Res.pD.frame_class A 1XN array with the active setting for each frame. N is the total number of session frames *including excluded and NaN frames*. Values between 1 and 6 denote user defined settings. A value of 10 corresponds to manual set positions. A value of 11 denotes excluded frames. A value of 12 is for interpolated frames.
Res.pD.final_nose_positions Nx2 array with final nose positions in each frame. The values are determined by the active settings within each frame. Excluded frames have NaN values.
Res.pD.final_body_positions Nx2 array with final body positions in each frame. The values are determined by the active settings within each frame. Excluded frames have NaN values.
Res.pD.final_mouse_angles 1xN array with final angles in each frame. The values are determined by the active settings within each frame. Excluded frames have NaN values.

Annotation data

Res.pD.annotations a structure array, with one element for each annotation event type as defined in the event definition file (**Appendix 7**). Each element is a sparse array of 1XN doubles, with N being the total number of session frames. Elements corresponding to frames associated with the event have a value of 1, and 0 otherwise.

Position data associated with each of the settings defined in the detection stage

This information is contained in the **position_results** a structure which is a field of **pD**, with one element for each detection setting. May of the fields are used in the parameter display. ***For each setting***, it includes the following fields:
Res.pD.position_results.mouseCOM Nx2 array with the X and Y coordinates of the mouse body for each frame in the session.
Res.pD.position_results.nosePOS Nx2 array with the X and Y coordinates of the mouse nose for each frame in the session.
Res.pD.position_results.GreyThresh 1XN array with the grey threshold used for each frame
Res.pD.position_results.TrimFact 1XN array with the trimming factor associated with each frame
Res.pD.position_results.MouseArea 1XN array with the mouse area in each frame
Res.pD.position_results.MousePerim 1XN array with the mouse perimeter in each frame
Res.pD.position_results.ThinMousePerim 1XN array with the mouse perimeter, after peeling, in each frame
Res.pD.position_results.mouse_angle: A 1XN array with mouse angles in each frame
Res.pD.position_results.mouse_length 1XN array with mouse lengths in each frame
Res.pD.position_results.MouseMean 1XN array with the mean intensity value of the mouse object in each frame

Res.pD.position_results.MouseRange 1XN array with the range of intensity values of the mouse object in each frame
Res.pD.position_results.MouseVar 1XN array with the variance of intensity values of the mouse object in each frame
Res.pD.position_results.BackGroundMean 1XN array with the mean intensity values of the rectangle containing the mouse object - the mouse pixels are excluded.

User defined and interpolated position data

Res.pD.user_defined_nosePOS NX2 array containing user defined mouse nose coordinates for those frames that are associated with user defined positions.
Res.pD.user_defined_mouseCOM NX2 array containing user defined mouse body coordinates for those frames that are associated with user defined positions.
Res.pD.user_defined_mouse_angle NX1 array containing user defined mouse angles for frames with user defined positions.
Res.pD.interpolated_nose_position NX2 array containing interpolated mouse nose coordinates for frames that have interpolated positions.
Res.pD.interpolated_body_position NX2 array containing interpolated mouse body coordinates for frames that have interpolated positions.
Res.pD.interpolated_mouse_angle NX2 array containing interpolated mouse angles for frames that have interpolated positions.

Arena data (an inherited structure array)

Res.pD.arena_data a structure containing information about the arena. It contains the following fields:
Res.pD.arena_data.videofilename full name of original video file for session
Res.pD.arena_data.arenafilename full name of arena file used for session
Res.pD.arena_data.user_string if defined, user entered string during session preparation
Res.pD.arena_data.OriginalImageSizeInPixels original image size in pixels. Width and height
Res.pD.arena_data.pixels_per_mm ratio of pixels to mm as specified during calibration
Res.pD.arena_data.frames_in_original_video first and last frames in original video for session
Res.pD.arena_data.MedianImage median image of arena
Res.pD.arena_data.FrameInfo Nx3 array of doubles with N being the total number of session frames. This array provides indices for each of the frames of the session into the block files created during session preparation. The index denotes the frame number, the first column is the block index, the second is the frame index within the block, and the third is the frame time within the session.
Res.pD.arena_data.arena_info structure with information about the specific arena definitions for the current session.

Information about settings used for detection

Res.pD.arena_data.detection_methods and **Res.pD.arena_data.detection_params** are structure arrays where one element for each of the different settings applied in the detection stage. Fields within these structures specify the entire set of detection parameters associated with each setting. Fields within detection methods are: name, algorithm, trimlevel, threshold,

median_background, user_background, no_background, mouse_brighter, mouse_darker, auto_determine_color, BackGroundImage, user_defined_params, user_defined_param_names, user_method_name, user_method_runstring.

Appendix 9. An example of custom analysis of data in the Results file

This appendix contains example code for analyzing freezing episodes in motion data. These examples are designed to show to load and process the data in the *results file*. The first block below includes a script that load a results data file, and calls the function **find_freezing_episodes**, and then plots the results. The second block below contains the function **find_freezing_episodes**. The two figures below show figures generated by the script, with different values for the min_pause_speed and min_pause_duration parameters. Note that some lines which are broken in the manual should appear as single lines in the MATLAB editor.

```
************************************
% Define a full name of a results file to load
% modify the XXX to an existing results file
% results file have the name '*result*.mat'
fname = 'XXX';

% Load the smooth speed variable
D = load(fname);
this_speed = D.Res.smoothed_speed;
deltaT = D.Res.deltaT; % frame sampling interval

% Define values defining a pause
max_pause_speed     = 0.5;  % cm / s
min_pause_duration_in_sec  = 5;
% minimal pause duration specified as number of frames
min_pause_duration  = round(min_pause_duration_in_sec/deltaT);

% Get a list of all freezing episodes - longest pause and total time paused
% all values are listed as frames numbers
[all_pauses,all_pause_durations,longest_pause,total_time_paused] =
find_freezing_episodes(this_speed,max_pause_speed,min_pause_duration);

figure;
plot(this_speed);
hold on
line([1 length(this_speed)],[max_pause_speed
max_pause_speed],'color','k','linestyle',':')
axis tight
xlabel('frame number')
ylabel('speed (cm/s)')
for i = 1:size(all_pauses,1)
    line([all_pauses(i,1) all_pauses(i,2)],[max_pause_speed
max_pause_speed],'color','r','linewidth',2);
end
title(['min pause duration: ' num2str(min_pause_duration) ' | max pause
speed: ' num2str(max_pause_speed) ' | total pauses time (frames): '
```

```
num2str(total_time_paused) ' num pauses: '
num2str(length(all_pause_durations))])
```

****

```
function [all_pauses,all_pause_durations,longest_pause,total_time_paused] =
find_freezing_episodes(speed,max_pause_speed,min_pause_duration)
% Find pauses in speed profile data
%
% INPUTS:
% speed: an array with speed values
% max pause speed: number indicating the maximum speed which counts as non
% movement
% minimum_pause_duration = minimum number of frames, required for a segment
% of non-movement
% to qualify as a pause
%
% OUTPUTS:
% all_pause_durations: a list of durations of all pauses
% all_pauses: a list of start and end time of all pauses
% longest_pause: the longest pause
% total_time_paused: the total time paused
% NOTE: all outputs returned as numbers of frames, not absolute time
%
% YBS February 2017

speed(isnan(speed)) = 0;

all_pauses        = [];
all_pause_durations  = [];
longest_pause     = 0;
total_time_paused = 0;

% Find all slow frames - this results in a vector of 0 and 1s
slowframes = speed <= max_pause_speed;

if isempty(slowframes)
    return
end

% find the starts and ends of pause segmenbts
pause_starts = 1 + find(diff(slowframes) ==  1);
pause_ends =   1 + find(diff(slowframes) == -1);

% pause index
pi = 1;
% if we started with a pause
if pause_ends(1) < pause_starts(1)
    if (pause_ends(1))  > min_pause_duration
        all_pause_durations(pi) = pause_ends(1);
        all_pauses(pi,:) = [1 pause_ends(1)];
        pi = pi + 1;
    end
    pause_ends = pause_ends(2:end);
end
% if we ended with a pause
if pause_starts(end) > pause_ends(end)
```

```
    if (length(speed) - pause_starts(end))  > min_pause_duration
        all_pause_durations(pi) = length(speed) - pause_starts(end);
        all_pauses(pi,:) = [pause_starts(end) length(speed)];
        pi = pi + 1;
    end
    pause_starts    = pause_starts(1:end-1);
end
% now they should be the same length
if ~(length(pause_starts) == length(pause_ends))
    disp('bug in the find freezing episodes function')
    return
end
for i = 1:length(pause_starts)
    this_start = pause_starts(i);
    tmp_ends = pause_ends(pause_ends > this_start);
    this_end   = min(tmp_ends);
    all_pause_durations(pi) = this_end - this_start;
    all_pauses(pi,:) = [this_start this_end];
    pi = pi + 1;
end

% keep only the pauses that are long enough
all_pauses = all_pauses(all_pause_durations >= min_pause_duration,:);
all_pause_durations = all_pause_durations(all_pause_durations >=
min_pause_duration);
longest_pause = max(all_pause_durations);
total_time_paused = sum(all_pause_durations);
```

****

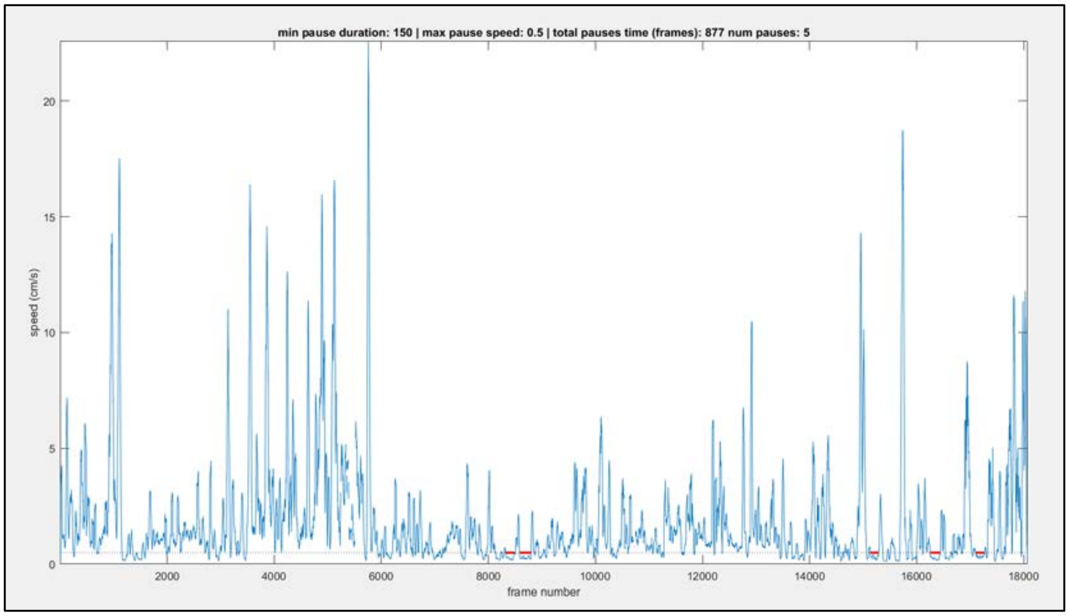

Figure A.5 Plot created by the script shown in Appendix 9.

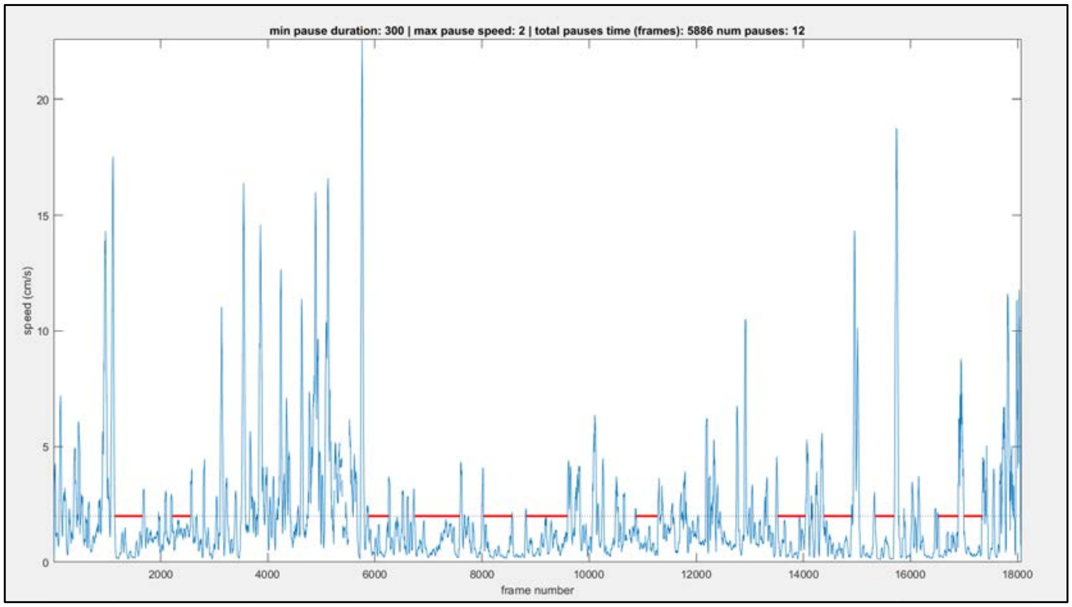

Figure A.6 **Another plot created by the script shown in Appendix 9, using different settings for the max pause speed, and the min pause duration.**

Appendix 10. A detailed explanation of OptiMouse detection algorithms.

All built in detection algorithms are implemented by the function **get_mouse_position_mm.** The function receives the arena image (one frame), the algorithm (default is 6), the number of trim cycles (default is 3) and the *greythreshold* factor (default is 0.5).

The MATLAB *graythresh* function is used to derive a threshold for the image. It then multiples the value by the user defined factor, specified as greythreshold (if it is set to 1, then the *graythresh* function's value will be used). The grayscale image is then converted to a binary (black and white) image using the MATLAB **im2bw** function and the threshold.

The connected components within the binary image are then found using the MATLAB **bwconncomp** function.

If the **bwconncomp** function does not return any object, the detection function will return with empty outputs. If multiple objects are found, then the object with the largest area will be considered.

Then, the MATLAB function **regionprops** is used to derive the following properties from the object: area, bounding box coordinates, centroid, list of object pixels, and list of pixels on the perimeter of the object. ***The mouse center of mass is the centroid of this object.***

Then, a smaller binary containing only the mouse object (not the entire arena) is created. It is named *mouse*. Note that merely clipping the original image according to the mouse bounding box is not sufficient, since it may also contain non mouse objects. Therefore, derivation of the *mouse* variable involves explicit assignment of 1's to the pixels that correspond to the mouse.

Next, the mouse image is peeled. The number of peeling cycles is determined by the *trim_cycle* argument to the main detection function. Prior to peeling, the original mouse is saved as *orig_mouse*. Peeling uses the OptiMouse function **trim_object_periphery**, which in turn uses the MATLAB function **bwperim** to find the pixels on the mouse object periphery. The result of peeling is saved in the variable *thin_mouse*.

The list of pixels on the perimeter of the original mouse is saved in the variable *orig_boundary*. This is found using the MATLAB **bwboundaries** function.

Using the MATLAB functions **bwconncomp** and **regionprops** the centroid, bounding box, and the perimeter of the thin mouse are derived.

The *tail* is found by subtracting the *thin_mouse* from the *orig_mouse*.
Note that *tail* will not only include the tail, but rather everything that was thinned. Nevertheless, the center of mass of the *tail* is closer to the center of mass of the actual tail than is the entire mouse center of mass.

The *tail* center of mass is found using the MATLAB functions **bwconncomp** and **regionprops**. If more or less than one object is found in the *tail* variable, the detection function will return.

The Euclidean distances of all the mouse's boundary points from the *tail* center of mass are found, and saved in the *distfromtail* variable.

The Euclidean distances of all boundary points from the tailless mouse center of mass are found and saved in the *distfrommouse* variable.

The *distfrommouse* is subtracted from the *distfromtail*, yielding the difference between the distances of each boundary point from the tail and from the body center of mass. This is saved in the variable *tailheaddist*.

At this stage, the algorithm splits depending on the detection method chosen by the user via the interface. We describe the position of each algorithm in turn. Algorithms 1 to 6 are increasingly more complex. Algorithm 7 is relatively simple. Note that derivation of body center of mass, as described above, is identical for all algorithms.

Algorithm 1: the nose is defined as the perimeter pixel which is associated with the maximal *tailheaddist*.

Algorithm 2: the nose is defined the perimeter pixel which is on the bounding box, and has the largest *tailheadist*. The rationale for this approach is that the nose is often a sharp part which opposes the bounding box.

Algorithm 3: the nose is the perimeter pixel which is furthest from the *tail* center of mass, but is also on the bounding box. It also must be further from the *tail* than from the *thin_mouse* center of mass.

Algorithm 4: the nose is defined the perimeter pixel which is furthest from the *tail end.* It also must be further from the *tail* than from the *thin_mouse* center of mass.

The tail end is found as the perimeter pixel which is furthest from the *tail* center of mass, but is also closer to the *tail* center of mass than it is to the body center of mass. After finding the tail end, the Euclidean distance of each periphery pixel from the tail end is found and saved in *distfromend* variable.

Algorithm 5: The nose is the furthest point from the tail base. This approach is appropriate since the tail might be curved or at an angle relative to the mouse body, and so the tail end might actually be close to the head (leading to failure of algorithm 4).

The *thin_mouse* periphery (without the tail) is found using the MATLAB **bwboundaries** function. It is saved in the variable *thinboundary*.

The *tail end* is detected as described in Algorithm 4.

The *tail base* is defined as the pixel, on the *thin_mouse* periphery, which is closest to the *tail end* (again, using the Euclidean distance).

The Euclidean distance of all perimeter points on the mouse are then found and saved in *distfromtailbase*.

Then, we find the *tailbaseheaddist* for each peripheral pixel. This is the difference between the distance from tail base and the distance to the mouse center of mass. The nose is defined as the pixel which is associated with the maximal difference, but must also be closer the mouse center of mass than to the *tail base*.

Algorithm 6: As in Algorithm 5, the nose is defined as the furthest peripheral pixel from the *tail base*. However, the *tail base* itself is derived using a different algorithm, which is appropriate also when the tail is curved.

The *tail end* is derived using the same procedure described in algorithms 4 and 5. Then, using the periphery of the original mouse (saved in a variable *origBW*), and the position of the *tail end* (saved in the variable *endMASK*), the geodesic distance of each peripheral pixel from the *tail end*, along the mouse periphery is found using the MATLAB function **bwdistgeodesic**.

However, to find the *tail base*, all pixels within the tail itself must be ignored. Pixels within the tailless mouse are found by considering the *thin_mouse*, after adding a thickness reduced following the peeling procedure. The algorithm then finds the pixel which is closest to the tail end (but is not part of the tail itself), and is on the tailless mouse periphery.

Having found the *tail base*, the nose is defined, as in the previous method, as the perimeter point which is furthest from it (using the Euclidean distance). The nose must also be closer to the mouse body center of mass than it to the tail center of mass.

Algorithm 7: This algorithm is appropriate when the mouse "has no tail" (or when the tail can be thresholded out of the image). As in Algorithm 4, the *tail end* is defined as the perimeter pixel which is furthest from the tail center of mass, but which is also closer to tail center of mass than it is to the head center of mass and is on the perimeter. This algorithm works without a tail, since the snout is often the thinnest part and peeling will first make the head, rather than the tail disappear.
